# Placental methylome reveals a 22q13.33 brain regulatory gene locus associated with autism

**DOI:** 10.1186/s13059-022-02613-1

**Published:** 2022-02-16

**Authors:** Yihui Zhu, J. Antonio Gomez, Benjamin I. Laufer, Charles E. Mordaunt, Julia S. Mouat, Daniela C. Soto, Megan Y. Dennis, Kelly S. Benke, Kelly M. Bakulski, John Dou, Ria Marathe, Julia M. Jianu, Logan A. Williams, Orangel J. Gutierrez Fugón, Cheryl K. Walker, Sally Ozonoff, Jason Daniels, Luke P. Grosvenor, Heather E. Volk, Jason I. Feinberg, M. Daniele Fallin, Irva Hertz-Picciotto, Rebecca J. Schmidt, Dag H. Yasui, Janine M. LaSalle

**Affiliations:** 1 Department of Medical Microbiology and Immunology, University of California, Davis, CA USA; 2 Perinatal Origins of Disparities Center, University of California, Davis, CA USA; 3 Genome Center, University of California, Davis, CA USA; 4 MIND Institute, School of Medicine, University of California, Davis, CA USA; 5 Department of Biochemistry and Molecular Medicine, School of Medicine, University of California, Davis, CA USA; 6 Department of Public Health Sciences, University of California, Davis, CA USA; 7 Department of Epidemiology, School of Public Health, University of Michigan, Ann Arbor, MI USA; 8 Department of Obstetrics and Gynecology, University of California, Davis, CA USA; 9 Department of Psychiatry and Behavioral Sciences, Davis, CA USA; 10 Department of Mental Health, Bloomberg School of Public Health, Johns Hopkins University, Baltimore, MD USA; 11 Wendy Klag Center for Autism and Developmental Disabilities, Bloomberg School of Public Health, Johns Hopkins University, Baltimore, MD USA

**Keywords:** Autism spectrum disorder, Epigenomics, Human genetics, Structural variants, DNA methylation, Prospective study, Placenta, Hypoxia, Neurodevelopment, Postmortem brain

## Abstract

**Background:**

Autism spectrum disorder (ASD) involves complex genetics interacting with the perinatal environment, complicating the discovery of common genetic risk. The epigenetic layer of DNA methylation shows dynamic developmental changes and molecular memory of in utero experiences, particularly in placenta, a fetal tissue discarded at birth. However, current array-based methods to identify novel ASD risk genes lack coverage of the most structurally and epigenetically variable regions of the human genome.

**Results:**

We use whole genome bisulfite sequencing in placenta samples from prospective ASD studies to discover a previously uncharacterized ASD risk gene, *LOC105373085*, renamed *NHIP*. Out of 134 differentially methylated regions associated with ASD in placental samples, a cluster at 22q13.33 corresponds to a 118-kb hypomethylated block that replicates in two additional cohorts. Within this locus, *NHIP* is functionally characterized as a nuclear peptide-encoding transcript with high expression in brain, and increased expression following neuronal differentiation or hypoxia, but decreased expression in ASD placenta and brain. *NHIP* overexpression increases cellular proliferation and alters expression of genes regulating synapses and neurogenesis, overlapping significantly with known ASD risk genes and *NHIP*-associated genes in ASD brain. A common structural variant disrupting the proximity of *NHIP* to a fetal brain enhancer is associated with *NHIP* expression and methylation levels and ASD risk, demonstrating a common genetic influence.

**Conclusions:**

Together, these results identify and initially characterize a novel environmentally responsive ASD risk gene relevant to brain development in a hitherto under-characterized region of the human genome.

**Supplementary Information:**

The online version contains supplementary material available at 10.1186/s13059-022-02613-1.

## Background

Autism spectrum disorders (ASD) are growing in prevalence, with 1 in 54 children diagnosed in the USA [1]. Diagnosis of ASD is based on a child’s behavioral difficulties in social communication and interactions, language deficits, restricted interests and repetitive behaviors, and sensory sensitivities. The etiology of ASD is complex and heterogeneous, and it is likely to involve multiple genetic and environmental factors, as well as poorly understood gene-environment interactions [2–4]. Twin and sibling studies have shown a strong heritability of ASD risk within families, and most genetic risk for ASD is expected to come from common variants [5]. Exome sequencing of ASD trios has identified genes with rare mutations in ASD children, which are enriched for neuronal, embryonic development, and chromatin regulation functions, but no single gene explains more than 1% of disease risk [6, 7]. A large genome-wide association study (GWAS) identified individual genetic variants that contribute to an individual’s ASD risk and showed that the weighed sum of the risk alleles and their effect sizes can be combined to create a polygenic risk score (PRS) for ASD [8]. The variance explained for ASD using this approach was 2.45%, which was further improved to 3.77% by including in the prediction model additional PRS for traits co-heritable with ASD, including schizophrenia, depression, and educational attainment [8–10]. Common polygenic risk may also interplay with early environmental and perinatal factors in other neurodevelopmental disorders. For example, a PRS derived from a set of 108 previously identified genome-wide significant variants for schizophrenia [11] was shown to be significant only in the presence of early-life maternal complications (ELCs), and the subset of variants interacting with ELCs also corresponded with patterns of placental gene expression, consistent with the importance of placental gene regulation as a window into neurodevelopment [12]. However, most ASD genetic or environmental studies have not included placental molecular measures, despite the potential convergence between placental biology and genetic risk for ASD. Term placenta is an accessible tissue normally discarded at birth; however, the convergence between placental biology and genetic risk for ASD is relatively unexplored.

Placenta maintains a distinct landscape of DNA methylation characterized by partially methylated domains (PMDs), which is more similar to oocytes and preimplantation embryos, where methylation over gene bodies is positively correlated with expression, compared with the high overall methylated pattern of fetal or adult tissues [13–15]. In this way, placental methylation patterns are also similar to most epithelial-derived tumors, where PMDs are also found [16]. Both placenta and tumors show a strong responsiveness to hypoxia that promotes cell proliferation and angiogenesis, which are both required for invasion of the placental trophoblasts into the uterine decidua during placentation [17]. In vivo, hypoxia and reactive oxygen species promote neurogenesis in embryos, newborns, and adults, and also play a role in neuronal differentiation in vitro [18]. Furthermore, exposure to most environmental pollutants produces excessive oxidative stress that can impact both placentation and neurodevelopment [19].

Because of its multiple roles in support of fetal development during intrauterine life, the placenta is a promising tissue for identifying DNA methylation alterations at genes relevant to fetal brain and gene-environment interactions in ASD [20–23]. Most epigenome-wide association studies (EWAS) for ASD have used array-based methods to assess DNA methylation which lack coverage over the most epigenetically and genetically polymorphic regions of the human genome, such as correlated regions of systemic interindividual variation (CoRSIVs) and structural variants (SVs) [24]. CoRSIVs, which are regions of the genome with similar DNA methylation patterns across tissue types, are sensitive to periconceptional environment, observed across diverse tissues, associated with human disease genes, and are enriched for transposable elements and subtelomeric locations [24, 25]. SVs arising from transposable elements have been associated with many human phenotypes, especially immune responses and neuropsychiatric disorders, such as schizophrenia [26–28]. SVs exhibit a nonrandom distribution in hotspots within relatively gene-poor regions in primate genomes, but are enriched for gene functions in oxygen transport, sensory perception, synapse assembly, and antigen-binding [29, 30]. Recent studies suggested that a large SV burden was associated with lower cognitive ability [31–33] and ASD [34], but most GWAS and EWAS studies ignore SVs and CoRSIVs in the genome. Therefore, the combination of utilizing the unique placental DNA methylation landscape reflective of in utero gene expression with sequencing-based epigenome-wide investigations inclusive of understudied genomic regions is warranted.

Here, we investigated the association of ASD risk with placental DNA methylation in two high-risk familial ASD cohorts through whole genome bisulfite sequencing (WGBS) analysis of 204 individuals. We identified a block of differential methylation in ASD at 22q13.33, a region previously described as a CoRSIV and SV hotspot but not previously associated with ASD. A novel gene *LOC105373085* (renamed as *NHIP* for neuronal hypoxia inducible, placenta associated) within 22q13.33 was demonstrated to be expressed in brain, responsive to oxidative stress, and to influence expression of other known ASD-risk genes. A common SV insertion within 22q13.33 was associated with increased ASD risk, reduced expression of *NHIP*, and reduced methylation, but first month prenatal vitamin use counteracted this effect. Together, these results demonstrate a novel ASD risk gene regulatory locus at the interface of common genetics and perinatal environmental resilience.

## Results

### Differential methylation analysis using WGBS identifies a hypomethylated block at 22q13.33 in ASD placenta

**Fig. 1:**
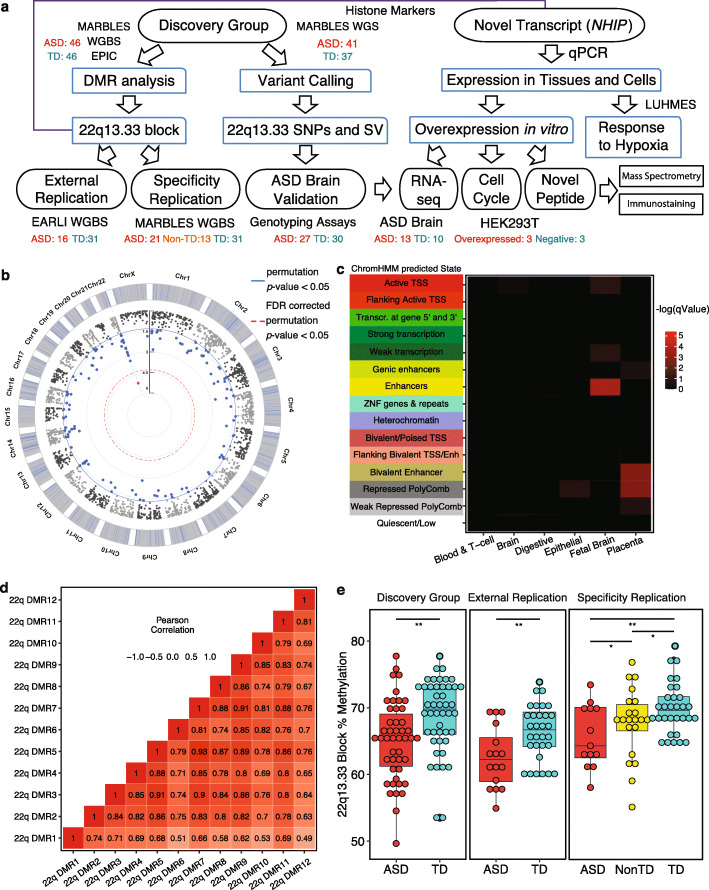
ASD-associated DMRs are enriched at fetal brains enhancers and a co-methylated block at 22q13.33 replicates across studies and platforms. **A** Schematic of the experimental design for discovery of ASD DMRs, replication of the co-methylated 22q13.33 locus, genetic associations, and functional follow-up of a novel gene (*NHIP*). **B** Circular Manhattan plot of the epigenome-wide association of DNA methylation in placenta with ASD diagnosis at 36 months. Results are represented as DMR association test results (− log_10_(*p*)). Significant thresholds are blue for permutation *p* value < 0.05, red for FDR-adjusted permutation *p* value < 0.05, and gray for nonsignificant. **C** 134 ASD DMRs (permutation *p* value < 0.05) tested for enrichment within chromatin states defined by Epigenome Roadmap ChromHMM [35]. Each row represents a different ChromHMM state and each column a single tissue type, with the heatmap plotting the − log_10_(*q*-value) significance of ASD DMR enrichment. **D** Correlation matrix of methylation levels using the Pearson correlation coefficient for the 12 DMRs located in the 22q13.33 hypomethylated block. **E** Smoothed methylation values were averaged over the 22q13.33 hypomethylated block (*y*-axis) and compared across diagnosis groups (*x*-axis). In the discovery group, ASD samples had significantly lower methylation than TD samples (MARBLES, HiSeq X, ASD *n* = 46, TD *n* = 46) (*p* value = 0.002). The same result and direction were observed in the external replication group (EARLI, HiSeq 2500, ASD *n* = 16, TD *n* = 31) (*p* value = 0.009). For the specificity replication group (MARBLES, NovaSeq, ASD n = 21, Non-TD *n* = 13, TD *n* = 31), ASD methylation levels were also significantly lower than both TD (*p* value = 0.005) and Non-TD (*p* value = 0.049), while Non-TD was significantly lower than TD samples (*p* value = 0.050) by Mann-Whitney-Wilcoxon. Box plot center lines, box limits, and whiskers represented median, interquartile range, and minimum and maximum values, respectively

To identify novel regions of epigenetic alterations in placenta discriminating later ASD diagnosis, we performed WGBS analysis of genome-wide DNA methylation on 204 subjects from two prospective high-risk ASD cohorts (MARBLES and EARLI) with a diagnosis outcome at 36 months (Fig. 1a). Subject clinical and demographic information as well as cell type proportions and global methylation measurements are provided in Additional File 1: Table S1, while data on individual sample sequencing information is in Additional File 2: Table S2. No demographic, cell type, or technical variables were significantly associated with ASD outcome, but scores related to ASD severity and cognition were associated, as expected (FDR-corrected and raw *p* values from Fisher’s exact for categorical or one-way ANOVA for continuous variables is in Additional File 1: Table S1). Global methylation levels over 20-kb windows were also not different by diagnostic group (Additional File 3: Fig. S1). Since sequencing platform differences (Illumina HiSeq 4000, HiSeq X, NovaSeq) impacted global methylation levels (Additional File 3: Fig. S2), we separated the samples into “discovery,” “external replication,” and “specificity replication” groups for initial analyses of differentially methylated regions.

Differentially methylated regions (DMRs) distinguishing ASD from typical development (TD) placental samples were identified with a permutation-based statistical approach, adjusted for sex and placental cell types, to identify broad epigenomic signatures of multiple gene regulatory regions at a genome-wide level in the discovery group (Additional File 2: Table S2). In total, 134 DMRs (permutation *p* value < 0.05) representing an average size of 1027 bp with 5–10% smoothed methylation differences, including 77 hyper- and 57 hypomethylated in ASD compared to TD, mapped to 183 genes (Fig. 1b, DMR characteristics and gene names are in Additional File 4: Table S3). A cluster of 12 ASD DMRs mapped to 22q13.33, all hypomethylated in ASD (5-7% difference from TD), including one that also passed genome-wide significance (FDR-adjusted *p* value < 0.05). Methylation levels within the 134 ASD DMRs were specifically associated with autism severity and cognitive scores, but not other demographic and technical variables after adjustment in a linear model (Additional File 3: Fig. S3). Further evidence that DMRs identified in placenta reflect epigenetic differences relevant to brain and development came from the significant enrichment of ASD DMRs in fetal brain enhancers, as well as bivalent enhancer and repressed polycomb regions of placenta compared to background regions using ChromHMM-defined chromatin states from the Roadmap Epigenomics Project [35] (Fig. 1c, detailed information on each tissue type and chromatin state is in Additional File 5: Table S4). Demonstrating their functional relevance, hyper-methylated ASD DMRs were enriched within 0–5-kb and 5–50-kb windows downstream of transcription start sites (TSS), at CpG islands and shores for both hyper- and hypomethylated DMRs (Additional File 3: Fig. S4-S5), and at known transcription factor binding sites (names, motifs, and enrichment statistics are in Additional File 6: Table S5). Genes mapping to placental ASD DMRs significantly overlapped with ASD risk genes from the Simons Foundation Autism Research Initiative (SFARI) dataset compared to all expressed genes (Fisher’s exact test, *p* value = 0.006, odds ratio (OR) = 2.321) [36] (Venn diagram in Additional File 3: Fig. S6, overlapping gene names and DMR characteristics are in Additional File 7: Table S6). The overrepresentation of 12 DMRs at 22q13.33 hypomethylated in ASD drove the additional enrichment at > 500 kb of TSS as well as gene ontology (GO) enrichment for functions in histone acetyltransferase (HATs) and chromatin modification, due to the assignment of the 22q13.33 hypomethylated DMRs to the nearest downstream gene *BRD1*, a histone acetyltransferase (top Biological Process and Cellular Compartment GO terms are graphed in Additional File 3: Fig. S7, GO terms and enrichment statistics are in Additional File 8: Table S7). Based on the genome-wide significance and observation of multiple DMRs mapping to the same genomic region, we decided to focus subsequent analyses on further understanding the impact of the 22q13.33 hypomethylated locus on ASD risk.

The 22q13.33 DMRs hypomethylated in ASD were highly positively correlated with each other and formed a 118-kb hypomethylation cluster that was also detected as a hypomethylated block (chr22: 49044669 - 49162642, hg38) (Fig. 1d, plot of methylation values of individual samples is in Additional File 3: Fig. S8). This chromosomal locus was also previously described as a CoRSIV, a region of correlated methylation differences between individuals [24]. We therefore examined smoothed methylation levels over the 118 kb 22q13.33 block for replication in a different ASD enriched risk cohort (EARLI, external replication group). Similar to the discovery group (Mann-Whitney-Wilcoxon, *p* value = 0.002, ASD *n* = 46, TD *n* = 46, effect size = −0.645), 22q13.33 block methylation levels were significantly lower in ASD compared to TD (Mann-Whitney-Wilcoxon, *p* value = 0.009, ASD *n* = 16, TD *n* = 31, effect size = −0.867) (Fig. 1e, comparisons of individual DMRs within the 22q13.33 block are in Additional File 9: Table S8). Furthermore, an independent “specificity replication group” of MARBLES subjects that included a “Non-TD” diagnostic group sequenced on a different platform also showed significantly lower 22q13.33 DNA methylation levels in ASD compared to either TD or the additional diagnostic Non-TD samples, defined as atypical cognitive scores but not ASD (Mann-Whitney-Wilcoxon, ASD vs TD *p* value = 0.005, effect size = −0.991, ASD vs Non-TD *p* value = 0.049, effect size = −0.282, Non-TD vs TD *p* value = 0.050, ASD *n* = 21, Non-TD *n* = 13, TD *n* = 31, effect size = −0.452) (Fig. 1e, Additional File 9: Table S8). Smoothed methylation levels over the 118-kb 22q13.33 block remained significantly associated with ASD after adjustment for 18 potential covariates in a linear model in the discovery group (Additional File 3: Fig. S3c) and after adjustment for two nominal covariates in all three groups (Additional File 3: Fig. S9 and Additional File 10: Table S9). These results demonstrate that hypomethylation over the 118 kb 22q13.33 co-methylated block is a reproducible finding across different cohorts and platforms, specifically distinguishing placental samples of newborns later diagnosed with ASD.

### *NHIP* is a primate-specific gene dynamically expressed during neuronal differentiation that exhibits reduced expression in ASD

**Fig. 2:**
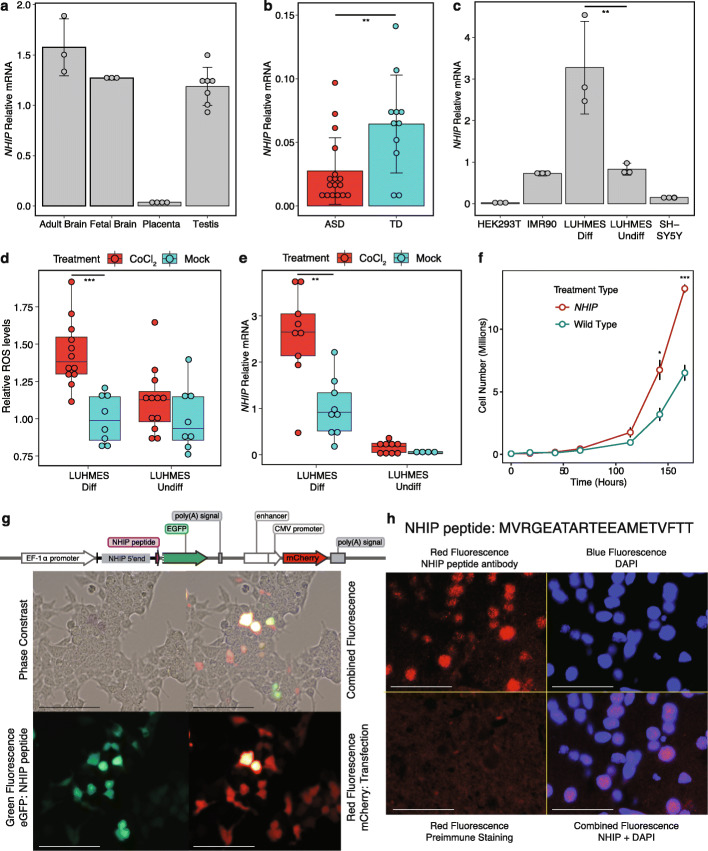
Functional characterization of *NHIP* transcript levels in response to neuronal hypoxia. In **a**–**e** RT-qPCR assays, *NHIP* levels were normalized to *GAPDH* with at least three independent experiments per condition. **A** *NHIP* levels in human tissues, including adult brain, fetal brain, placenta, and testis. **B** *NHIP* levels in placenta samples from the discovery group (ASD *n* = 17, TD *n* = 11). ASD samples show significantly lower *NHIP* levels than TD samples (Mann-Whitney-Wilcoxon, *p* value = 0.009). **C** *NHIP* levels in human cell lines, HEK293T, IMR90, LUHMES, and SH-SY5Y. In LUHMES cells, *NHIP* levels were significantly higher in differentiated neurons compared to undifferentiated neurons (Mann-Whitney-Wilcoxon, *p* value = 0.034). **D** Differentiated LUHMES cells are more sensitive to hypoxia than undifferentiated LUHMES cells. Formation of reactive oxygen species (ROS) was measured in differentiated and undifferentiated LUHMES cells treated with 100 nM CoCl_2_, a hypoxia mimetic, or vehicle (mock) (Mann-Whitney-Wilcoxon, *p* value = 0.0001). **E** *NHIP* levels increase in response to hypoxia, specifically in differentiated neurons. Differentiated or undifferentiated LUHMES cells were treated with 100 nM CoCl_2_. In differentiated LUHMES cells, CoCl_2_ treatment significantly increased *NHIP* levels (Mann-Whitney-Wilcoxon, *p* value = 0.009). **F** *NHIP* overexpression in HEK293T cells resulted in a faster doubling time than vector control cells, indicating increased cell proliferation. (Hour 142, Mann-Whitney-Wilcoxon, *p* value = 0.045, *NHIP* overexpression cells *n* = 3, control cells *n* = 3, effect size = 3.10; Hour 166, Mann-Whitney-Wilcoxon, *p* value = 0.0009, *NHIP* overexpression cells *n* = 3, control cells *n* = 3, effect size = 7.80). **G** Vector design of NHIP peptide-eGFP (dotted line represents excised ATG of EGFP) and combined phase and fluorescent microscopy. Green, eGFP linked to NHIP peptide; red, mCherry, transfection positive control. Scale bars, 100 μm. **H** Immunofluorescent staining of human frontal cortex, showing nuclear localization with anti-NHIP, but not pre-immune control. Blue, DAPI nuclear counterstain; red, anti-NHIP staining. Scale bars, 100 μm. Data are mean ± SEM

The 22q13.33 co-methylated block was within an apparent gene desert, located more than 500 kb away from the closest annotated protein coding genes: *FAM19A5 (TAFA5)* and *BRD1*. Epigenetic evidence for promoter and enhancer activity within 22q13.33 was obtained from placenta, ovary, and brain ENCODE datasets (Additional File 3: Fig. S10) [37]. Within 22q13.33, an active promoter peak identified by H3K4me3 histone markers was observed in a subset of ovary, placenta, and brain samples, suggesting variable promoter marks between individuals (Additional File 3: Fig. S11). This H3K4me3 peak overlapped a CpG island and the TSS of the uncharacterized transcript, *LOC105373085* (also named *AK057312*) identified from a human testis cDNA library (Additional File 3: Fig. S11) [38]. We renamed *LOC105373085* as *NHIP*, for *neuronal hypoxia inducible*, *placenta associated*. *NHIP* is also variably expressed among brain regions from the Genotype-Tissue Expression (GTEx) database (Additional File 3: Fig. S12) [39]. The full-length *NHIP* sequence is syntenic in all primates, but not in other vertebrates including mouse (Additional File 3: Fig. S13, conservation scores for individual species are in Additional File 11: Table S10). When quantified by RT-PCR in human tissues, *NHIP* was expressed in placenta, testis, and adult and fetal brain, with relatively lower expression in placenta (Fig. 2a). ASD placental samples showed significantly lower *NHIP* transcript levels than TD samples, in the same direction as methylation changes in the 22q13.33 block (Mann-Whitney-Wilcoxon, *p* value = 0.009, ASD *n* = 17, TD *n* = 11, effect size = −1.12, Fig. 2b). In placenta, because of the epigenetic feature of PMDs, gene body methylation is positively associated with and predicts active gene expression across mammals [13, 15]. Since the 22q13.33 co-methylated block mapped to a previously defined PMD in placenta [15] (map of region is in Additional File 3: Fig. S14), these results suggest that hypomethylation of the 22q13.33 block in ASD is reflective of lower past or current expression of *NHIP* expression in utero for ASD compared to TD.

To understand the function of this uncharacterized gene, we assayed and detected levels of *NHIP* expression in four human cell lines selected for their early-life origins (HEK293T, IMR90, LUHMES, SH-SY5Y). All cell lines are derived from human females; HEK293T cells are of embryonic kidney origin, IMR90 are fetal fibroblasts, Lund human mesencephalic (LUHMES) are derived from embryonic human mesencephalon, and SH-SY5Y cells are from a neuroblastoma. A significant increase in *NHIP* transcript levels was observed following neuronal differentiation in LUHMES cells (Mann-Whitney-Wilcoxon, *p* value = 0.034, differentiated LUHMES *n* = 3, TD undifferentiated LUHMES *n* = 3, effect size = 3.07, Fig. 2c). Since both neuronal differentiation and placental trophoblast differentiation respond to hypoxic conditions and oxidative stress in response to environmental pollutants [40, 41], we tested the responsiveness of *NHIP* to hypoxia. Differentiated LUHMES neurons were more sensitive to treatment with a hypoxia mimetic (CoCl_2_) than undifferentiated cells, with a significant decrease in cell viability and an increase in reactive oxygen species (ROS) levels (Mann-Whitney-Wilcoxon, *p* value = 0.0001, differentiated LUHMES treated with 100 nM CoCl_2_ *n* = 12, differentiated LUHMES treated with vehicle *n* = 8, effect size = 2.26, Fig. 2d, H_2_O_2_ results and statistics are in Additional File 3: Fig. S15). *NHIP* transcript levels also increased after exposure to CoCl_2_ specifically in differentiated, but not undifferentiated LUHMES cells (Mann-Whitney-Wilcoxon, *p* value = 0.009, differentiated LUHMES treated with 100 nM CoCl_2_ *n* = 9, differentiated LUHMES treated with vehicle *n* = 9, effect size = 1.56, Fig. 2e). Following removal of hypoxia, *NHIP* transcript levels returned to untreated levels, demonstrating the transience of the response (Additional File 3: Fig. S16). Among the tested human cell lines, embryonic kidney origin HEK293T cells had the lowest endogenous *NHIP* transcript levels (Fig. 2c). Since response to hypoxia is a developmental signal regulating cell proliferation in embryos [42], we experimentally tested this hypothesis by transiently transfecting HEK293T cells with either a plasmid encoding *NHIP* with a dual GFP-Puromycin selection cassette or a control vector control lacking *NHIP* (plasmid construct shown in Additional File 3: Fig. S17, sequences of vector and peptide are in Additional File 12: Table S11). A significantly shortened doubling time was observed in response to *NHIP* overexpression compared to control cells (20.23 h vs. 24.91 h) (Hour 142, Mann-Whitney-Wilcoxon, *p* value = 0.045, *NHIP* overexpression cells *n* = 3, control cells *n* = 3, effect size = 3.10; Hour 166, Mann-Whitney-Wilcoxon, *p* value = 0.0009, *NHIP* overexpression cells *n* = 3, control cells *n* = 3, effect size = 7.80, Fig. 2f, Additional File 3: Fig. S18). These results demonstrate that *NHIP* is a hypoxia-inducible gene in neurons that regulates cell proliferation in an embryonic cell line with low endogenous expression.

To examine whether *NHIP* encoded a protein, we identified a 20-amino acid (aa) putative peptide containing a Kozak sequence and tested the existence of the peptide by designing the NHIP peptide-eGFP vector so that the peptide sequence would be in frame with ATG-less GFP transfected in HEK293T cells (Fig. 2g, sequences are in Additional File 12: Table S11). The presence of both transfection control (red, mCherry) and reporter (green, eGFP) confirmed the existence of the 20 aa NHIP peptide (Fig. 2g). The *NHIP* encoded peptide sequence was confirmed using mass spectrometry after pull-down with anti-GFP antibody (Additional File 3: Fig. S19). A search of human protein databases demonstrated that the *NHIP* peptide partially overlapped protein sequences within BRCA2 and CHD4 (alignments are shown in Additional File 3: Fig. S20, blastp results are in Additional File 13: Table S12). Lastly, using a custom antibody against the *NHIP* encoded peptide, immunostaining was performed on sections of human postmortem prefrontal cortex, demonstrating nuclear staining in a subset of neuronal nuclei (Fig. 2h). Together, these results demonstrate the existence of a nuclear peptide encoded by *NHIP*.

### A common genetic structural variant at 22q13.33 is associated with reduced placental DNA methylation, reduced *NHIP* expression, and increased ASD risk

**Fig. 3:**
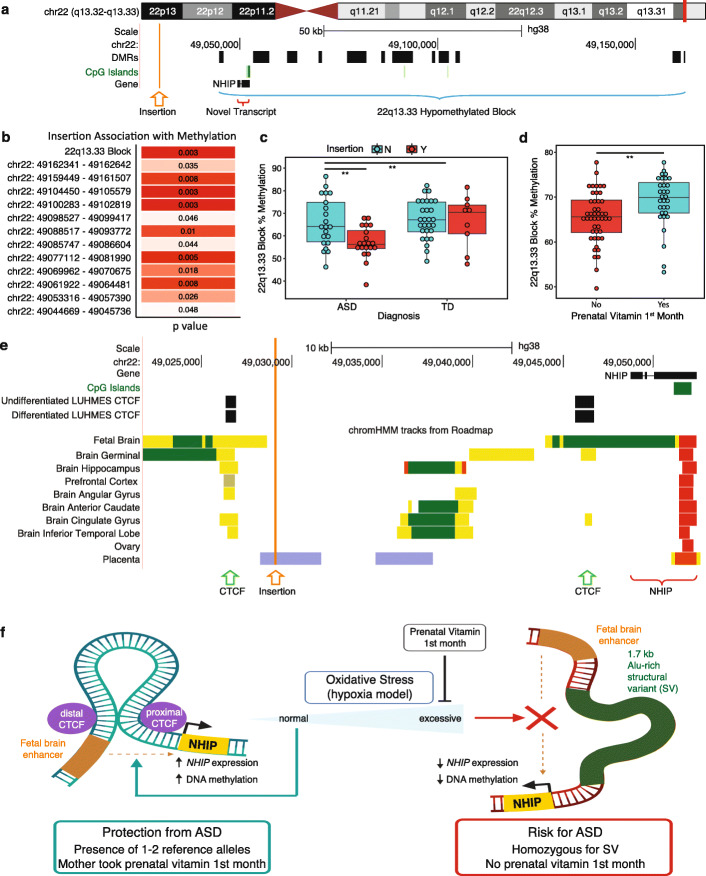
A common genetic structural variant is significantly associated with 22q13.33 DNA methylation and ASD. **A** Insertion location (orange) relative to the 22q13.33 hypomethylated block (blue), and the novel transcript, *NHIP* (red) in the UCSC genome browser. The 22q13.33 co-methylated block was 117,974 bp in length (blue). *NHIP* TSS was located 7881 bp downstream from the start of the 22q13.33 hypomethylated block. The insertion (not in the reference genome) is 15,013 bp upstream from the start of the 22q13.33 hypomethylated block. **B** The association matrix shows ANOVA *p* values for the comparison of the insertion genotype (homozygous for insertion versus not) with smoothed methylation levels within each of 12 DMRs located in the 22q13.33 hypomethylated block from the discovery group (ASD *n* = 41, TD *n* = 37). **C** Association was tested between insertion genotype (Y, homozygous for insertion; N, not) and 22q13.33 co-methylated block methylation levels (discovery group, ASD *n* = 41, TD *n* = 37). ASD showed significantly lower DNA methylation levels compared to TD placenta samples within the entire 22q13.33 co-methylated block (Mann-Whitney-Wilcoxon, *p* value = 0.008, ASD *n* = 41, TD *n* = 37, effect size = −0.645). Samples homozygous for the insertion had significantly lower methylation than those not having insertion on one or both alleles (Mann-Whitney-Wilcoxon, *p* value = 0.004, Y *n* = 29, N *n* = 49, effect size = − 0.644). When broken down by diagnosis, samples with insertion had significantly lower methylation specifically in ASD samples (Mann-Whitney-Wilcoxon, *p* value = 0.005, Y *n* = 20, N *n* = 21, effect size = −0.878), not TD samples (Mann-Whitney-Wilcoxon, *p* value = 0.847, Y *n* = 9, N *n* = 28, effect size = −0.173). **D** Periconceptional prenatal vitamin use was a significant modifier of 22q13.33 block methylation in placenta (discovery group, ASD *n* = 41, TD *n* = 37). Lower percent methylation at the 22q13.33 co-methylated block was significantly associated with not taking prenatal vitamins during the first month of pregnancy (Mann-Whitney-Wilcoxon, *p* value = 0.001), which was in the same direction as ASD risk. **E** UCSC genome browser map shows the insertion location (orange vertical line) relative to two adjacent CTCF sites (green arrows) and *NHIP*. Both undifferentiated and differentiated LUHMES cells have both CTCF sites, consistent with them being homozygous for the reference sequence. Additional brain tracks show the variability of the upstream CTCF site between human samples. ChromHMM tracks were derived from fetal brain, multiple brain regions, ovary, and placenta. Red, active promoter; yellow, active enhancer; green; active transcriptional elongation; purple, bivalent poised chromatin. **F** Working model to explain ASD risk associated with SV homozygosity. Illustrations created with BioRender.com

To examine genetic factors associated with 22q13.33 methylation levels and polymorphic expression of *NHIP* in ASD, we tested the association between 22q13.33 block DNA methylation levels and common variants from individual-matched whole genome sequencing (WGS), including SNPs, insertions or deletions (indels), copy number variations (CNVs), and SVs. Methylation levels in five out of 12 ASD DMRs within 22q13.33 were significantly associated with common SNPs located inside the DMRs (linear regression, *p* values in Additional File 3: Fig. S21). A 1674 bp SV insertion (chr22: 49029657, hg38) was identified 15,013 bp upstream of the start of the 22q13.33 co-methylated block (Fig. 3a, genotypes and demographics of each subject are in Additional File 14: Table S13) with which DNA methylation levels of all 12 22q13.33 ASD DMRs were significantly associated (linear regression, *p* values are in Fig. 3b). In the MARBLES cohort, this SV insertion was identified in significantly more ASD than TD samples (chi-square test, ASD *n* = 41, TD *n* = 37, *p* value = 0.045) (Additional File 3: Fig. S22). Placenta samples with the 22q13.33 insertion from ASD, but not TD, showed significantly lower methylation levels (methylation vs diagnosis: Mann-Whitney-Wilcoxon, *p* value = 0.008, ASD *n* = 41, TD *n* = 37, effect size = −0.645; methylation vs genotype: Mann-Whitney-Wilcoxon, *p* value = 0.004, Y *n* = 29, N *n* = 49, effect size = −0.644; in ASD samples: methylation vs genotype: Mann-Whitney-Wilcoxon, *p* value = 0.005, Y *n* = 20, N *n* = 21, effect size = −0.878; in TD samples: methylation vs genotype Mann-Whitney-Wilcoxon, *p* value = 0.847, Y *n* = 9, N *n* = 28, effect size = −0.173; Fig. 3c). While not present in the reference genome, the 22q13.33 insertion was also identified as a structural variant identified from PacBio assembly data of the human CHM1 complete hydatidiform cell line (CHM1_chr22-49029645-INS-1673 contig [43] and NCBI GenBank ID QPKN01007947.1 [44] Additional File 3: Fig. S23). We also confirmed the WGS identification of the SV using PCR genotyping primer sets and provided allelic genotypes (genotypes are in Additional File 14: Table S13, example of PCR products is in Additional File 3: Fig. S24). The insertion sequence showed high similarity with retrotransposon elements, including SVA and Alu (Additional File 3: Fig. S25). This 22q13.33 SV also corresponded to INS_22_115103 in Genome Aggregation Database (gnomAD) which showed an average allele frequency of 0.7 and only small deviation (10% or less) based on ancestry or sex (Additional File 3: Fig. S26) [45, 46].

Since the 22q13.33 block exhibited lower methylation in ASD compared to TD placental samples from the MARBLES cohort, we chose to evaluate the relationship between prenatal vitamin use during the first month of pregnancy, previously shown to be associated with decreased ASD risk in this cohort [47], in the general population [48, 49], and in other high-familial risk cohorts [50], in the context of ASD risk associated with the insertion. Along with other vitamins and minerals, prenatal vitamins contain high folic acid to meet the increased needs during pregnancy, and could be an important source of methyl donors. There was a significant positive association with prenatal vitamins use in the first month and methylation level at the 22q13.33 block, in the protective direction (Mann-Whitney-Wilcoxon, *p* value = 0.001, took prenatal vitamin *n* = 31, did not take prenatal vitamin *n* = 46, effect size = 0.477) (Fig. 3d). When samples were stratified by 22q13.33 insertion genotype, prenatal vitamin use during the first month of pregnancy showed a nominally significant protective effect among individuals with the insertion, although we acknowledge that the sample size is small (within samples homozygous for the insertion: in samples from mothers who took prenatal vitamin during the first month of pregnancy, Mann-Whitney-Wilcoxon, *p* value = 0.046, ASD *n* = 3, TD *n* = 4, effect size = −2.09; Additional File 3: Fig. S27). Unlike the 22q13.33 insertion, the GWAS-based PRS [8] calculated for the MARBLES cohort was not significantly different between diagnostic groups or associated with 22q13.33 block methylation by ANOVA in the MARBLES discovery cohort (Additional File 15: Table S14). Together, these results are consistent with the hypothesis that ASD risk associated with the 22q13.33 SV and co-methylated block is distinguishable from polygenic ASD risk and tempered by a common nutrient intervention with evidence for an association with ASD protection.

Since SVs have been previously implicated in altering chromatin loops regulating promoter-enhancer interactions [51], we hypothesized that this 1.7 kb insertion may be located within an enhancer-promoter loop relevant to fetal brain. Using the recent EpiMap database of chromatin states across multiple humans and tissue types [52], we identified two CTCF sites flanking the SV insertion (Fig. 3e). ChromHMM maps [35] demonstrate a fetal brain enhancer that aligns with the distal CTCF binding site. The proximal CTCF site is adjacent to the *NHIP* TSS, which ChromHMM predicts as an active promoter in brain, ovary, and placenta. These two CTCF binding sites were inside a large ~ 2 Mb topologically associated domain (TAD) spanning from the 48.5 Mb position to the telomere of 22q (Additional File 3: Fig. S28) [53]. Together, these results suggest a model whereby the SV insertion allele could disrupt the fetal brain enhancer-promoter interaction within a large telomeric TAD, thereby reducing the responsiveness of *NHIP* expression to neuronal differentiation and excessive oxidative stress (Fig. 3f). Early pregnancy prenatal vitamin use is expected to counteract the effects of oxidative stress through provision of dietary methyl groups, thereby increasing DNA methylation at the *NHIP* locus in individuals homozygous for the 22q13.33 insertion.

### *NHIP* expression is reduced in ASD brain and associated with the regulation of genes enriched for synaptic functions and ASD risk

**Fig. 4:**
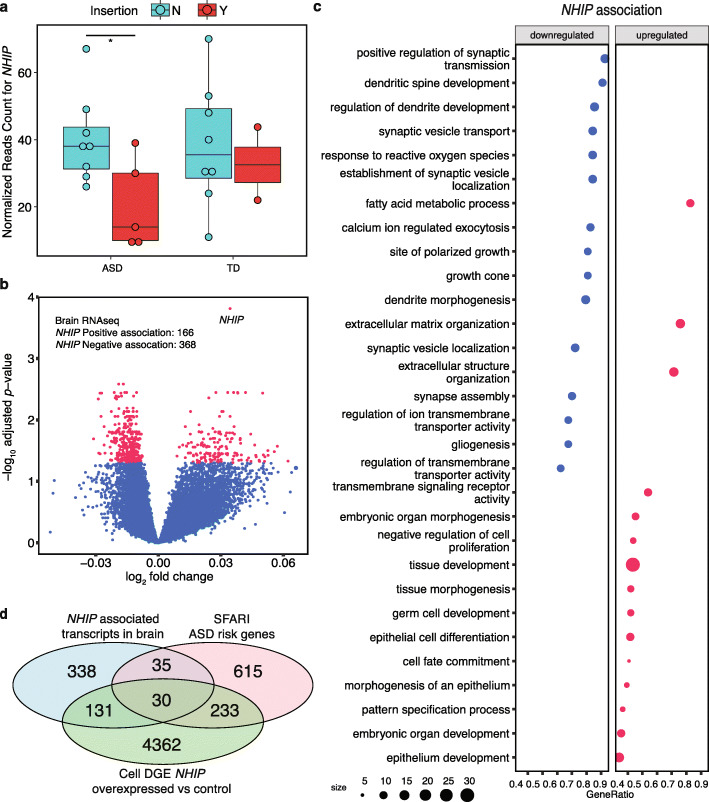
*NHIP* levels in brain are reduced in ASD and associated with expression of genes enriched for synaptic functions, response to oxidative stress, and ASD risk. **A** Brain samples homozygous for the 22q13.33 insertion had significantly lower *NHIP* levels compared to those who were not (*p* value = 0.048). The association between *NHIP* levels and the insertion was observed specifically in ASD (*p* value = 0.036), not in TD (*p* value = 0.711) (Mann-Whitney-Wilcoxon, brain, ASD *n* = 13, TD *n* = 10). **B** *NHIP-*associated differential expression analysis was performed from brain RNA-seq, adjusted for sex, age, brain region, and PMI, identifying 534 genome-wide significant genes (FDR-adjusted *q*-value < 0.05). **C** Gene ontology (GO) enrichment analysis of the 851 *NHIP-*associated genes in brain identified significantly enriched terms (FDR-adjusted *q*-value < 0.05). Positively associated GO terms are shown in red and negatively associated GO terms are colored in blue. **D** Venn diagram representing the 30 genes in common between *NHIP* association in brain (adjusted), differential gene expression (DGE) in the *NHIP* overexpressed cell line, and SFARI ASD risk genes. Genes are listed in Table 1 with common functional categories

We then tested the hypothesis that the 22q13.33 insertion was associated with *NHIP* expression in ASD versus TD postmortem brain samples that were matched for age, sex, and race/ethnicity (Additional File 16: Table S15). Similar to the MARBLES cohort of placenta samples, the 22q13.33 insertion showed a significantly higher frequency in ASD compared with TD in a necessarily small group of 58 cortical samples acquired from postmortem brain banks (chi-square test, ASD *n* = 27, TD *n* = 30, *p* value = 0.023; Additional File 3: Fig. S29). RNA-seq was performed on a subset of 20 cortical samples representing all three SV insertion genotypes, matched for age, sex, and race/ethnicity between ASD and TD. Brain samples homozygous for the 22q13.33 insertion (Y) exhibited lower *NHIP* levels compared to those with one or no insertion alleles (N) specifically in ASD, but not in TD samples (Fig. 4a).

We then performed a genome-wide analysis of transcript levels associated with variable *NHIP* transcript levels in brain samples as a continuous response variable. In total, 851 genes passed FDR significance for *NHIP* association, including 195 positively and 656 negatively associated (Fig. 4b, gene list is in Additional File 17: Table S16). After adjustment for sex, age, brain region, and postmortem interval (PMI), 534 genes passed FDR significance for *NHIP* association, of which 445 overlapped with those identified without covariate adjustments, including 166 positively and 368 negatively associated with *NHIP* (Additional File 3: Fig. S30, gene list is in Additional File 18: Table S17). Downregulated genes included ASD candidate genes such as *CHD8* [54], and a gene previously implicated in ASD from placenta, *IRS2* [21] (unadjusted gene list in Additional File 17: Table S16, adjusted gene list in Additional File 18: Table S17). Gene ontology (GO) enrichment analysis of *NHIP*-associated genes (covariate adjusted) revealed 252 terms significant by permutation test (Fig. 4c, FDR-adjusted *p* values in Additional File 19: Table S18). Regulation of nervous system development, including gliogenesis, synaptic vesicle transport, and dendritic spine development, and response to oxidative stress were negatively associated with *NHIP* transcript levels (Fig. 4c, GO term analyses are in Additional File 19: Table S18). GO term functions related to the dendritic spine, synapse assembly, and response to reactive oxygen formed a functional module of genes negatively associated with *NHIP* levels (Additional File 3: Fig. S31, Additional File 19: Table S18). In contrast, transcripts positively associated with *NHIP* levels were enriched for distinct functions in fatty acid metabolism and embryonic organ development. To further examine the relevance of *NHIP* expression to ASD etiology, we overlapped brain *NHIP*-associated transcripts with SFARI ASD risk genes and observed a significant overlap of 65 genes (Fisher’s exact test, *p* value = 1.572E−13, OR = 3.131, Additional File 3: Fig. S32, gene list is in Additional File 20: Table S19). The 65 genes in common were significantly enriched for 14 GO terms, including nervous system development, synapse, chromatin organization, and neurogenesis (Additional File 3: Fig. S33, FDR-adjusted *p* values in Additional File 21: Table S20), demonstrating associations of *NHIP* levels with functionally relevant gene pathways in brain and ASD.

### Overexpression of *NHIP* in HEK293T cells results in large-scale transcriptional changes to genes relevant to brain and ASD risk

To experimentally model the transcriptional impact of *NHIP* induction, RNA-seq and differential expression analyses were performed on HEK293T cells transiently transfected with *NHIP* or vector control. We identified 4756 differentially expressed genes (DEG) with genome-wide significance (FDR-adjusted *p* value < 0.05). *NHIP* overexpression increased expression of 1490 genes and decreased expression of 3266 genes (Additional File 3: Fig. S34, gene list is in Additional File 22: Table S21). Genes decreased with *NHIP* expression included the downstream flanking gene *BRD1*, as well as *IRS2*, *CHD8*, and *DLL1* (Additional File 3: Fig. S35, Additional File 22: Table S21). *NHIP* overexpression and reduced *BRD1* in overexpression cell lines were confirmed with RT-PCR (Additional File 3: Fig. S36). Genes differentially expressed with *NHIP* overexpression were enriched for GO terms associated with noncoding RNA processing, histone modification, placental development, cell cycle, and p53 binding (Additional File 3: Fig. S37, GO term enrichment statistics are in Additional File 23: Table S22), consistent with the proliferation phenotype (Fig. 2f). KEGG gene set enrichment analysis [55] showed enrichment for brain disorders, including Parkinson’s, Alzheimer’s, and Huntington’s diseases and metabolism, such as fatty acid metabolism and drug metabolism (Additional File 3: Fig. S38, KEGG enrichment statistics are in Additional File 24: Table S23), further demonstrating the relevance of *NHIP* regulated genes to brain functions.

In a comparison of in vivo and in vitro RNA-seq analyses, 161 genes overlapped between those differentially expressed in response to experimental *NHIP* overexpression and those associated with *NHIP* transcript levels in human brain, which was significant compared to all expressed genes (> 1 read in any sample, Fisher’s exact test, *p* value = 6.946E-5, OR = 1.482; or > 1 read per sample in both tissues, Fisher’s exact test, *p* value = 8.348E-4, OR = 1.386; Additional File 3: Fig. S39, Additional File 25: Table S24). Genes negatively associated with *NHIP* levels in vitro and in vivo were enriched for functions in synapse, chromatin, and regulation of nervous system development (Additional File 3: Fig. S40, gene list is in Additional File 26: Table S25). Similarly, 20 GO terms overlapped between in vivo and in vitro RNA-seq analyses including regulation of translation, peptide biosynthetic process, and rhythmic process, which was significant compared to all GO terms (Fisher’s exact test, *p* value = 2.24E−5, OR = 0.137, Additional File 27: Table S26). Furthermore, genes differentially expressed with *NHIP* overexpression also showed a significant overlap of 263 genes with ASD risk genes from the SFARI database compared to all expressed genes (Fisher’s exact test, *p* value = 2.752E−05, OR = 1.379) and were enriched for functions in central nervous system development, synaptic signaling, and response to oxygen levels (Additional File 3: Fig. S41-S42, FDR-adjusted *p* values are in Additional Files 28-29: Tables S27-S28). There were 30 genes in common among ASD risk, *NHIP* association in brain, and *NHIP* overexpression, including *BRD4*, *SETD5*, *ARID1B*, *EP300*, and *FOXG1* (Fig. 4d, Table 1, FDR-adjusted *p* values are in Additional File 30: Table S29). Genes common to ASD risk, *NHIP* association in brain, and *NHIP* overexpression were enriched for chromatin organization, regulation of transcription by RNA polymerase II, histone modification, neurogenesis, and rhythmic processes (Table 1, Additional File 3: Fig. S43, GO term enrichment statistics are in Additional File 31: Table S30). Together, these results demonstrate that *NHIP* is a novel regulatory gene with functions relevant to known ASD risk factors.

**Table 1:** Functional categories of genes showing adjusted *NHIP-*associated expression in human cortex, differential expression in *NHIP* overexpressing cells, and known ASD risk (from Fig. 4d overlap)

	GO Terms						
Genes	Chromatin organization	Regulation of transcription by RNA polymerase II	Histone modification	Rhythmic process	Dendritic spine	Regulation of biosynthetic process	Count
*ARID1B*	X	X				X	3
*ASH1L*	X	X	X			X	4
*BRD4*	X	X	X			X	4
*CREBBP*	X	X	X	X		X	5
*EP300*	X	X	X	X		X	5
*HNRNPU*	X	X		X		X	4
*HUWE1*	X		X	X			3
*JMJD1C*	X		X			X	3
*KMT2A*	X	X	X	X		X	5
*PRKCA*	X		X				2
*RERE*	X	X				X	3
*SETD5*	X		X				2
*SMARCA2*	X	X				X	3
*ADNP*		X		X		X	3
*CUX1*		X				X	2
*FOXG1*		X				X	2
*GRIN1*		X			X	X	3
*NR1D1*		X		X	X	X	4
*NR2F1*		X				X	2
*POGZ*		X				X	2
*ZNF292*		X				X	2
*ARHGAP32*					X		1
*CPEB4*					X	X	2
*LRRC4*					X		1
*TNRC6B*						X	1

## Discussion

This study has taken the innovative approach of utilizing placental tissue from a high-risk prospective pregnancy cohort with multi-omic assays to discover a novel ASD risk gene locus that integrates responsiveness to oxidative stress with inheritance of a common structural variant. Given the distinctive DNA methylation landscape of the placenta characterized by PMDs and higher gene body methylation over expressed genes [14, 15, 56], using unbiased WGBS as a tool enabled the discovery of a novel gene associated with ASD that had been missed by standard genetic and epigenetic array-based approaches. The 22q13.33 co-methylated block identified in this study was previously identified by WGBS as a correlated region of increased methylation variance (CoRSIV) [24, 25] as well as a region of increased SV [30] in the human genome. We confirmed the hypothesis that CoRSIV and SV locations overlap more frequently than expected at random (Additional File 3: Fig. S44). Although this 22q13.33 region has not been previously associated with ASD risk, the neighboring distal long arm of 22q13.3 harbors multiple genes implicated in neuropsychiatric disorders, including ASD, intellectual disability, schizophrenia, and bipolar disease [57–59]. *SHANK3*, which encodes a postsynaptic protein required for maturation of glutamatergic synapses [60], is 1.5 Mb telomeric from the 22q13.33 hypomethylated block identified in this study. Rare *SHANK3* mutations are noted in ASD [61], and large structural variations including *SHANK3* are observed in rare ASD children [57]. In addition, 22q13.33, 22q13.32, and 22q13.31 are disease-associated hotspot regions in ASD [33]. While these highly polymorphic regions of the genome have the potential to contain regulatory genes such as *NHIP*, as well as primate-specific sequences relevant to brain development [62], they are often excluded from the design of array-based platforms because of their complexities. The *NHIP* locus is sparsely covered by probes in the most current genetic and epigenetic array designs (Additional File 3: Fig. S14), a likely explanation for why it was not identified by prior ASD studies. In contrast, sequencing-based approaches, such as the integrated WGS and WGBS approach employed here, are a promising alternative for disease association testing.

Placenta is an often misunderstood and overlooked tissue, despite its importance in regulating and thereby reflecting events critical to brain development in utero. Placenta regulates metabolism and provides steroid hormones as well as neurotransmitters critical for the developing brain [63, 64]. Additionally, placenta regulates oxygen supply, as it consumes 40–60% of the body’s oxygen, and hypoxia metabolic adaptation regulates trophoblast cell fate decisions [65, 66]. Oxygen tension can also modulate extravillous trophoblast proliferation, differentiation, and invasion [67], all important for successful implantation and placentation, which can all impact brain development and ASD risk [68–70].

We have demonstrated that *NHIP* is a primate-specific, variably expressed gene responsive to hypoxia in human placenta and brain tissues. The variability in *NHIP* transcript levels was influenced by both non-genetic and genetic factors. First, *NHIP* was induced with neuronal differentiation, but also with hypoxia and oxidative stress. Interestingly, the responsiveness of *NHIP* expression as well as oxidative stress was specific to differentiated neurons but not seen in the undifferentiated state. Oxidative stress is a common convergent mechanism that occurs in normal neurodevelopment but can be excessive in cases of many environmental exposures associated with in ASD, including air pollution [71] and pesticides [72]. Second, prenatal vitamin use in the first month of pregnancy provides essential methyl donors to the one-carbon metabolism pathway [49, 73] that may counteract excessive oxidative stress, a prediction consistent with the elevated methylation over the 22q13.33 block in placentas from pregnancies with first month prenatal vitamin use. Third, common genetic variants were also associated with 22q13.33 methylation levels. While we identified 12 SNPs within the 22q13.33 co-methylated block that were significantly associated with methylation, the strongest genetic factor was a 1.7 kb insertion with a high allele frequency in all ethnicities. Homozygosity for this 22q13.33 insertion was a better predictor of ASD risk than GWAS-based PRS in this mixed ancestry cohort. 22q13.33 SV homozygosity was also strongly associated with hypomethylation of this locus and reduced expression of *NHIP* in ASD compared to TD placenta and brain samples.

Large insertions such as the 22q13.33 SV that occur outside of coding regions can still modify gene expression through alterations in promoter-enhancer loop size. The *NHIP* promoter shows differences in active chromatin marks between individuals and is associated with two CTCF binding sites that apparently anchor an intra-TAD loop between the promoter and a distal fetal brain enhancer. These results suggest a model by which the presence of at least one copy of the reference allele without the insertion would allow *NHIP* to be induced during neurodevelopment and hypoxia, thereby protect the developing brain through its regulation of downstream regulatory gene pathways (Fig. 3f). Homozygosity for the 22q13.33 SV allele is associated with lower *NHIP* expression and less protection, likely because the enhancer-promoter loop forms less efficiently because of the > 15% increased size of the loop. For the minority of TD children who were also homozygous for the 22q13.33 SV, the use of prenatal vitamins that can reduce the consequences of oxidative stress might have been one source of associated protection from risk, although other genetic and environmental factors not investigated may also be involved.

There are several limitations to this study. Because of the relatively small sample size of placentas that came from the only two available ASD-enriched longitudinal studies in the USA (MARBLES and EARLI), the association between 22q13.33 epigenetic and genetic variation with ASD should be replicated in future birth cohorts. Further, the postmortem brain samples used for RNA-seq also constituted relatively small sample sizes, although comparable to other postmortem brain studies in ASD [74–76]. Because the *NHIP*-associated insertion is only detectable in whole genome sequencing studies, not whole exome or SNP arrays, we were limited in performing a larger population analysis of the allele frequencies of the insertion in ASD. In spite of these necessary inherent limitations, this study used an unconventional approach to identify a new gene locus of potential relevance to ASD etiology that will need confirmation by future investigations.

## Conclusions

Much of the human genome is still under-represented in genome- and epigenome-wide association studies with complex diseases such as autism, resulting in an incomplete understanding of heredity, risk, and resilience. Using the novel approach of combining WGBS with WGS in placenta and cord blood samples from two prospective ASD enriched risk cohorts, this study identified a novel regulatory gene *NHIP* expressed in response to hypoxia in placenta and neurons, but modified by a novel structural variant associated with increased ASD risk. These results are expected to be important in understanding gene by environmental interactions in the developing brain and as potential biomarkers of ASD risk at birth, prior to the development of symptoms.

## Methods

### Sample population and diagnostic classification

The Markers of Autism Risk in Babies – Learning Early Signs (MARBLES) study [77] recruited mothers with at least one child that had been diagnosed with ASD and who were pregnant or planning another pregnancy in Northern California, primarily through lists provided by the California Department of Development Services [21, 77–79]. The following criteria were required for MARBLES study’s enrollment: the prospective child has at least one first or second degree relative diagnosed with ASD; the mother is at least 18 years old; the mother is pregnant or planning for a pregnancy; the mother speaks, reads, and understands English proficiently enough in order to complete the protocol; and the mother lives within a 2.5-h drive distance of Davis/Sacramento region. Demographic, diet, and medical information were collected by prospective telephone interviews or questionnaires throughout the pregnancy. For this analysis, a discovery set of 46 placentae from children subsequently diagnosed with ASD and 46 placentae from children subsequently found to have typical neurodevelopment (TD) was sequenced. An internal WGBS replication group included 65 additional MARBLES placenta samples (ASD *n* = 21, Non-TD *n* = 13, TD *n* = 31). Finally, whole genome sequence data were available on 41 ASD and 37 TD MARBLES children, which were used for SNP and SV analyses to characterize WGBS findings.

The Early Autism Risk Longitudinal Investigation (EARLI) study recruited pregnant mothers who already have a child diagnosed with ASD and has been described in detail previously [80]. EARLI families were recruited from four sites (Drexel/Children’s Hospital of Philadelphia, Johns Hopkins/Kennedy Krieger Institute, Kaiser Permanente Northern California, and University of California, Davis) across three US regions (Southeast Pennsylvania, Northeast Maryland, and Northern California). Enrollment criteria for EARLI were as follows: having a biological child diagnosed with ASD; communicating fluently in English or Spanish; being 18 years or older; living within a 2-h drive distance from the study site; and being less than 29 weeks pregnant. For replication analysis of the initial MARBLES WGBS findings, 47 placenta samples (ASD *n* = 16, TD *n* = 31) were available from the EARLI study, with details described previously [81].

In both MARBLES and EARLI studies, the subsequent child diagnosis was clinically assessed by trained, professional examiners at 36 months using standardized instruments including the Autism Diagnostic Observation Schedule (ADOS) [82], Autism Diagnostic Interview – Revised (ADI-R) [83], and Mullen Scales of Early Learning (MSEL) [84]. Based on a previously published algorithm, children were classified into three outcome groups: ASD, TD, and Non-TD [47, 85, 86]. Children with ASD had scores over the ADOS cutoff and fit ASD DSM-5 criteria. Children with TD had all MSEL scores within 2 standard deviations (SD) and no more than one MSEL subscale 1.5 SD below the normative mean together with scores on the ADOS at least three points lower than the ASD cutoff. Children with Non-TD did not meet ASD or TD criteria, but had elevated ADOS scores and low MSEL scores, defined as two or more MSEL subscales with more than 1.5 SD below the normative mean or at least one MSEL subscale more than 2 SD below the normative mean.

### Whole genome bisulfite sequencing (WGBS) library preparation

The placental samples were frozen within 4 h after birth. DNA was extracted from placenta tissue with the Gentra Puregene kit (Qiagen, Hilden, Germany) and quantified with the Qubit DNA Assay Kit (Thermo Fisher Scientific, Waltham, MA, USA). The discovery group included 92 samples (ASD *n* = 46, TD *n* = 46) from the MARBLES study that were used for both WGBS and WGS. For WGBS, DNA was bisulfite converted with the EZ DNA Methylation Lightning kit (Zymo, Irvine, CA, USA). WGBS libraries were prepared from bisulfite-converted DNA using the TruSeq DNA Methylation kit (Illumina, San Diego, CA, USA) with indexed PCR primers and a 14 cycle PCR program. Libraries were sequenced at 2 per lane with 150 bp paired-end reads in Illumina HiSeq X (San Diego, CA, USA) by Novogene (Sacramento, CA, USA). The external replication group included WGBS data from 47 samples (ASD *n* = 16, TD *n* = 31) from the EARLI study, with details described previously [81]. The specificity replication group included 65 samples (ASD *n* = 21, Non-TD *n* = 13, TD *n* = 31) from the MARBLES study. DNA were sonicated to ~ 350 bp using a Covaris E220 (Woburn, MA, USA). Sonicated and size selected DNA was bisulfite converted using the EZ DNA Methylation Lightning kit (Zymo, Irvine, CA, USA). WGBS libraries were prepared using Accel-NGS Methyl-Seq DNA library kit (Swift Biosciences, Ann Arbor, MI, USA) with indexed PCR primers and a 12-cycle PCR program. Libraries were pooled and sequenced on 2 lanes with 150 bp paired-end reads of Illumina NovaSeq 6000 S4 (San Diego, CA, USA) by the DNA Tech Core at University of California, Davis (Davis, CA, USA).

### WGBS alignment and quality control

Raw sequencing files were preprocessed, aligned to the human reference genome, and converted to CpG methylation count matrices with the default parameters in CpG_Me [87–89]. Reads were trimmed to remove adapters and methylation bias on both 5′ and 3′ end. After trimming, reads were aligned to human reference genome hg38 and filtered for PCR duplicates. Cytosine methylation reports were generated using all covered CpG sites. Quality control was examined for each sample and independently between the three sample cohorts because of the different sequencing platforms used. Libraries with CHH methylation greater than 2% were excluded as incomplete bisulfite conversion. The CpG_Me workflow incorporates Trim Galore, Bismark, Bowtie2, SAMtools, and MultiQC [88, 90–93].

### Global methylation and principal component analysis (PCA)

DNA methylation at 20-kb windows sliding across the genome was extracted using the getMeth function in the bsseq R package [94, 95]. Percent methylation for each sample at each window was calculated using the average methylation value from the window. Correlations between DMRs were calculated using Pearson’s correlation coefficient. Principal component analysis (PCA) was performed using the prcomp function in the stats R package and visualized using ggbiplot [96]. The ellipses for each group were illustrated as the 95% confidence limit.

### Methylation array analysis and cell type estimation

The same 92 placenta DNA sample aliquots in the discovery group (ASD *n* = 46, TD *n* = 46) were used for DNA methylation array analysis. DNA was treated and cleaned with the EZ DNA methylation gold kit (Zymo, Irvine, CA, USA). Samples were assayed on the Infinium MethylationEPIC array (Illumina, San Diego, CA, USA) at John Hopkins University CIDR (Baltimore, MD, USA). Raw image files were analyzed using the minfi R package [97]. Data were corrected for background and dye bias with the normal-exponential by out-of-band probe (noob) method [98]. Cell type composition of placenta (trophoblast cells, stromal cells, Hofbauer cells, endothelial cells, and nucleated red blood cells) was estimated from DNA methylation array in the discovery group, WGBS methylation in the external replication and specificity replication group using a sorted placenta cell reference by PlaNET [99].

### Detection of DMRs

DMRs were identified between ASD and TD in the discovery group through DMRichR, with 100 permutations and adjustments for sex and cell types [87, 100]. DMRichR utilized the dmrseq and bsseq algorithms to process methylation levels from a CpG count matrix to identify DMRs [94, 101]. The DMR analysis approach used a smoothing and weighting algorithm that weights CpGs based on coverage. CpGs in physical proximity with similar methylation values were grouped into candidate background regions to estimate region statistics. In permutation testing, DMR percent methylation values were randomly shuffled between diagnosis groups and pooled together to form an approximate null distribution using bsseq [94]. The empirical *p* value was calculated by comparing the observed test to the entire null distribution from all permutation tests to identify significant DMRs. Further correction for genome-wide significance used a FDR of 0.05. Repeating the DMR permutation test with 90 instead of 100 permutations did not change the number of DMRs detected (134 significant by empirical *p* value, 1 after FDR correction) (Additional File 4: Table S3). Individual smoothed methylation levels and chr22q block methylation levels were obtained using the bsseq R package [94]. Genes were assigned to DMRs using the Genomic Regions Enrichment of Annotation Tool (GREAT) tool with the default association settings (5 kb upstream, 1 kb downstream, and 1000 kb max extension) [102]. The distances (kb) were calculated from DMRs to the transcription start site (TSS) of the GREAT assigned genes. Gene Ontology (GO) enrichment analysis for DMRs, hypermethylation DMRs, and hypomethylation DMRs relative to background regions was done using GREAT [102]. Significant terms were called with FDR-corrected *p* values less than 0.05.

### Placenta DMR enrichment analysis

DMRs were examined for enrichment with chromatin marks compared to the background regions using the LOLA R package with Fisher’s exact test followed by FDR correction [103]. Chromatin states were predicted by ChromHMM, which uses a Hidden Markov Model to separate the human genome into 15 functional states based on data from the Roadmap Epigenomics Project [35, 104]. Promoter-related states included active TSS (TssA) (red), TSS flank (TssAFlnk) (orange red), bivalent TSS (TssBiv) (Indian Red), and bivalent TSS flank (BivFlnk) (Dark Salmon) states. Enhancer-related states included genic enhancer (EnhG) (Green Yellow), enhancer (Enh) (Yellow), and bivalent enhancer (EnhBiv) (Dark Khaki). CpG island, shore, shelf, and open sea coordinates were obtained from the annotatr R package [105]. Encyclopedia of DNA Elements (ENCODE) datasets were used to extract histone post-translational modifications (PTMs), including H3K4me1, H3K4me3, H4K9me3, H3K36me3, H3K27me3, and H3K27ac datasets [37, 106]. Enrichment for known transcription factor binding site motif sequences in DMRs was obtained using Hypergeometric Optimization of Motif EnRichment (HOMER) [107].

### Participant whole genome sequencing (WGS) and variant calling

WGS was performed using cord blood DNA from a subset of the same individuals in the discovery group (ASD *n* = 41, TD *n* = 37). Sequencing libraries were generated using the NEBNest DNA library prep kit (NEB, Ipswich, MA, USA) with 150 bp paired-end reads in Illumina HiSeq X (San Diego, CA, USA) by Novogene (Sacramento, CA, USA) with at least 30× coverage per sample. Raw read files were mapped to human reference genome hg38 using Burrows-Wheeler Aligner (BWA) with the default settings [108]. SAMtools was utilized to sort the bam files and Picard was used to merge bam files from the same sample and identify duplicate reads [92, 109]. Single-nucleotide polymorphisms (SNPs), and small insertions and deletions (InDels) were called using GATK and variants were annotated using ANNOVAR [110, 111]. Copy number variations (CNVs) longer than 50 bp were identified using control-FREEC and CREST [112, 113]. Structural variant (SV) detection and genotyping, larger than 50 bp, were performed using DELLY with the default settings [114]. Our criteria included filtering for minor allele frequency (MAF) > 5%. The association of the DMR taken from individual methylation levels using bsseq [94], and variants by linear regression.

### Polygenic risk score (PRS) generation

A subset of individuals from the discovery group were also genotyped using the Illumina Multi-Ethnic genotyping array (ASD *n* = 31, TD *n* = 35). Stringent QC criteria were used on the raw genotypes in order to remove low-quality SNPs and samples [115]. Our criteria included removal of samples with call rates < 98%, sex discrepancy, and relatedness (pi-hat < 0.18) to non-familial samples with filtering out MAF < 5% using PLINK software [116]. After data cleaning, the imputation pipeline was performed using the University of Michigan Imputation Server [117] with minimac4 software [118] to the 1000G Phase v5 reference panel (hg19) [119, 120]. Phasing was performed using Eagle software [121].

PRS calculation was performed on the imputed genetic data, after applying post-imputation filtering (*R*-squared > 0.80). PRS was informed by discovery GWAS results from the combined PGC-iPSYCH genome-wide meta-analysis [8] and generated at a range of *p*_discovery_ thresholds (*p*_discovery_ threshold range from 1 × 10^−8^ to 1.0). Using PLINK software [116], we removed correlated SNPs and applied from 2 to > 20,000 effect sizes to achieve a weighted summation of alleles, representing a PRS for ASD risk. After evaluating via logistic regression the *R*^2^ from a model of ASD on ASD-PRS ranging across the discovery thresholds and adjusting for genetic ancestry, we determined that a *p*_discovery_ of 0.05 achieved the best fit, and thus used this score in further analyses. The association of the 22q13.33 co-methylated block % methylation, taken from individual smoothed chr22q block methylation levels obtained using bsseq [94], and diagnosis with PRS was tested by analysis of variance (ANOVA), with replication by linear regression, with PRS as the dependent variable (Additional File 15: Table S14).

### Participant genomic insertion characterization and Sanger sequencing

To validate the 22q13.33 insertion from Illumina WGS data, the expected genomic location of the insertion was queried in a published PacBio long read sequencing dataset [43]. The insertion was identified as located at the CHM1_chr22-49029645-INS-1673 contig [43]. The contig was in a fasta file with accession number GCA_003709635.1 with the correspondence table, it was also named with GenBank ID QPKN01007947.1 in the NCBI database [44]. SAMtools was utilized to isolate the fasta sequence from the contig (85,271 bp in length) and extract the insertion sequence (1673 bp in length) (Additional File 14: Table S13). The QPKN01007947.1 contig mapped to chr22: 49,381,532–49,466,902 (reference genome: hg19) using blat [122] and the insertion was visualized using Miropeats [123].

In addition to characterizing the insertion using PacBio long read sequencing, primer sets were designed to span the insertion location for PCR-based genotyping (Additional File 14: Table S13). A 25 μl PCR reaction mixture contained 100 ng genomics DNA, 5 μl 5× LongAmp *Taq* reaction buffer (NEB, Ipswich, MA, USA), 1 μl LongAmp *Taq* DNA polymerase (NEB, Ipswich, MA, USA), 1 μl 10 mM dNTPs, and 2 μl of 10 μM forward and reverse primer. The PCR amplifications were performed using following conditions: initial denaturation at 94 °C for 30 s; 30 cycles of denaturing at 94 °C for 30 s, 52 °C for 30 s, and 65 °C for 2 min with a final extension at 65 °C for 10 min. PCR products were subjected to Topoisomerase (TOPO) PCR Cloning Kit (Thermo Fisher Scientific, Waltham, MA, USA) followed by a 1.5% agarose gel electrophoresis with purification and Sanger sequencing by University of California, Davis DNA Sequencing Facility (Davis, CA, USA), and chromatograms were analyzed using SnapGene (Genewiz, South Plainfield, NJ, USA). PCR product genotype and size were characterized using the Bioanalyzer 2100 (Agilent, Santa Clara, CA, USA). The sequence of the insertion was analyzed for repetitive elements using CENSOR and RepeatMasker [124, 125].

### Cell culture, cell-based assays, and transfection

LUHMES cells (ATCC, Manassas, VA, USA, CRL-2927) were seeded on fibronectin-coated plates (Thermo Fisher Scientific, Waltham, MA, USA, CWP001, 354402). Undifferentiated cells were maintained in proliferation medium: Advanced DMEM/F12 (Invitrogen, Carlsbad, CA, USA), supplemented with N2 supplement (Invitrogen, Carlsbad, CA, USA), Penicillin-streptomycin-glutamine (Thermo Fisher Scientific, Waltham, MA, USA), and 40 ng/ml recombinant bFGF (Invitrogen, Carlsbad, CA, USA). To generate differentiated LUHMES, cells were switched to differentiation media for 5 days. Differentiation media comprised advanced DMEM/F12, supplemented with N2 supplement, Penicillin-streptomycin-glutamine, 1 mM dbcAMP (MilliporeSigma, Burlington, MA, USA), 1 μg/ml tetracycline (Neta Scientific, Hainesport, NJ, USA), and 2 ng/ml recombinant human GDNF (Thermo Fisher Scientific, Waltham, MA, USA). For cell viability and hydrogen peroxide production experiments, differentiated cells were grown in 96-well plates for 6 days prior to treatment with CellTiter Blue or ROS-Glo visualization reagent (Promega, Madison, WI, USA). Undifferentiated cells were plated in 96-well plates at the same densities as differentiated neurons and treated identically for cell viability and hydrogen peroxide measurements. For RNA quantification, cells were maintained in 6-well plates. Challenges with hydrogen peroxide (MilliporeSigma, Burlington, MA, USA), cobalt chloride (Thermo Fisher Scientific, Waltham, MA, USA), or mock treatment were carried out after 5 days of differentiation, and cells were treated for 24 h before analysis.

An overexpression *NHIP* plasmid, NHIP-eGFP, was synthesized by VectorBuilder (Chicago, IL, USA) with EF-1α as the promoter for *NHIP* and CMV as the promoter for eGFP fused with a puromycin resistance gene (Additional File 12: Table S11, Additional File 3: Fig. S18). A control plasmid was cut using XbaI and AbaI restriction endonucleases based on NHIP-eGFP, named NEG-eGFP, with *NHIP* removed and the rest of plasmid structure maintained (Additional File 12: Table S11, Additional File 3: Fig. S18). The plasmid for the NHIP peptide, NHIP peptide-eGFP, was synthesized by VectorBuilder with EF-1α as the promoter for the NHIP peptide, and the stop codon removed and fused to the end of the NHIP peptide with eGFP, together with CMV as the promoter for mCherry fused with a puromycin resistance gene (Additional File 12: Table S11, Fig. 2g). All constructs were sequenced with Sanger sequencing by the University of California, Davis, DNA Sequencing Facility (Davis, CA, USA) and analyzed using SnapGene (Genewiz, South Plainfield, NJ, USA) to confirm the expected sequence.

HEK293T cells (ATCC, Manassas, VA, USA, CRL-11268) were grown in DMEM/F12, GlutaMAX medium (Thermo Fisher Scientific, Waltham, MA, USA) supplemented with MEM non-essential amino acids (Thermo Fisher Scientific, Waltham, MA, USA) and 10% fetal bovine serum (Invitrogen, Carlsbad, CA, USA) together with Penicillin-streptomycin-glutamine. Low passage HEK293T cells were transfected with plasmids using Lipofectamine 3000 and Opti-MEM (Invitrogen, Carlsbad, CA, USA) according to the manufacturer’s instructions. Transfections were performed using HEK293T cell lines for each condition. Transfection medium was replaced 24 h post-transfection with complete growth media with puromycin at 3 μg/ml for 7 days.

All cells were maintained at 37 °C containing 95% O_2_ and 5% CO_2_. Images were taken using an EVOS microscope under the magnification labeled in the images. Cell numbers were measured using the Countess II FL automated cell counter (Thermo Fisher Scientific, Waltham, MA, USA) under the default steps with mixing 10 μl of samples with 10 μl of trypan blue. CellTiter Blue reagent was used to measure cell viability using luminescence based on manufacturer instructions (Promega, Madison, WI, USA). H_2_O_2_ production representing relative reactive oxygen species (ROS) level was measured with the ROS-Glo H_2_O_2_ assay system using 50 nM with the default settings with level measured by luminometer (Promega, Madison, WI, USA).

HEK293T whole cell lysates were prepared by resuspension in 1× RIPA buffer and sonication using a Diagenode Bioruptor 300 (Diagenode, Denville, NJ, USA) followed by centrifugation at 21,130×*g* at 4 °C to remove insoluble material and then resolved on a 4–15% SDS-PAGE gel (Biorad, Hercules, CA, USA). The SDS-PAGE gel was rinsed in three changes of water to remove SDS and stained with Imperial protein stain (Thermo Fisher Scientific, Waltham, MA, USA) to visualize proteins. Stained bands between 25 and 37 kDa were carefully excised from the gel, washed in three changes of 50 mM ammonium bicarbonate followed by three washes with acetonitrile, then swollen in 10 mM DTT in acetonitrile and incubated at 56 °C for 30 min to reduce disulfide bonds. The gel pieces were next shrunk by incubation in acetonitrile then incubated in 55 mM iodoacetamide (IAA) in 50 mM ammonium bicarbonate prior to washing with 50 mM ammonium bicarbonate, then shrunk with acetonitrile and dried in a speed vac. Gel pieces were suspended in 50 mM ammonium bicarbonate with 0.01% Protease Max (Promega, Madison, WI, USA) and treated with trypsin (Promega) for 4 h at 50 °C. The NHIP/GFP fusion protein was detected from the resulting peptides by (LC/MS-MS). MS was performed at University of California, Davis Proteomics Core Facility.

NHIP peptide immunofluorescence staining utilized a custom polyclonal antibody that was produced in Rabbit by GenScript Inc (Piscataway, NJ, USA) to a truncated NHIP peptide MVRGEATARTEEAMC and affinity purified. Flash frozen human cortical tissues were fixed in 4% formaldehyde in 1× PBS for 72 h then dehydrated by immersion in 70% ethanol for 7 days and embedded in paraffin. Five-micrometer sections were cut from embedded brain tissue and mounted on glass slides then baked for 4 h at 56 °C. Tissues on slides were washed in four changes of xylene to remove paraffin. Next, slides were washed in two changes of 100% ethanol which was removed by heating to 50 °C on a heat block. The slides were then treated with 1× DAKO antigen retrieval solution (Agilent, Santa Clara, CA, USA) at 95 °C for 1 h in a water bath. Slides were washed five times in 1× PBS with agitation. To reduce endogenous autofluorescence, slides were immersed in 1× PBS and exposed to LED light for 24 h. Slides were next incubated with 1× PBS/0.5% Tween 20/3% BSA for 1 h at 37 °C to block background signals then washed three times in 1× PBS/0.5% Tween 20. Anti-NHIP peptide and control pre-immune antibodies were diluted 1/200 in 1× PBS/0.5% Tween 20/3% BSA and incubated on slides at 37 °C overnight in a humid chamber before three washes in 1× PBS/ 0.5% Tween. Goat anti-Rabbit Alexa 594 (Thermo Fisher Scientific, Waltham, MA, USA, Catalog #A32740) was diluted in 1× PBS/ 0.5% Tween20/ 3% BSA with 5 μg/ml DAPI and added to slides for 2 h at 37 °C in a humid chamber. Slides were washed five times in 1× PBS/ 0.5% Tween 20 with shaking before mounting in 5 μg/ml DAPI in 50% glycerol and application of glass coverslips.

### RNA extraction, cDNA synthesis, and RT-PCR

Total RNA was isolated from HEK293T cells transiently transfected with NHIP-eGFP or negative control NEG-eGFP using the AllPrep DNA/RNA/Protein mini kit (Qiagen, Hilden, Germany). Human tissue total RNA samples were obtained commercially, including placenta (Life Technology, Carlsbad, CA, USA), testes (TaKaRa Bio, Kusatsu, Shiga, Japan), and fetal brain (Cell Applications, San Diego, CA, USA). RNA was extracted from frozen placenta samples in the Discovery group samples using TRIzol Reagent (Invitrogen, Carlsbad, CA, USA). cDNA was synthesized using the High-Capacity cDNA Reverse Transcription Kit (Thermo Fisher Scientific, Waltham, MA, USA) based on the manufacturer’s protocol. TaqMan Gene Expression Assays for *LOC105373085* (renamed as *NHIP*) (assay ID: Hs01034248_s1), *BRD1* (Hs00205849_m1), *FAM19A5* (Hs00395354_m1), and *GAPDH* (assay ID: Hs02786624_g1) were used (Thermo Fisher Scientific, Waltham, MA, USA). The expression of 3 genes of interest and 1 reference genes were examined by real-time TaqMan PCR assay (Thermo Fisher Scientific, Waltham, MA, USA). Expression levels were determined by the probes with optimized primer and probe concentrations. Quantification was accomplished with RT-PCR machine using TaqMan Fast Advanced Master Mix with the default parameters by the manufacturer (Thermo Fisher Scientific, Waltham, MA, USA). Reactions were performed with three biological replicates. Fold changes of transcript levels were measured using the Fluidigm Real-Time PCR Analysis software with fold change of gene expression calculated as the delta delta CT normalized to *GAPDH* (Fluidigm, San Francisco, CA, USA).

### Brain sample acquisition

Human brain samples were obtained from the NICHD Brain and Tissue Bank for Developmental Disorders at the University of Maryland (Baltimore, MD, USA) (Additional File 16: Table S15). RNA from the frozen human brain was purified using AllPrep DNA/RNA/Protein mini kit (Qiagen, Hilden, Germany).

### RNA-seq library preparation and sequencing

RNA from cells and brain was prepared as RNA-seq libraries using Kapa RNA HyperPrep kits (Roche, Basel, Switzerland) together with the QIAseq FastSelect Human ribodepletion kit (Qiagen, Hilden, Germany). Libraries were assessed for quality and quantified on the Agilent Bioanalyzer 2100, and then pooled for multiplex sequencing with at least 25 million reads with 150 bp paired-end reads on the Illumina NovaSeq 6000 S4 (San Diego, CA, USA) by the DNA Tech Core at University of California, Davis (Davis, CA, USA).

### RNA-seq data processing and differential gene expression (DGE)

Raw fastq files were processed and aligned using STAR [126]. After quality control steps by FASTQC, the count matrixes were generated by featureCounts [127, 128]. Count matrixes were filtered for at least one count in any sample. Size factor estimation and normalization were performed by DESeq2 [129]. DGE was generated compared between overexpressed *NHIP* and negative control cells using DESeq2 (FDR-corrected *p* value < 0.05) [129]. DGE for brain was analyzed by using normalized read count for *NHIP* levels as continuous trait using DESeq2 (FDR-corrected *p* value < 0.05) [129]. In brain, the normalized read count for *NHIP* transcripts were adjusted for potential covariates of sex, age, brain region, and PMI using DESeq2 (FDR-corrected *p* value < 0.05). Gene overlaps between different experiments were tested for significance using Fisher’s exact test in the GeneOverlap R package compared to all genes expressed in at least one read in any sample [130].

Gene Ontology terms for DGE were identified using clusterProfiler on Gene Set Enrichment Analysis using gseGO function with 1000 permutations [131]. Normalized enrichment scores (NES) were calculated for enrichment after correcting for multiple testing with FDR. The dotplots illustrate significant GO terms based on GeneRatio, calculated from the number of overlapped genes divided by the total number of genes in the gene set [131]. GO terms to be included in the plots were selected based on GeneRatio ranking. The enrichment map was plotted using emapplot function by clustering mutually overlapping gene sets to form functional modules [131]. The ridgeplot was plotted using the ridgeplot R function to visualize expression distributions of core enriched genes [131]. The cnetplot depicted the linkages of genes and biological concepts as networks [131].

## Supplementary Information

**Additional file 1: Table S1.** Subject characteristics in relation to outcomes at 36 months.

**Additional file 2: Table S2.**. Subject characteristics by subject.

**Additional file 3.** This file contains Figures S1-S44.

**Additional file 4: Table S3.** ASD DMRs identified in discovery group with annotations.

**Additional file 5: Table S4.**. ChromHMM-defined chromatin state enrichment for DMRs.

**Additional file 6: Table S5.** Transcription factor motif enrichment for DMRs.

**Additional file 7: Table S6.** Genes in common between DMRs associated genes and SFARI ASD genes.

**Additional file 8: Table S7.** Gene Ontology terms on ASD DMRs.

**Additional file 9: Table S8.** 22q13.33 comethylated block replicates with independent studies, and sequencing platforms.

**Additional file 10: Table S9.** 22q13.33 comethylated block adjusted for nominal covariates (birth weight, nRBC).

**Additional file 11: Table S10.** Blat results of *NHIP* in vertebrates.

**Additional file 12: Table S11.** Plasmid structure for HEK293T cells and *NHIP* peptide sequence.

**Additional file 13: Table S12.** *NHIP* peptide blat research results.

**Additional file 14: Table S13.** Insertion characteristics in the discovery group and primers.

**Additional file 15: Table S14.** PRS was tested for association on 22q13.33 comethylated block % methylation or diagnosis.

**Additional file 16: Table S15.** Subject characteristics of postmortem brain samples.

**Additional file 17: Table S16.** Differential gene expression associated with *NHIP* in brain.

**Additional file 18: Table S17.** Differential gene expression associated with *NHIP* in brain, after adjustment for covariates.

**Additional file 19: Table S18.** Gene ontology analysis on differentially expressed genes (FDR corrected *p*-value < 0.05) associated with *NHIP* in brain.

**Additional file 20: Table S19.** Genes in common between DGE in brain and SFARI ASD genes.

**Additional file 21: Table S20.** Gene ontology analysis on overlapped genes between DGE in brain and SFARI ASD genes.

**Additional file 22: Table S21.** Differential gene expression with overexpressed *NHIP* in HEK293T cells.

**Additional file 23: Table S22.** Gene ontology analysis of differentially expressed genes (FDR corrected *p*-value < 0.05) related to overexpressed *NHIP* treatment in HEK293T cells.

**Additional file 24: Table S23.** KEGG pathway enrichment of differentially expressed genes (FDR corrected *p*-value < 0.05) with overexpressed *NHIP* treatment in HEK293T cells.

**Additional file 25: Table S24.** Genes in common between DGE in brain and DGE in cells.

**Additional file 26: Table S25.** Gene ontology analysis on the overlapped genes between DGE in brain and DGE in cells.

**Additional file 27: Table S26.** Gene ontology in common between significant gene ontology terms for brain DGE and significant terms for cell DGE.

**Additional file 28: Table S27.** Genes in common between DGE in brain and SFARI ASD genes.

**Additional file 29: Table S28.** Gene ontology analysis on the overlapped genes between DGE in cells and SFARI ASD genes.

**Additional file 30: Table S29.** Genes in common among DGE in brain, DGE in cells, and SFARI ASD genes.

**Additional file 31: Table S30.** Gene ontology analysis on the overlapped genes among DGE in brain, DGE in cells, and SFARI ASD genes.

**Additional file 32.** Review history.

## Data Availability

Datasets supporting the conclusions are available in the Gene Expression Omnibus repository (GEO) [132] at accession number (GSE178206) [133]. Code and scripts for this study are available on GitHub [134]. The gene abbreviation *NHIP* for “neuronal hypoxia inducible, placental associated” for *LOC105373085* was approved by the HUGO Gene Nomenclature Committee.
