# Supplementary material for: Placental methylome reveals a 22q13.33 brain regulatory gene locus associated with autism
Source: Genome Biol. 2022 Feb 16;23:46. doi: 10.1186/s13059-022-02613-1 (PMC8848662; doi:10.1186/s13059-022-02613-1)
Supplement: Supplementary file 3 — Additional file 3. This file contains Figures S1-S44. [file 13059_2022_2613_MOESM3_ESM.pdf]

Supplementary Figures

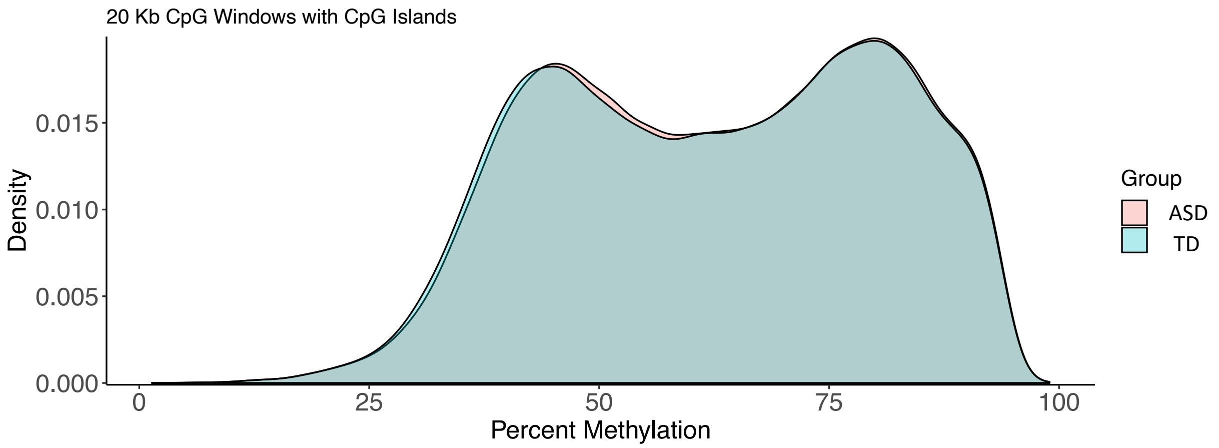

Figure S1. No global methylation difference was observed between ASD and TD comparing methylation levels over 20 kb windows. Results are shown as a density plot of average percent smoothed methylation for CpGs in the discovery group.

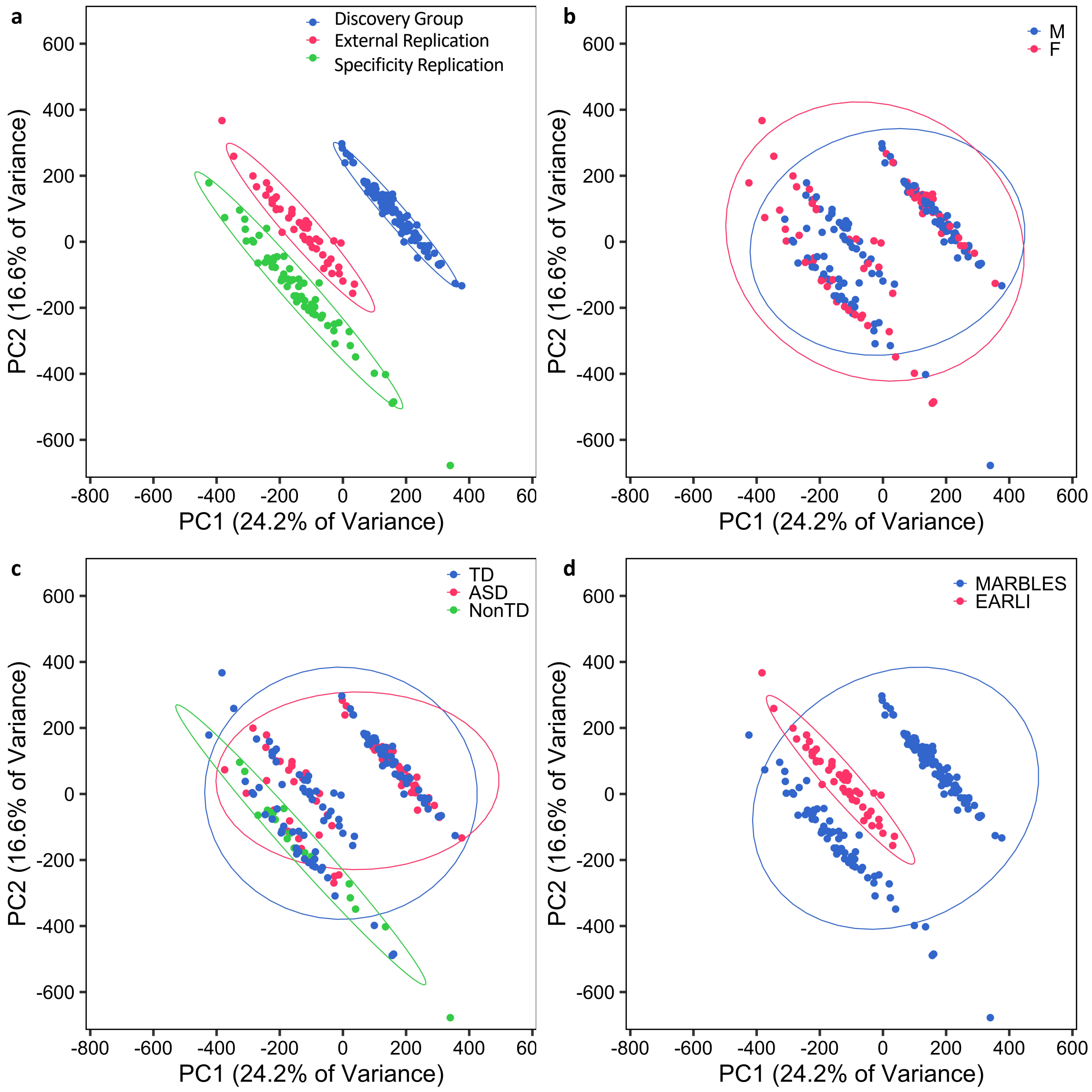

Figure S2. Sequencing platform (HiSeq 4000 vs HiSeq X vs NovaSeq) of each group of samples has a larger effect on methylation levels over 20 kb windows than ASD diagnosis, sex, or study. PCA plots show percent methylation at 20 kb windows across the genome for each sample. Points were colored by group, sex, diagnosis, or study. Ellipses indicate 95% confidence limits. Discovery group: MARBLES, HiSeq X; External Replication group: EARLI, HiSeq 4000; Specificity Replication group: MARBLES, NovaSeq

a

b

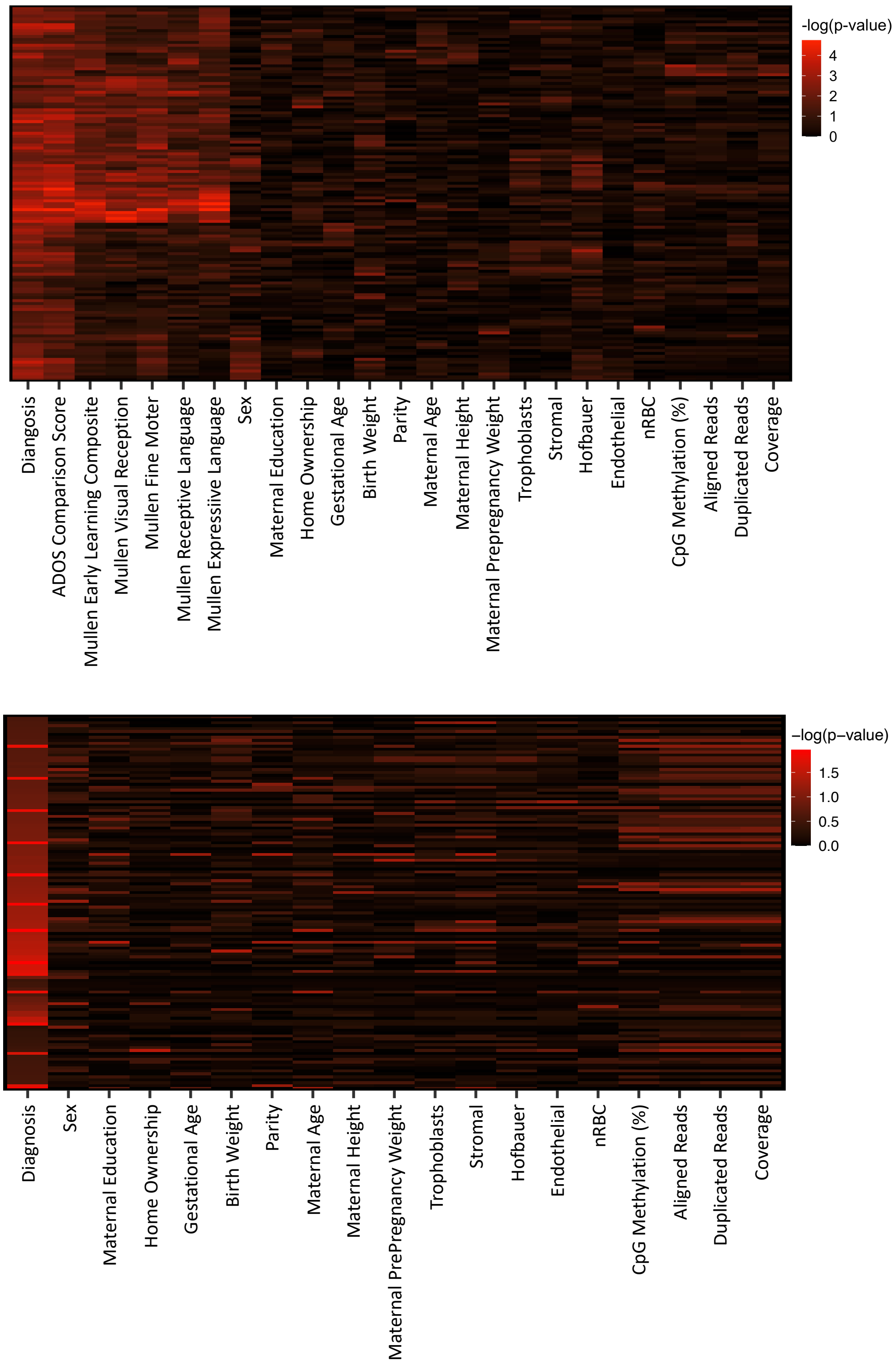
-log(p-value)
4
3
2
1
0
Diangosis
ADOS Comparison Score
Mullen Early Learning Composite
Mullen Visual Reception
Mullen Fine Moter
Mullen Receptive Language
Mullen Expressive Language
Sex
Maternal Education
Home Ownership
Gestational Age
Birth Weight
Parity
Maternal Age
Maternal Height
Maternal Prepregnancy Weight
Trophoblasts
Stromal
Hofbauer
Endothelial
nRBC
CpG Methylation (%)
Aligned Reads
Duplicated Reads
Coverage
–log(p–value)
1.5
1.0
0.5
0.0
Diagnosis
Sex
Maternal Education
Home Ownership
Gestational Age
Birth Weight
Parity
Maternal Age
Maternal Height
Maternal PrePregnancy Weight
Trophoblasts
Stromal
Hofbauer
Endothelial
nRBC
CpG Methylation (%)
Aligned Reads
Duplicated Reads
Coverage

c

	p.val (22q_smooth)
Diagnosis	0.002
Sex	0.572
Material Education	0.303
Home Ownership	0.274
Gestational Age	0.776
Birth Weight	0.126
Parity	0.621
MaternalAge	0.957
Maternal Height	0.394
Maternal Pre-Pregnancy Weight	0.273
Trophoblast cells	0.706
Stromal Cells	0.333
Hofbauer Cells	0.290
Endothelial Cells	0.517
Nucleated Red Blood Cells	0.151
Global CpG Methylation	0.274
Aligned Reads	0.540
Duplicated Reads	0.445
Coverage	0.481

Figure S3. Methylation levels at ASD DMRs and 22q13.33 block are specifically associated with ADOS followed by cognitive behavioral outcomes (Mullen) but independent of other demographic or technical variables.

(**a**) Smoothed methylation values at ASD diagnosis associated DMRs were compared with behavioral, demographic, and technical variables. Significance testing was performed using linear regression.

(**b**) Linear regression models were fitted between smoothed methylation values of each ASD associated DMR with diagnosis, and other potential covariates (sex, maternal education, home ownership, gestational age, birth weight, parity, maternal age, maternal height, maternal pre-pregnancy weight, trophoblast cells, stromal cells, Hofbauer cells, endothelial cells, nucleated red blood cells, global CpG methylation, aligned reads, duplicated reads, and coverage). P values were adjusted for the number of variables within each linear regression model using FDR method.

(**c**) A multivariable linear regression model was fitted between average smooth methylation values over the 22q13.33 hypomethylated block and compared with other covariates (sex, maternal education, home ownership, gestational age, birth weight, parity, maternal age, maternal height, maternal pre-pregnancy weight, trophoblast cells, stromal cells, Hofbauer cells, endothelial cells, nucleated red blood cells, global CpG methylation, aligned reads, duplicated reads, and coverage). (Model: lm(22q_smooth ~ sex + maternal education + home ownership + gestational age + birth weight + parity + maternal age + maternal height + maternal pre-pregnancy weight + trophoblast cells + stromal cells + Hofbauer cells + endothelial cells + nucleated red blood cells + global CpG methylation + aligned reads + duplicated reads + coverage).

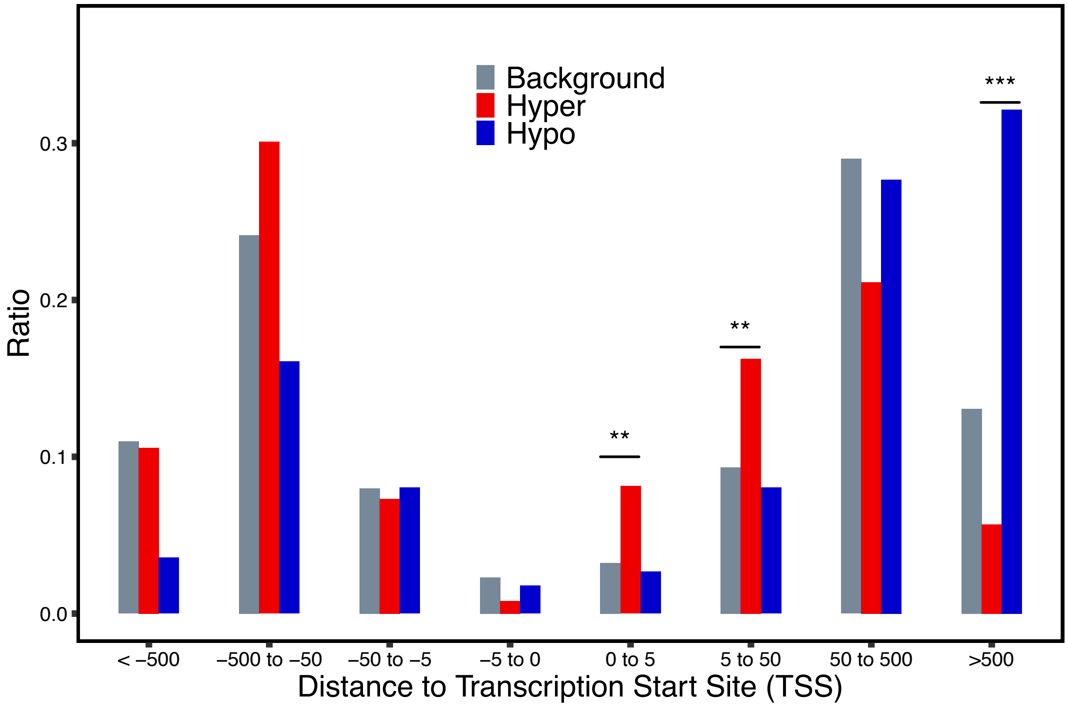

Figure S4. ASD associated DMRs were enriched at 5 kb and 5 – 50 kb downstream of TSS's compared to background regions.

Locations relative to genes for hypermethylated (red) and hypomethylated (blue) ASD DMRs compared to background regions (grey). Distributions of locations relative to transcription start sites (TSS). The ratio was plotted based on the number of genes at each binned location divided by the total number of genes. Significance levels were calculated using Fisher's exact test. (* *p*-value < 0.05, ** *p*-value < 0.01, *** *p*-value < 0.001).

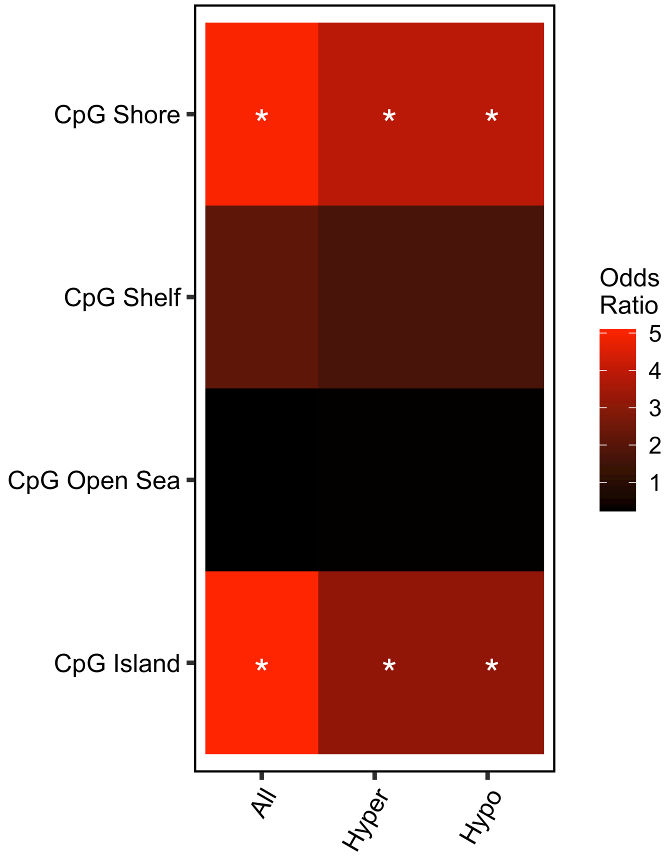

Figure S5. ASD DMRs were enriched at CpG islands and CpG shores.
ASD DMRs (categorized as all, hypermethylated, or hypomethylated in ASD) were tested for enrichment based on CpG island location. Asterisks (*) indicate significant enrichment compared to background regions using the LOLA R package with Fisher's exact test after FDR correction.

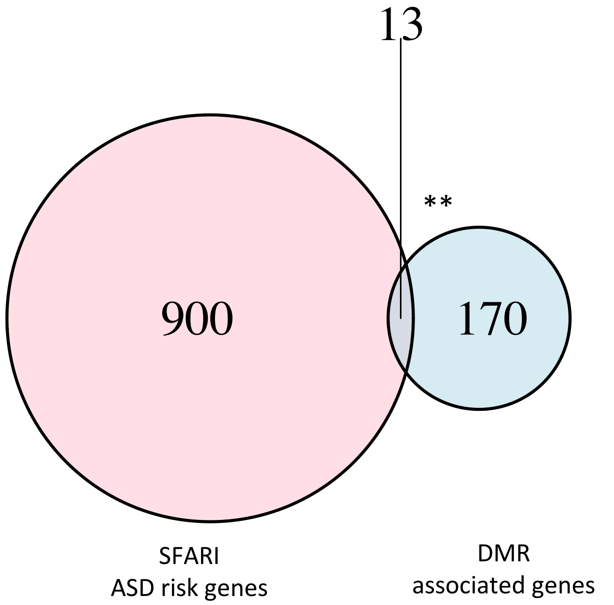

Figure S6. ASD DMR associated genes significantly overlapped with SFARI ASD genes (Fisher's exact test, *p*-value < 0.01). Gene overlaps were tested for significance using Fisher's exact test in the GeneOverlap R package compared to background regions.

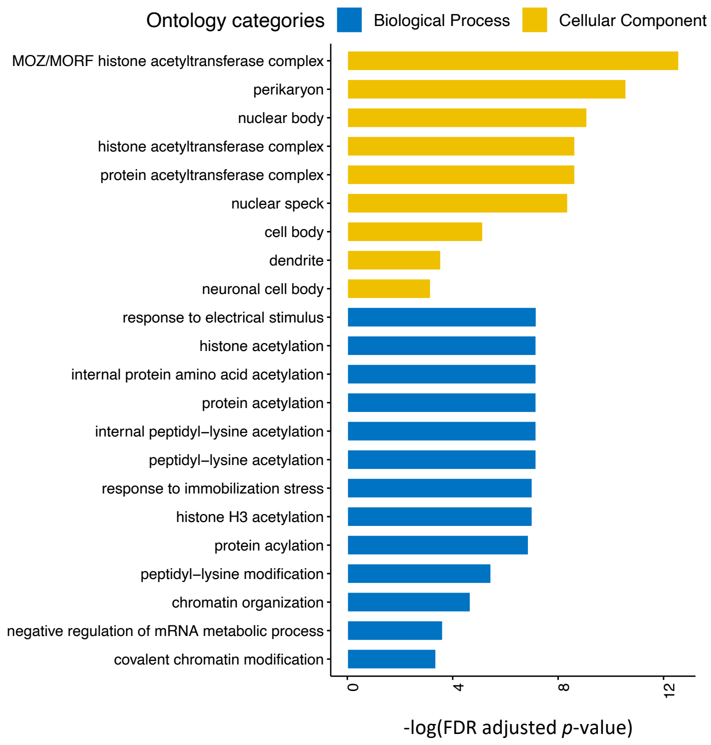

Figure S7. ASD DMRs were enriched for genes with histone acetyltransferase and chromatin modification functions.

Bar graph represents GREAT gene ontology and pathway enrichment analysis of ASD DMR annotated genes compared to background region annotated genes using Fisher's exact test (FDR adjusted *p*-value).

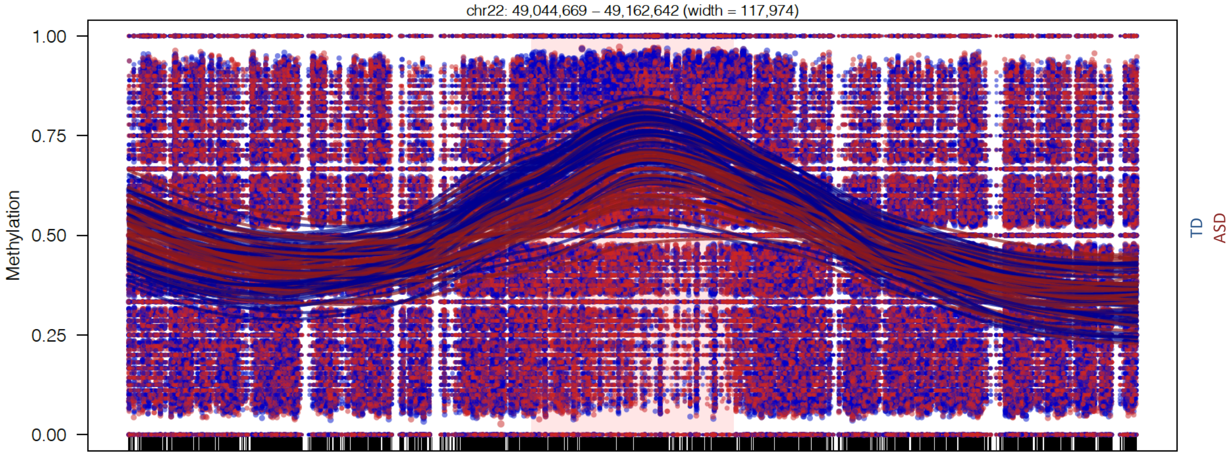

Figure S8. 22q13.33 co-methylated block identified as hypomethylated in ASD.
The lines represent individual smoothed methylation levels estimated for ASD (red) or TD (blue). The dots represent the methylation level estimated at individual CpGs for each sample.

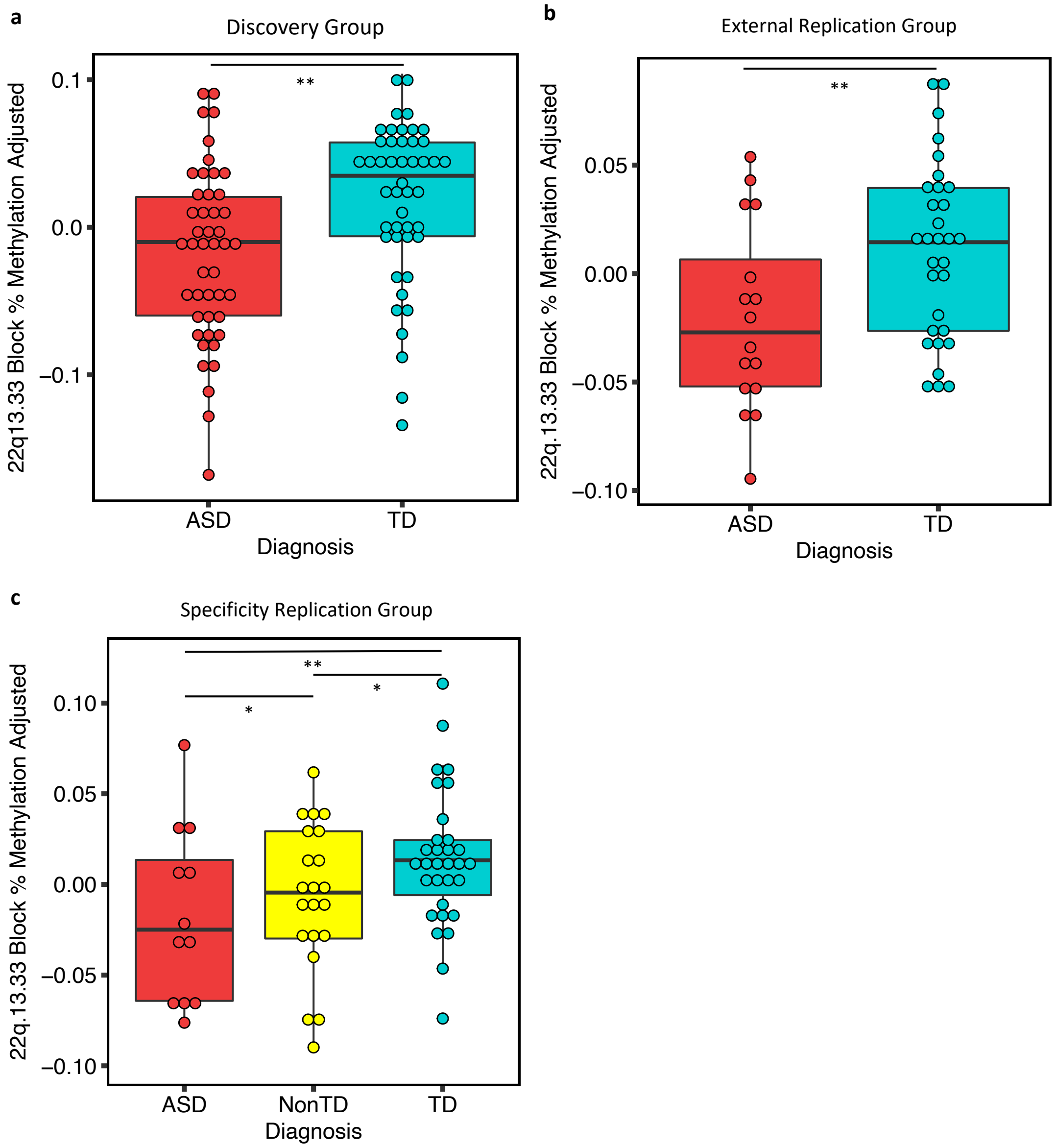

Figure S9. Smoothed methylation values (y-axis) were averaged over the 22q13.33 block with adjustment for potential covariates, birthweight and nucleated red blood cells (nRBC), and compared across diagnosis group (x-axis).

(**a**) ASD samples had significantly lower methylation compared to TD samples in the discovery group (MARBLES, HiSeq X, ASD n = 46, TD n = 46) (*p*-value = 0.003).

(**b**) In the external replication group, ASD samples also had significantly lower methylation than TD group (EARLI, HiSeq 2500, ASD n = 16, TD n = 31) (*p*-value = 0.0098).

(**c**) For the specificity replication group (MARBLES, NovaSeq, ASD n = 21, Non-TD n = 13, TD n = 31), ASD methylation levels were also significantly lower than both TD (*p*-value = 0.004) and Non-TD (*p*-value = 0.0496), while Non-TD was significantly lower than TD samples (*p*-value = 0.0494).

Box plot center lines, box limits, and whiskers represented median, interquartiles range, and minimum and maximum values.

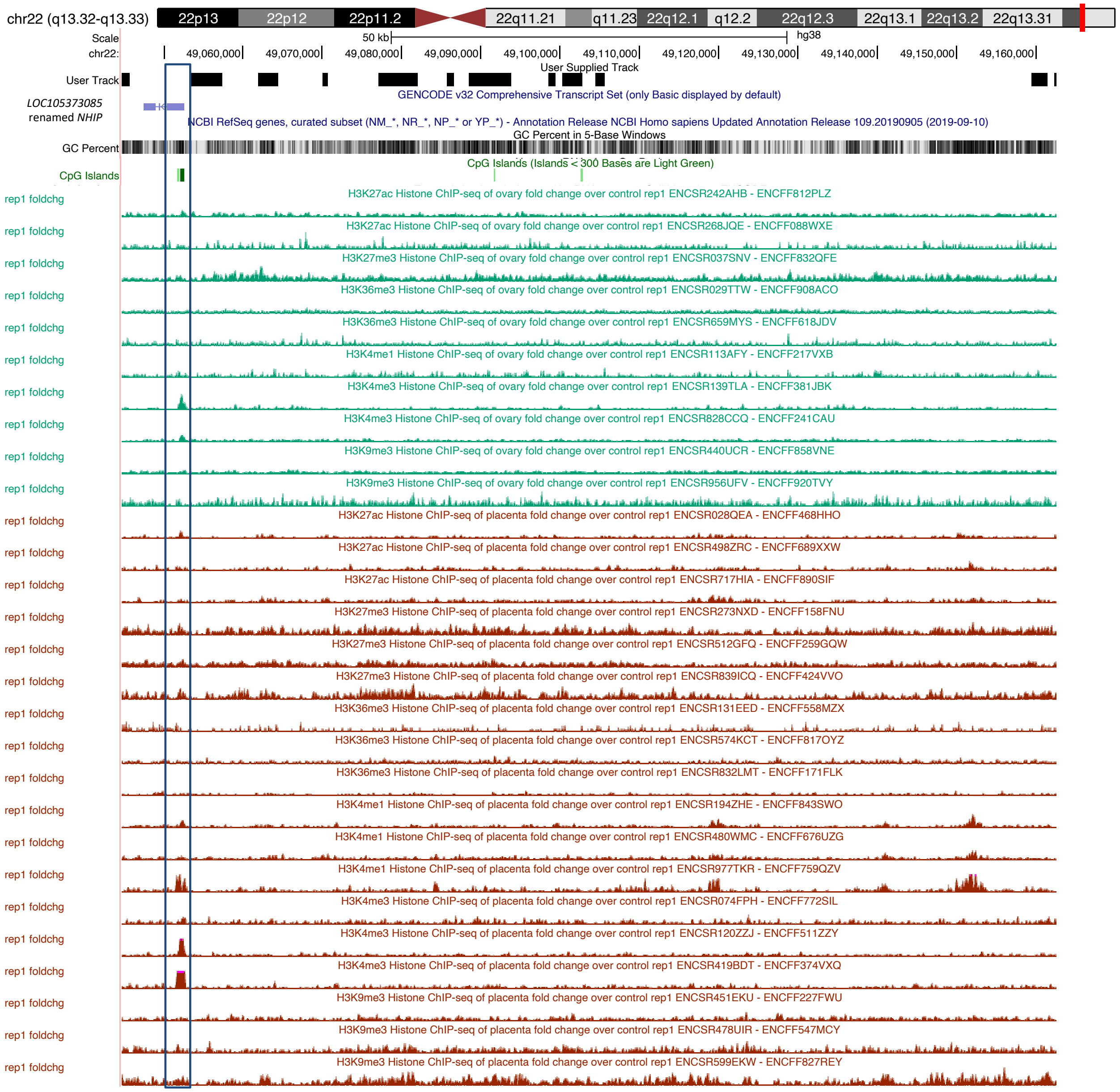

Figure S10. H3K4me3 peaks were observed at 22q13.33 co-methylated block.
Histone post-translational modifications (PTMs) markers for ovary and placenta were extracted from ENCODE data [1,2] at the 22q13.33 co-methylated block region using the UCSC genome browser. Black box illustrates the H3K4me3 peak observed on the CpG island at the TSS of *NHIP*.

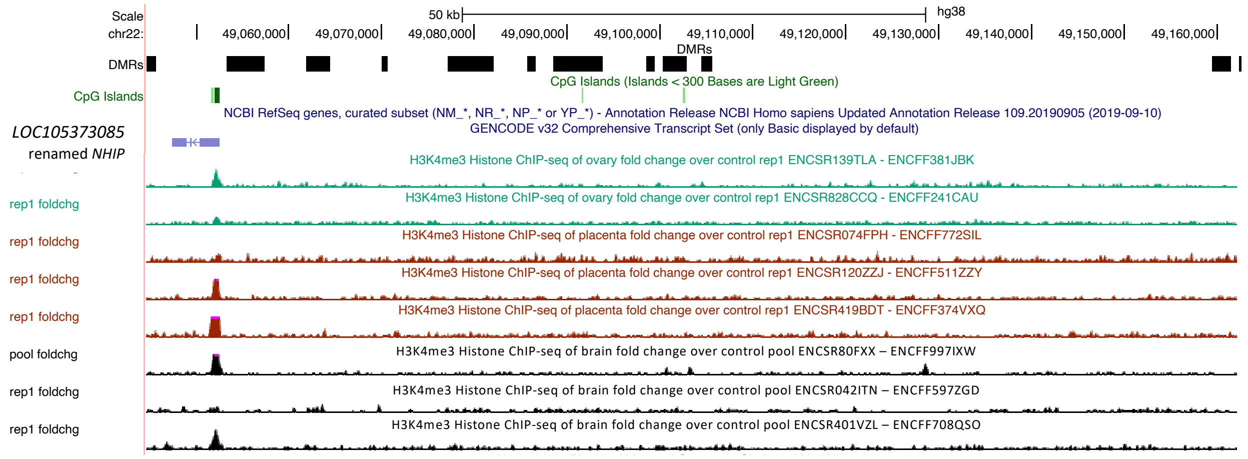

Figure S11. The H3K4me3 peak at the TSS of *NHIP* appears to be polymorphic among individuals in ovary, placenta, and brain.

H3K4me3 peaks from multiple individuals for ovary, placenta, and brain were extracted from ENCODE data [106] at the 22q13.33 co-methylated block region using the UCSC genome browser. There was variability in the existence of the H3K4me3 peak among individuals.

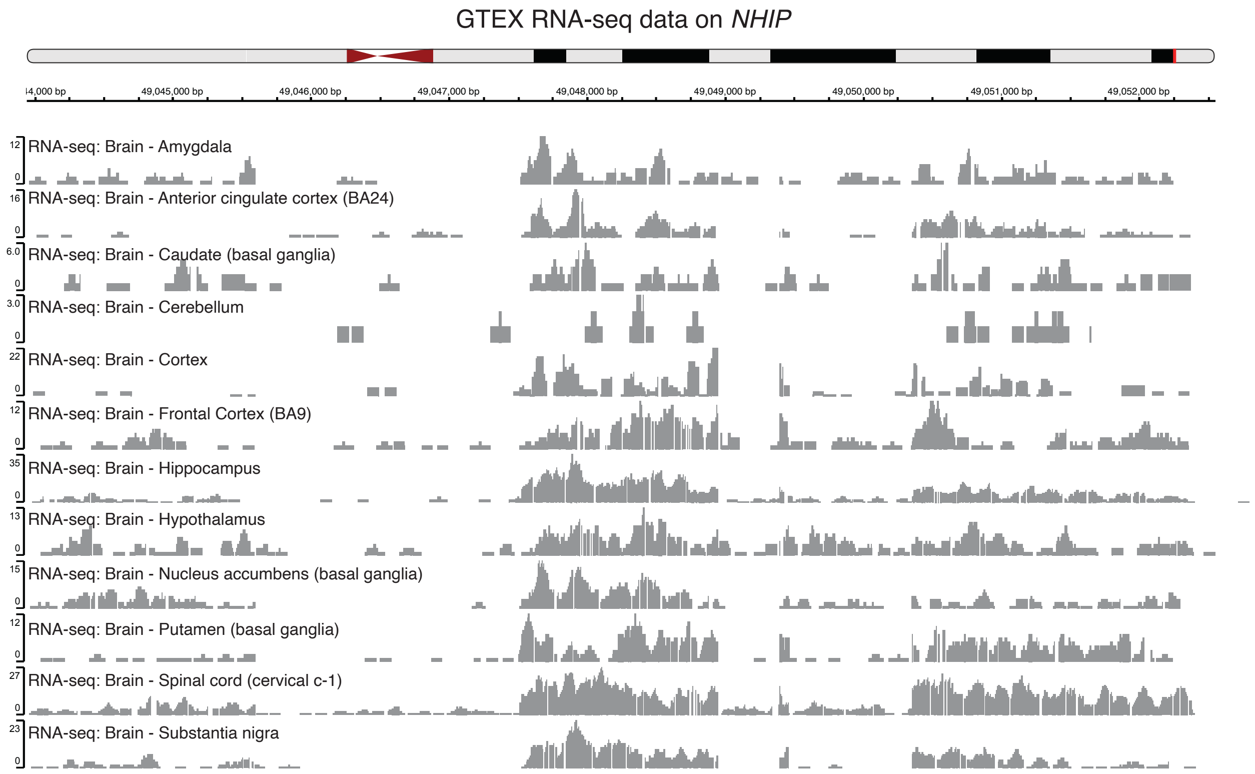

Figure S12. *NHIP* was variably expressed between different brain regions. *NHIP* location was queried in various brain regions using the Genotype-Tissue Expression (GTEx) database [24, 39]. Various brain regions show polymorphic expression levels of *NHIP,* with relatively high expression in cortex and low expression in cerebellum.

Species	Query start	Query end	Length	Score	E-val	%ID	Species
Human (Homo sapiens)	1	8609	8609	17016	0	100	Primates
Gorilla (Gorilla gorilla gorilla)	1	8609	8672	13460	0	97.16	Primates
Bonobo (Pan paniscus)	1	8609	8669	12011	0	97.87	Primates
Chimpanzee (Pan troglodytes)	1	8609	8677	11964	0	97.78	Primates
Orangutan (Pongo abelii)	1	8609	8638	7979	0	94.55	Primates
Gibbon (Nomascus leucogenys)	40	8609	7663	7232	0	94.39	Primates
Crab-eating macaque (Macaca fascicularis)	46	8609	8570	6327	0	90.15	Primates
Gelada (Theropithecus gelada)	46	8609	8609	6327	0	89.98	Primates
Olive baboon (Papio anubis)	46	8609	8563	6275	0	90.02	Primates
Pig-tailed macaque (Macaca nemestrina)	46	8609	8609	6260	0	89.98	Primates
Macaque (Macaca mulatta)	46	8077	8567	6204	0	89.79	Primates
Drill (Mandrillus leucophaeus)	46	8077	7979	6147	0	89.74	Primates
Black snub-nosed monkey (Rhinopithecus bieti)	40	7499	9243	6078	0	89.36	Primates
Angola colobus (Colobus angolensis palliatus)	40	8601	8631	6060	0	89.33	Primates
Sooty mangabey (Cercocebus atys)	46	8609	8580	6032	0	89.3	Primates
Golden snub-nosed monkey (Rhinopithecus roxellana)	40	8609	9014	6024	0	89.23	Primates
Vervet-AGM (Chlorocebus sabaeus)	40	8609	8601	5340	0	90.66	Primates
Ugandan red Colobus (Piliocolobus tephrosceles)	980	8609	7628	5178	0	90.19	Primates
Ma's night monkey (Aotus nancymaae)	977	7176	7351	2170	0	84.83	Primates
Bolivian squirrel monkey (Saimiri boliviensis boliviensis)	3697	7499	3576	1391	0	86.14	Primates
Capuchin (Cebus capucinus imitator)	46	7176	6416	1077	0	84.13	Primates
Marmoset (Callithrix jacchus)	980	1808	852	557	3.00E-155	83.45	Primates
Tarsier (Carlito syrichta)	1832	6760	1596	557	3.00E-155	83.9	Primates
American bison (Bison bison bison)	7250	7508	259	251	5.00E-63	87.26	Ungulates
Greater bamboo lemur (Prolemur simus)	7237	7476	241	229	1.00E-56	87.14	Primates
American black bear (Ursus americanus)	1894	2072	179	204	8.00E-49	89.39	Carnivores
Polar bear (Ursus maritimus)	1894	2072	179	204	7.00E-49	89.39	Carnivores
Alpaca (Vicugna pacos)	7252	7464	214	192	3.00E-45	86.45	Ungulates
Panda (Ailuropoda melanoleuca)	1894	2072	179	188	4.00E-44	88.27	Carnivores
Agassiz's desert tortoise (Gopherus agassizii)	7250	7491	243	186	2.00E-43	84.77	Birds and Re
Coquerel's sifaka (Propithecus coquereli)	7247	7463	217	184	8.00E-43	85.71	Primates
Cat (Felis catus)	7252	7508	265	178	4.00E-41	84.53	Carnivores
Donkey (Equus asinus asinus)	1862	2058	197	166	1.00E-37	85.79	Ungulates
Horse (Equus caballus)	1862	2058	198	164	6.00E-37	85.86	Ungulates
Common canary (Serinus canaria)	7251	7414	164	164	3.00E-37	87.8	Birds and Re
Goat (Capra hircus)	1906	2034	129	160	1.00E-35	90.7	Ungulates
Rat (Rattus norvegicus)	1899	2038	140	158	4.00E-35	89.29	Rats & Mice

Figure S13. Full length *NHIP* was identified in primates, but not other mammals by blat search. *NHIP* DNA sequence was extracted to blat search compared against vertebrate databases [122].

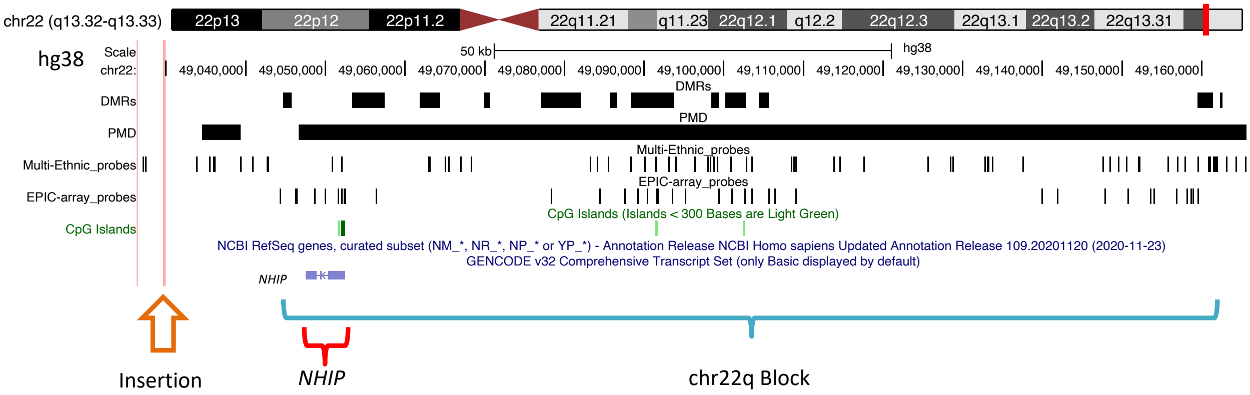

Figure S14. 22q13.33 hypomethylated block location relative to PMDs, multi-ethnic SNP probes, and EPIC-array probe locations.

UCSC genome browser (genome assembly: hg38) with the 22q13.33 hypomethylated block (blue), the insertion location (orange), and *NHIP* (red). 12 DMRs located inside the 22q13.33 hypomethylated block are shown in the DMR track. PMD locations are illustrated in the PMD track [15]. SNP-array probe location was extracted from Illumina Infinium multi-ethnic genotyping array. EPIC-array probe location was extracted from Illumina Infinium MethylationEPIC.

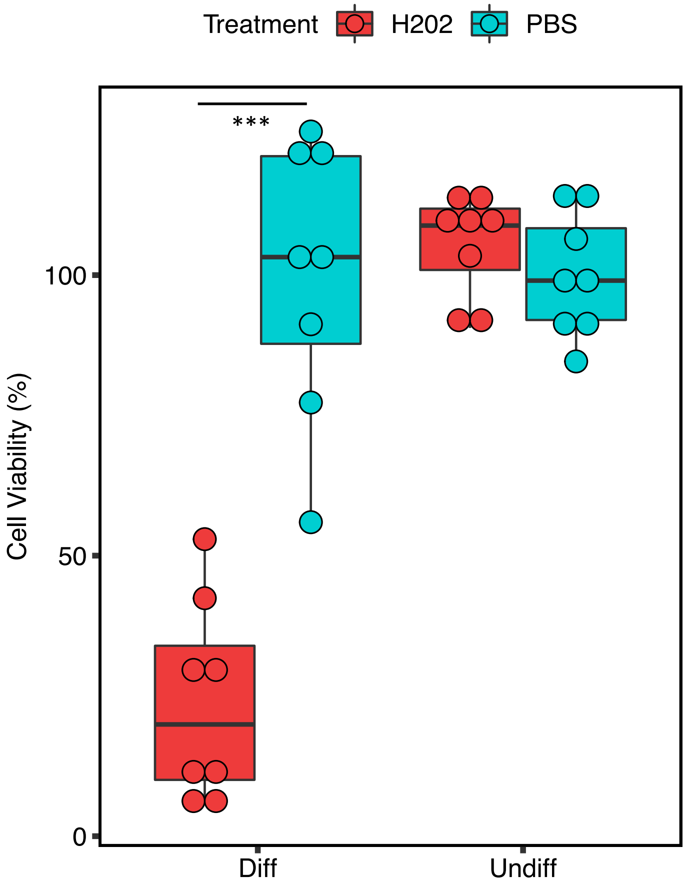

Figure S15. Differentiated LUHMES cells are more sensitive to oxidative stress than undifferentiated LUHMES cells.
Cell viabilities were detected by fluorescent intensity of CellTiter luminescent assay. The relative levels in treated cells were generated by comparing with the average of PBS-treated cells for both differentiated and undifferentiated experiments (Mann-Whitney-Wilcoxon, *p*-value < 0.0001, differentiated LUHMES treated with 50nM H_20_2 (hypoxia mimetic) n = 8, differentiated LUHMES treated with vehicle (mock) n = 8, effect size = -3.58).

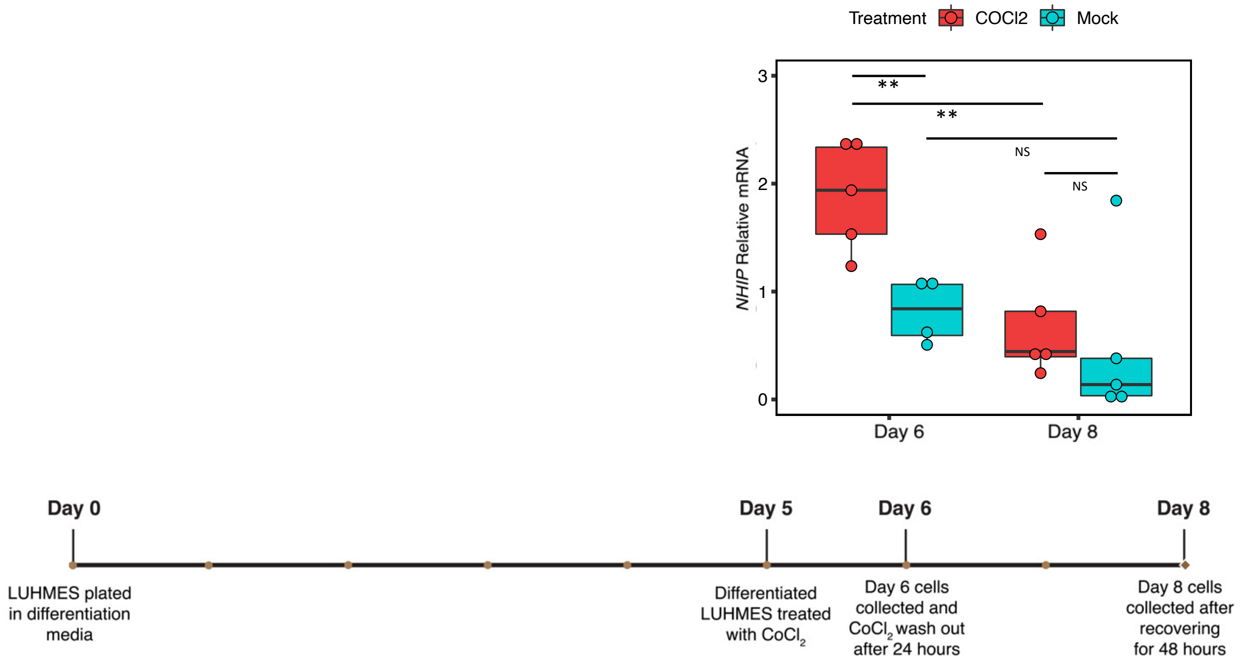

Figure S16. *NHIP* transcript levels return to baseline two days following removal of hypoxia mimetic.

LUHMES cells were plated in differentiation media at Day 0. At Day 5, differentiated LUHMES cells were treated with $CoCl_2$. After 24 hours of $CoCl_2$ treatment (Day 6), LUHMES cells treated with $CoCl_2$ showed significantly increased *NHIP* transcript level compared with LUHMES cells with mock treatment (Mann-Whitney-Wilcoxon, *p*-value = 0.009, differentiated LUHMES treated with 100nM $CoCl_2$ (hypoxia mimetic) n = 5, differentiated LUHMES treated with vehicle (mock) n = 4, effect size = 2.58). Cells were washed and replaced with media at Day 6 to allow LUHMES cells to recover. *NHIP* transcript level returned to mock control baseline levels after 48 hours of recovery at Day 8 (treatment *p*-value = 0.223). *NHIP* transcript level remained unchanged between Day 6 and Day 8 in the mock treatment cells (Mann-Whitney-Wilcoxon, *p*-value = 0.2000), while differentiated LUHMES treated with $CoCl_2$ at Day 6 had a significantly higher level of *NHIP* compared with 48 hours after $CoCl_2$ removal on Day 8 (Mann-Whitney-Wilcoxon, *p*-value = 0.009).

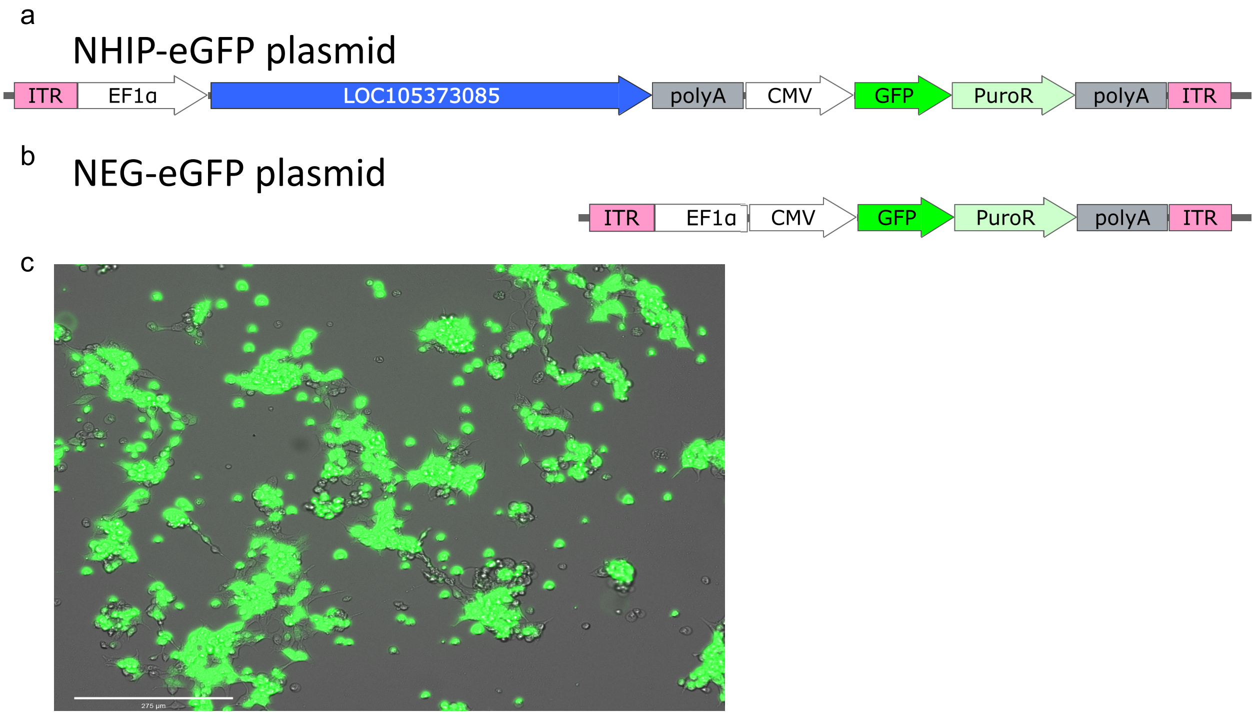

Figure S17. HEK293T cells were successfully transfected with overexpressed *NHIP*. Schematic diagram of vector used for HEK293T cell transfection.

(**a**) Plasmid with *NHIP*, pb-NHIP-eGFP. EF-1α as promoter for *NHIP* and CMB as promoter for eGFP fused with puromycin resistant gene.

(**b**) Negative control plasmid without *NHIP*, pb-NEG-eGFP. Remove *NHIP* from pb-NHIP-eGFP and remove the rest of plasmid structure.

(**c**) Successful transfection image on HEK293T cell using EVOS microscopy.

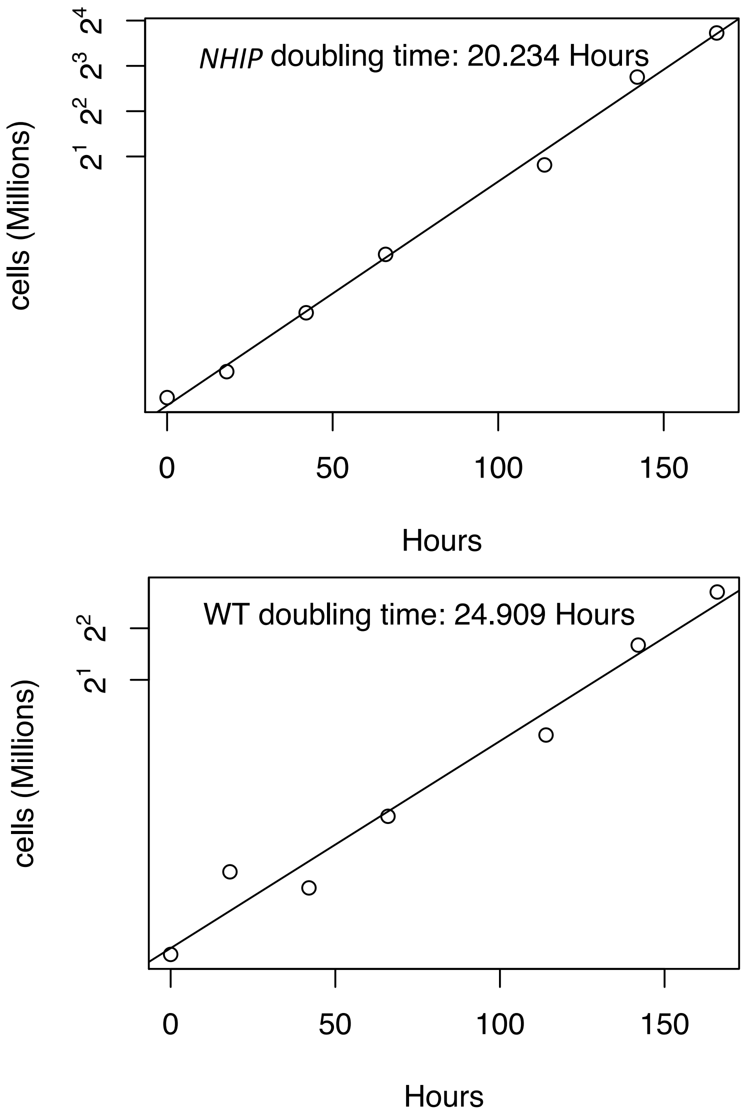

Figure S18. HEK293T cells overexpressing *NHIP* exhibited a significantly altered cell cycle. Overexpression of *NHIP* in HEK293T cells resulted in a significantly shortened cell cycle doubling time (overexpression cells doubling time = 20.23 h, wild type cells doubling time = 24.91 h). Doubling times were calculated based on the 1/slope of the regression between log (cells) and time.

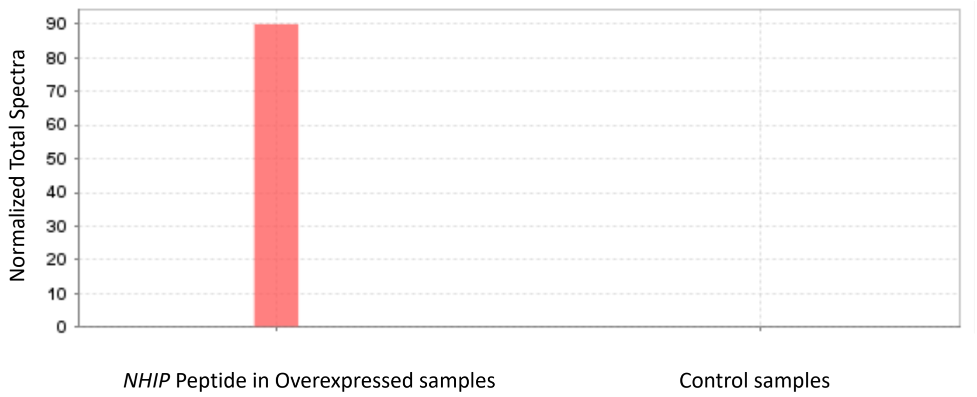

Figure S19. Mass spectrometry of NHIP peptide and negative control.

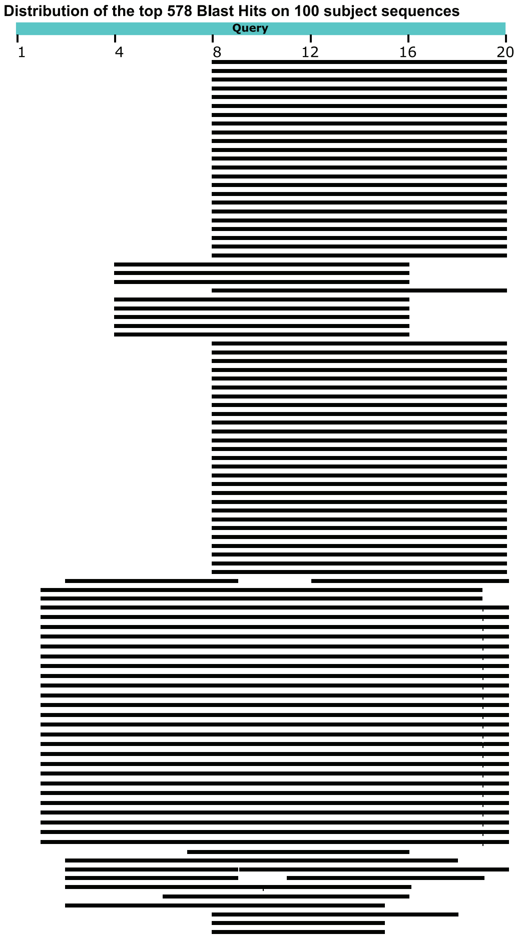

BLAST ® » **blastp suite** » results for RID-DBJE4WD201R

Your search parameters were adjusted to search for a short input sequence.
Your search is limited to records that include: Home sapiens (taxid:9606)

Job Title	Protein Sequence ...
RID	DBJE4WD201R Search expires on 06-03 11:38 am
Program	Quick BLASTP
Database	nr
Query ID	lcl|Query_38615
Description	None ...
Molecule type	amino acid
Query Length	20

Descriptions

Description	Max Score	Total Score	Query Cover	E value	Per. Ident	Accession
truncated breast and ovarian cancer susceptibility protein 2 [Homo sapiens]	26.9	26.9	60%	3.7	66.67%	AYD59793.1
truncated breast and ovarian cancer susceptibility protein 2 [Homo sapiens]	26.9	26.9	60%	3.8	66.67%	AYD59803.1
DNA repair-associated BRCA2 [Homo sapiens]	26.9	26.9	60%	3.8	66.67%	QCL11117.1
truncated breast and ovarian cancer susceptibility protein 2 [Homo sapiens]	26.9	26.9	60%	3.8	66.67%	AYD59769.1
truncated breast and ovarian cancer susceptibility protein 2 [Homo sapiens]	26.9	26.9	60%	3.8	66.67%	AYD59770.1
truncated breast and ovarian cancer susceptibility protein 2 [Homo sapiens]	26.9	26.9	60%	3.8	66.67%	AYD59771.1
truncated breast and ovarian cancer susceptibility protein 2 [Homo sapiens]	26.9	26.9	60%	3.8	66.67%	AYD59772.1
truncated breast and ovarian cancer susceptibility protein 2 [Homo sapiens]	26.9	26.9	60%	3.8	66.67%	AYD59773.1
truncated breast and ovarian cancer susceptibility protein 2 [Homo sapiens]	26.9	26.9	60%	3.8	66.67%	QGJ03648.1
truncated breast and ovarian cancer susceptibility protein 2 [Homo sapiens]	26.9	26.9	60%	3.8	66.67%	AYD59774.1
truncated breast and ovarian cancer susceptibility protein 2 [Homo sapiens]	26.9	26.9	60%	3.8	66.67%	AYD59775.1
truncated breast and ovarian cancer susceptibility protein 2 [Homo sapiens]	26.9	26.9	60%	3.8	66.67%	AYD59776.1
truncated breast and ovarian cancer susceptibility protein 2 [Homo sapiens]	26.9	26.9	60%	3.8	66.67%	QGJ03647.1
truncated breast and ovarian cancer susceptibility protein 2 [Homo sapiens]	26.9	26.9	60%	3.8	66.67%	AYD59777.1
truncated breast and ovarian cancer susceptibility protein 2 [Homo sapiens]	26.9	26.9	60%	3.8	66.67%	AYD59778.1
truncated BRCA2 DNA repair-associated protein [Homo sapiens]	26.9	26.9	60%	3.8	66.67%	QIM56776.1
truncated breast and ovarian cancer susceptibility protein 2 [Homo sapiens]	26.9	26.9	60%	3.8	66.67%	AYD59779.1
truncated breast and ovarian cancer susceptibility protein 2 [Homo sapiens]	26.9	26.9	60%	3.8	66.67%	AYD59780.1
truncated breast and ovarian cancer susceptibility protein 2 [Homo sapiens]	26.9	26.9	60%	3.8	66.67%	AYD59781.1
truncated breast and ovarian cancer susceptibility protein 2 [Homo sapiens]	26.9	26.9	60%	3.8	66.67%	AYD59782.1
DNA repair-associated BRCA2 [Homo sapiens]	26.9	26.9	60%	3.8	66.67%	QCL11116.1
truncated breast and ovarian cancer susceptibility protein 2 [Homo sapiens]	26.9	26.9	60%	3.8	66.67%	AYD59784.1
truncated breast and ovarian cancer susceptibility protein 2 [Homo sapiens]	26.9	26.9	60%	3.8	66.67%	QGJ03646.1
unnamed protein product [Homo sapiens]	26.9	26.9	60%	3.8	66.67%	BAG54725.1
chromodomain-helicase-DNA-binding protein 4 isoform 3 [Homo sapiens]	26.9	41.1	60%	3.8	66.67%	NP_001350535.1
chromodomain-helicase-DNA-binding protein 4 isoform 2 [Homo sapiens]	26.9	41.1	60%	3.8	66.67%	NP_001284482.1
truncated breast and ovarian cancer susceptibility protein 2 [Homo sapiens]	26.9	26.9	60%	3.8	66.67%	AYD59785.1
chromodomain helicase DNA binding protein 4, isoform CRA_c [Homo sapiens]	26.9	41.1	60%	3.8	66.67%	EAW88779.1
chromodomain helicase DNA binding protein 4, isoform CRA_a [Homo sapiens]	26.9	41.1	60%	3.8	66.67%	EAW88777.1
chromodomain helicase DNA binding protein 4, isoform CRA_b [Homo sapiens]	26.9	41.1	60%	3.8	66.67%	EAW88778.1
chromodomain-helicase-DNA-binding protein 4 isoform 1 [Homo sapiens]	26.9	41.1	60%	3.8	66.67%	NP_001264.2
CHD4 protein [Homo sapiens]	26.9	41.1	60%	3.8	66.67%	AAH38596.1

Figure S20. NHIP peptide in blat search [122] with hits on BRCA2 and CHD4.

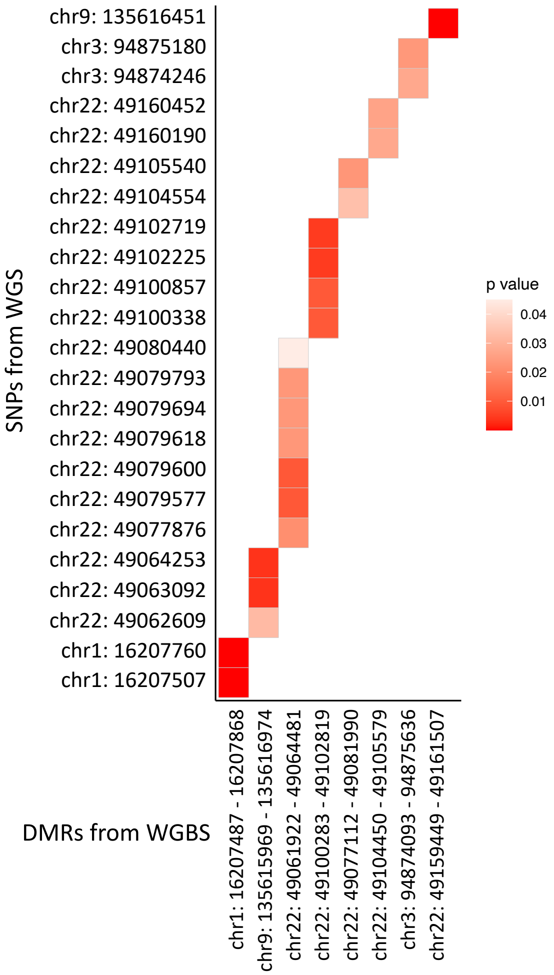

Figure S21. Percent methylation at eight DMRs was significantly associated with *in cis* SNPs. Percent methylation for each DMR was extracted from WGBS as average smooth methylation of the DMR region. WGS data was used to extract the *in cis* SNPs located within the DMRs. Significance tests were done between the *in cis* SNPs and DMRs using linear regression, with *p*-value < 0.05 shown (discovery group, ASD n = 41, TD n = 37).

Diagnosis \ Insertion	Yes	No
ASD	20	21
TD	9	27

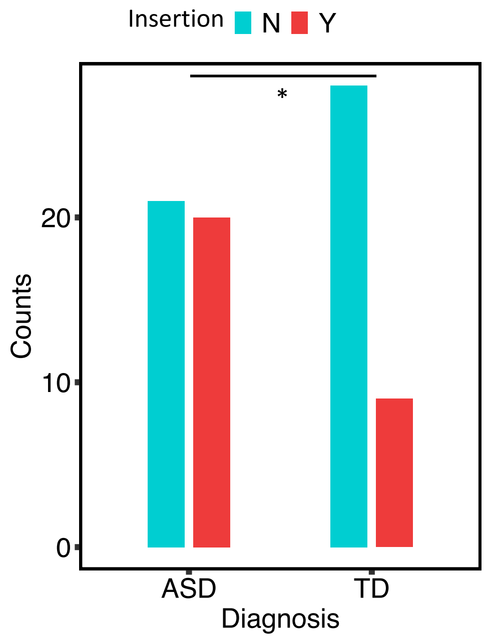

Figure S22. Count matrix of the 1.7 kb insertion genotypes, with a significantly higher frequency in ASD compared to TD (discovery group, ASD n = 41, TD n = 37) (chi-square test, *p*-value = 0.045).

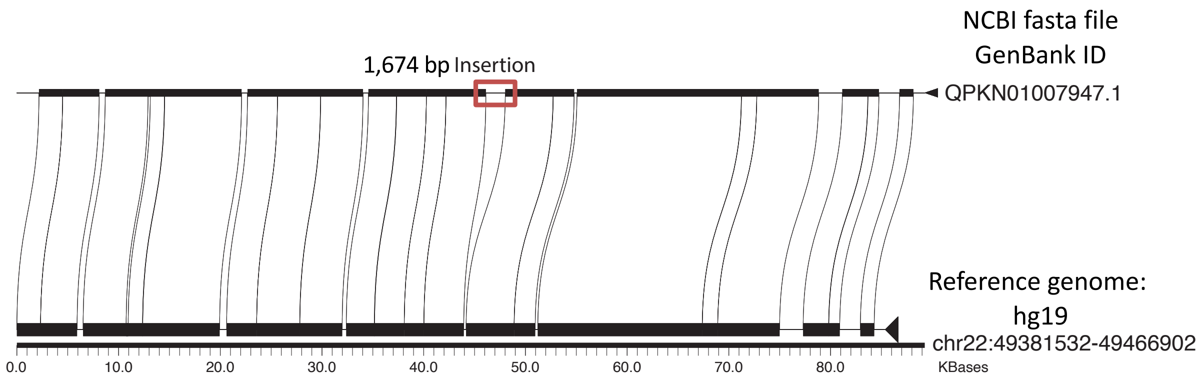

Figure S23. Insertion was characterized with PacBio long-read sequencing. QPKN01007947.1 contig [43] mapped to reference genome chr22: 49,381,532 – 49,466,902 with Miropeats [123] used for visualization. The orange box shows the clear insertion of 1,674 bp in length, comparing the QPKN01007947.1 contig with the reference genome.

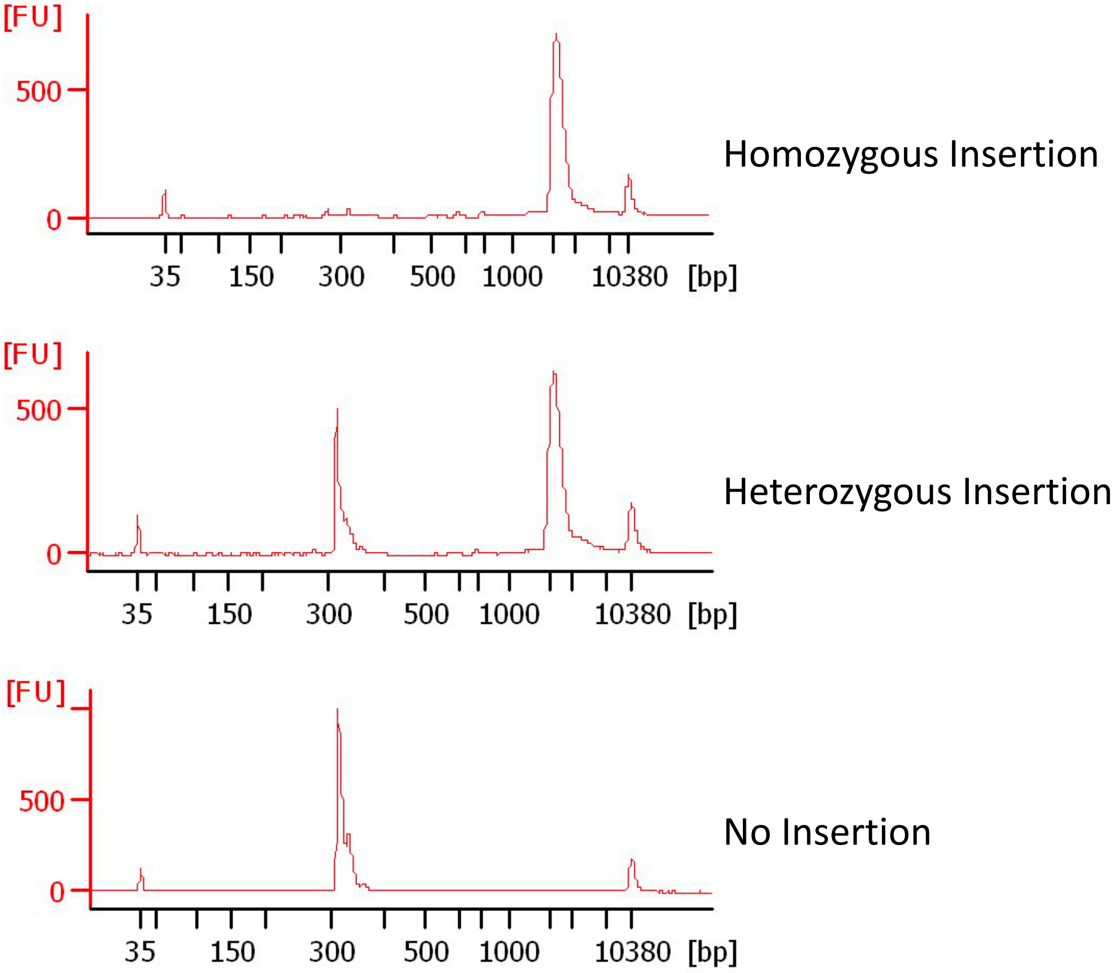

Figure S24. Insertion profiles were validated using PCR genotyping on the same sample. Genotyping primers were designed flanking both sides of the insertion to discriminate the alleles based on size following PCR. Agarose gel electrophoresis and bioanalyzer shown the same result (discovery group, ASD n = 41, TD n = 37).

QPKN01007947.1:46099-47772 (SVG Plot; Alignments; Masked)

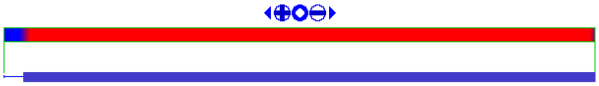

Name	From	To	Name	From	To	Class	Dir	Sim	Pos/Mm:Ts	Score
QPKN01007947.1:46099-47772	59	1673	SVA_A	1	1640	NonLTR/SINE	d	0.9514	1.4043	10850

Masked Sequence

```
>QPKN01007947.1:46099-47772
TAAGAAAACTCCTGCTCTCCCTCCTCTCCCTCTCCCTCCTCTCCCTCTCCCTCTCCCTXXXXXXXXXXXX
XXXXXXXXXXXXXXXXXXXXXXXXXXXXXXXXXXXXXXXXXXXXXXXXXXXXXXXXXXXXXXXXXXXXXX
XXXXXXXXXXXXXXXXXXXXXXXXXXXXXXXXXXXXXXXXXXXXXXXXXXXXXXXXXXXXXXXXXXXXXX
XXXXXXXXXXXXXXXXXXXXXXXXXXXXXXXXXXXXXXXXXXXXXXXXXXXXXXXXXXXXXXXXXXXXXX
XXXXXXXXXXXXXXXXXXXXXXXXXXXXXXXXXXXXXXXXXXXXXXXXXXXXXXXXXXXXXXXXXXXXXX
XXXXXXXXXXXXXXXXXXXXXXXXXXXXXXXXXXXXXXXXXXXXXXXXXXXXXXXXXXXXXXXXXXXXXX
XXXXXXXXXXXXXXXXXXXXXXXXXXXXXXXXXXXXXXXXXXXXXXXXXXXXXXXXXXXXXXXXXXXXXX
XXXXXXXXXXXXXXXXXXXXXXXXXXXXXXXXXXXXXXXXXXXXXXXXXXXXXXXXXXXXXXXXXXXXXX
XXXXXXXXXXXXXXXXXXXXXXXXXXXXXXXXXXXXXXXXXXXXXXXXXXXXXXXXXXXXXXXXXXXXXX
XXXXXXXXXXXXXXXXXXXXXXXXXXXXXXXXXXXXXXXXXXXXXXXXXXXXXXXXXXXXXXXXXXXXXX
XXXXXXXXXXXXXXXXXXXXXXXXXXXXXXXXXXXXXXXXXXXXXXXXXXXXXXXXXXXXXXXXXXXXXX
XXXXXXXXXXXXXXXXXXXXXXXXXXXXXXXXXXXXXXXXXXXXXXXXXXXXXXXXXXXXXXXXXXXXXX
XXXXXXXXXXXXXXXXXXXXXXXXXXXXXXXXXXXXXXXXXXXXXXXXXXXXXXXXXXXXXXXXXXXXXX
XXXXXXXXXXXXXXXXXXXXXXXXXXXXXXXXXXXXXXXXXXXXXXXXXXXXXXXXXXXXXXXXXXXXXX
XXXXXXXXXXXXXXXXXXXXXXXXXXXXXXXXXXXXXXXXXXXXXXXXXXXXXXXXXXXXXXXXXXXXXX
XXXXXXXXXXXXXXXXXXXXXXXXXXXXXXXXXXXXXXXXXXXXXXXXXXXXXXXXXXXXXXXXXXXXXX
XXXXXXXXXXXXXXXXXXXXXXXXXXXXXXXXXXXXXXXXXXXXXXXXXXXXXXXXXXXXXXXXXXXXXX
XXXXXXXXXXXXXXXXXXXXXXXXXXXXXXXXXXXXXXXXXXXXXXXXXXXXXXXXXXXXXXXXXXXXXX
XXXXXXXXXXXXXXXXXXXXXXXXXXXXXXXXXXXXXXXXXXXXXXXXXXXXXXXXXXXXXXXXXXXXXX
XXXXXXXXXXXXXXXXXXXXXXXXXXXXXXXXXXXXXXXXXXXXXXXXXXXXXXXXXXXXXXXXXXXXXX
XXXXXXXXXXXXXXXXXXXXXXXXXXXXXXXXXXXXXXXXXXXXXXXXXXXXXXXXXXXXXXXXXXXXXX
XXXXXXXXXXXXXXXXXXXXXXXXXXXXXXXXXXXXXXXXXXXXXXXXXXXXXXXXXXXXXXXXXXXXXX
XXXXXXXXXXXXXXXXXXXXXXXXXXXXXXXXXXXXXXXXXXXXXXXXXXXXXXXXXXXXXXXXXXXXXX
XXXXXXXXXXXXXXXXXXXXXXXXXXXXXXXXXXXXXXXXXXXXXXXXXXXXXXXXXXXXXXXA
```

Local Alignments*

Name	From	To	Name	From	To	Dir	Sim	Pos/Mm:Ts	Score
QPKN01007947.1:46099-47772	59	1673	SVA_A	1	1640	d	0.9514	1.4043	10850

```
59 CTCCCTCTCCCCACGGTCTCCCTCTCCCCACGGTCTCCCTCTCCCTCTCTTTCCACGGTCTCCCCCTGAT 128
   |||||||||||-----|| |||||||||||:|||||||||||||||||||||||||||||||||:|||||
 1 CTCCCTCTCCC-----TCACCCTCTCCCCATGGTCTCCCTCTCCCTCTCTTTCCACGGTCTCCCTCTGAT 65

129 GCTGAGCCGAAGCTGGACTGTACTGCTGCCATCTCGGCTCACTGCAACCTCCCTGCCTGATTCTCCTGCC 198
    ||:|||||||||||||| ||||||||||||||||||||||||||||||||||||||||||||||||||||
 66 GCCGAGCCGAAGCTGGACGGTACTGCTGCCATCTCGGCTCACTGCAACCTCCCTGCCTGATTCTCCTGCC 135

199 TCAGCCTGCCGAGTGCCTGCGATTGCAGGCGCGTGCCGCCACGCCTGACTGGTTTTCGTATTTTTTTGGT 268
    |||||:|||||||||||||||||||||||||:||||||||||||||||||||||||||||||| ||:||
136 TCAGCTTGCCGAGTGCCTGCGATTGCAGGCGCGCGCCGCCACGCCTGACTGGTTTTCGTATTTTGTTAGT 205

269 GGAGACGGGGTTTCGCTGTGTTGGCCGGGCTGGTCTCCAGCTCCTAATCACGAGTGATCCGCCAGCCTTG 338
    |||||||||||||||||||||||||||||||||||||||||||||||||:|:||||||||||:|||||||:|
206 GGAGACGGGGTTTCGCTGTGTTGGCCGGGCTGGTCTCCAGCTCCTAACCGCGAGTGATCCACCAGCCTCG 275

339 GCCTCCCGAGGTGCCGGGATTGCAGACGGAGTCTCGTTCACTCAGTGCTCAATGGTGCCCAGGCTGGAGT 408
    ||||||||||||||:||||||||||||||||||||||||||||||||||||||||:|||||||||||||||
276 GCCTCCCGAGGTGCTGGGATTGCAGACGGAGTCTCGTTCACTCAGTGCTCAATGATGCCCAGGCTGGAGT 345

409 GCAGTGGCGTGATCTCGGCTTGCTACAACCTCCACCTCCCAGCCGCCTGCCTTGGCCTCCCAAAGTGCCG 478
    ||||||||||||||||||||:|||||||||||||||||||| ||||||||||||||||||||||||||||
346 GCAGTGGCGTGATCTCGGCTCGCTACAACCTCCACCTCCCAGCAGCCTGCCTTGGCCTCCCAAAGTGCCG 415

479 AGATTGCAGCCTCTGCCCGGCCGCCACCCCGTCTGGGAAGTGAGGAGCGTCTCTGCCTGGCCGCCCATCG 548
    ||||||||||||||||||||||||||||||||||||||||||||||||:|||||:||||||||:|||||||
416 AGATTGCAGCCTCTGCCCGGCCGCCACCCCGTCTGGGAAGTGAGGAGTGTCTCCGCCTGGCCACCCATCG 485

549 TCTGGGACGTGAGGAGCCCCTCTGCCTGGCTGCCCAGTCTGGAAAGTGAGGAGCGTCTCTGCCCGGCCGC 618
    |||||||:|||||||||-------------------------------------|||||||||| |||||
486 TCTGGGATGTGAGGAGC-------------------------------------GTCTCTGCCCTGCCGC 518

619 CATCCCATCTAGGAAGTGAGGAGCGCCTCTGCCAGGCCGCCCATCGTCTGAGATGTGGGGAGCGCCTCTG 688
    |-----------------------------------------||||||||||||||||||||||:||||||
519 C-----------------------------------------CATCGTCTGAGATGTGGGGAGCACCTCTG 548

689 CCCTGCCACCCCGTCTGGGATGTGAGGAGCGTCTCTGCCCGGCCGCCCCGTCTGAGAAGTGAGGAGACCC 758
    ||| |||:|||||||:|||||||||||||||| ||||||||||||||||||||||||||||||||||||
549 CCCGGCCGCCCCGTCCGGGATGTGAGGAGCGTCGCTGCCCGGCCGCCCCGTCTGAGAAGTGAGGAGACCC 618

759 TCTGCCTGGCAACCGCCCCGTCTGAGAAGTGAGGAGCCCCTCCGCCCGGCAGCCACACCCTCTGAGAAGT 828
    ||||||||||||||||:||:||||||||||||||||||||||||||||||||||:| |: ||||||||||
619 TCTGCCTGGCAACCGCTCCATCTGAGAAGTGAGGAGCCCCTCCGCCCGGCAGCCGCCCTGTCTGAGAAGT 688

829 GAGGAGCGTCTCTGCCTGGCAGCCACCCCGTCTGGGAGGGAGGTGGGGG--TCAGCCCCCTGCCCCGCCA 896

965 GGGGCGCCTCTGCCCGGCCGCCCCTACTGGGAAGTGAGGAGCC--------------------------- 1007
    |||||||||||||||||||||||||||||||||||||||||||---------------------------
829 GGGGCGCCTCTGCCCGGCCGCCCCTACTGGGAAGTGAGGAGCCACTTTGCCCGGCCAGCCACTCTGTCCG 898

1008 ----------------------CCTCTGCCTGGCCAGCCGCCCCGTCGGGGAGGGAGGTGGGGGGA---- 1051
     ----------------------||:|:|||:||||||||||||||| |||||||||||||||||----
 899 GGAGGGAGGTGGGGGGGTCAGCCCCCGCCCGGCCAGCCGCCCCGTCCGGGAGGGAGGTGGGGGGATCAG 968

1052 ----------------------------------------------CAGCCCCCGCCCGGCCAGCCGCC 1075
     ----------------------------------------------|||||||||||||||||||||||
 969 CCCCCCGCCCAGCCAGCCGCCCCGTCCGGGAGGGAGGTGGGGGGTCAGCCCCCGCCCGGCCAGCCGCC 1038

1076 CCGTCCGGGAGGTGAGGGGCGCCTCTGCCCGGCCGCCCCTACTGGGAAGTGAGGAGCCCCTCTGCCCGGC 1145
     |:|||||||||||||||||||||||||||||||||| ||||||||:||||||||||||||||||||||||
1039 CTGTCCGGGAGGTGAGGGGCGCCTCTGCCCGGCCGCGCCTACTGGAAAGTGAGGAGCCCCTCTGCCCGGC 1108

1146 CACCACCCCGTCTGGGAGGTGTACTCAACAGCTCATTGAGAACGGGCCATGATGACAATGGCGGTTTTGT 1215
     ||||||||||||||||||||||:|:||||||||||||||||| |||||||||||||||||||||||||||
1109 CACCACCCCGTCTGGGAGGTGTGCCCAACAGCTCATTGAGAAGGGGCCATGATGACAATGGCGGTTTTGT 1178

1216 GGAATAGAAAGGGGGGAAAGGTGGGGAAAAGATTGAGAAATCGGATGGTTGCCGTGTCTGTGTAGAAAGA 1285
     ||||||||||||||||||||||||||||||||||||||||||||||||||||||||||||||||||||||
1179 GGAATAGAAAGGGGGGAAAGGTGGGGAAAAGATTGAGAAATCGGATGGTTGCCGTGTCTGTGTAGAAAGA 1248

1286 GGTAGACATGGGAGACTTTTCATTTTGTTCTGTACTAAGAAAAATTCTTCTGCCTTGGGATCCTGTTGAT 1355
     ||||||| ||||||||||||||||||||||||||||||||||||||||||||||||||||||||||||||
1249 GGTAGACCTGGGAGACTTTTCATTTTGTTCTGTACTAAGAAAAATTCTTCTGCCTTGGGATCCTGTTGAT 1318

1356 CTGTGACCTTACCCCCAACCCTGTGCTCTCTGAAACATGTGCTGTGTCCACTCAGGGTTGAATGGATTAA 1425
     | ||||||||||||||||||||||||||||||||||||||||||:|||||||||||||||||||||||||
1319 CGGTGACCTTACCCCCAACCCTGTGCTCTCTGAAACATGTGCTGTATCCACTCAGGGTTGAATGGATTAA 1388

1426 GGGTGGTGCAAGATGTGCTTTGTTAAACAGATGCTTGAAGGCAGCATGCTCGTTAAGAGTCATCACCACT 1495
     |:|:||||||||||||||||||||||||||||||||||||||||||||||| ||||||||||||||||||
1389 GAGCGGTGCAAGATGTGCTTTGTTAAACAGATGCTTGAAGGCAGCATGCTCCTTAAGAGTCATCACCACT 1458

1496 CCCTAATCTCAAGTACCCAGGGACACAAACACTGCGGAAGGCCGCAGGGTCCTCTGCCTAGGAAAACCAG 1565
     ||||||||||||||||||||||||||||||||||||||||||||||||||||||||||||||||||||||
1459 CCCTAATCTCAAGTACCCAGGGACACAAACACTGCGGAAGGCCGCAGGGTCCTCTGCCTAGGAAAACCAG 1528

1566 AGACCTTTGTTCACTT----ATCTGCTGACCTTCCCTCCACTATTGTCCTGTGACCCTGCCAAATCCCCC 1631
     ||||||||||||||||----||||||||||||||||||||||||||||||||||||||||||||||||||
1529 AGACCTTTGTTCACTTGTTTATCTGCTGACCTTCCCTCCACTATTGTCCTGTGACCCTGCCAAATCCCCC 1598

1632 TCTGCGAGAAACACCCAAGAATGATCAATAAAAAAAAAAAAAA 1673
     ||||:||||||||||||||||||||||||||||||||||||||
1599 TCTGTGAGAAACACCCAAGAATGATCAATAAAAAAAAAAAAAA 1640
```

Figure S25. Insertion contained retrotransposon elements.
Insertion sequence was used to query against the collection of repeat databases with default settings.

Structural variant: INS_22_115103

Dataset gnomAD SVs v2.1

Filter Pass
Allele Count 14350
Allele Number 20614
Allele Frequency 0.6961
Quality score 999
Position 22:49425456
Size 1,314 bp
Class insertion (ME:SVA)
Evidence Split reads
Algorithms MELT

External Resources

- UCSC

Feedback

Report an issue with this variant

Consequences

This variant has consequences in 0 genes.

Genotype Quality

Variant carriers All individuals

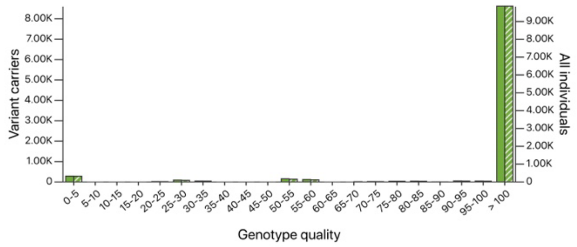

Compare to all individuals

Population Frequencies

Population	Allele Count	Allele Number	Number of Homozygotes	Allele Frequency
Latino	1178	1470	463	0.8014
European	5437	7452	1908	0.7296
African/African-American	6121	9086	1988	0.6737
Other	125	190	40	0.6579
East Asian	1489	2416	404	0.6163
XX	7131	10132	2422	0.7038
XY	7189	10444	2369	0.6883
**Total**	**14350**	**20614**	**4803**	**0.6961**

Age Distribution

Variant carriers All individuals

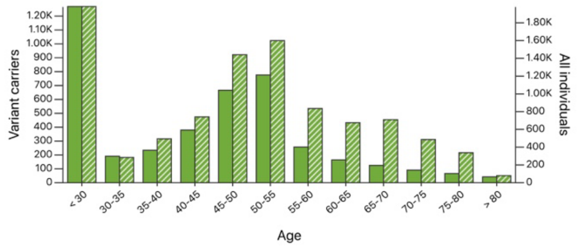

Include heterozygous variant carriers
Include homozygous variant carriers
Compare to all individuals

Figure S26. The insertion was also identified in the gnomAD database.
gnomAD dataset [45,46] snapshot of the insertion named as INS_22_115103, classified as insertion structural variant with SVA element.

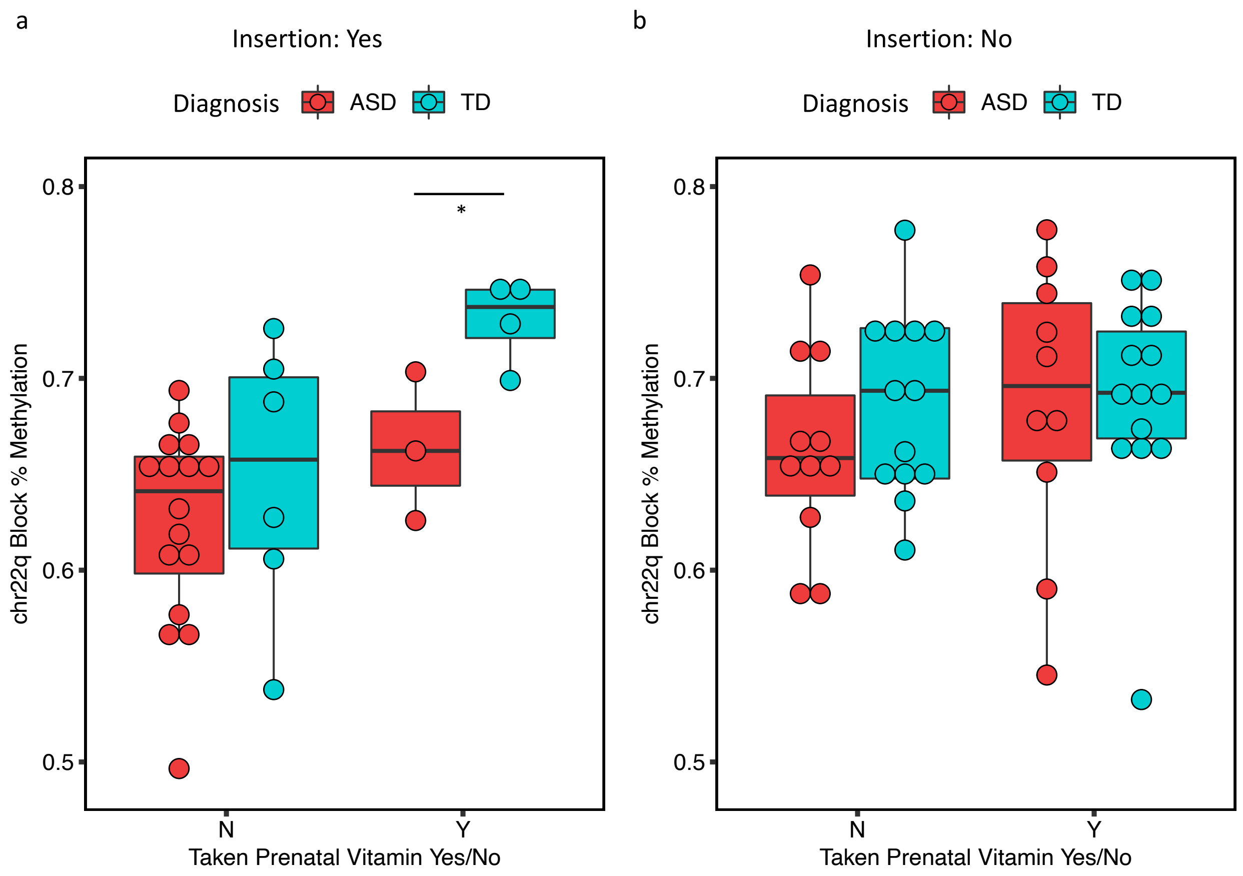

Figure S27. When separated by insertion genotype, methylation levels at 22q13.33 were most different between TD and ASD in offspring homozygous for the insertion whose mothers took a prenatal vitamin in month 1 of pregnancy.

In other words, for individuals with the genetic risk of the insertion, taking prenatal vitamins at P1 significantly altered 22q13.33 block methylation, in the protective direction (Mann-Whitney-Wilcoxon, *p*-value = 0.046, ASD n = 3, TD n = 4, effect size = -2.09), a difference which was not significant in in those with the insertion whose mothers did not take a prenatal vitamin (*p*-value = 0.407).

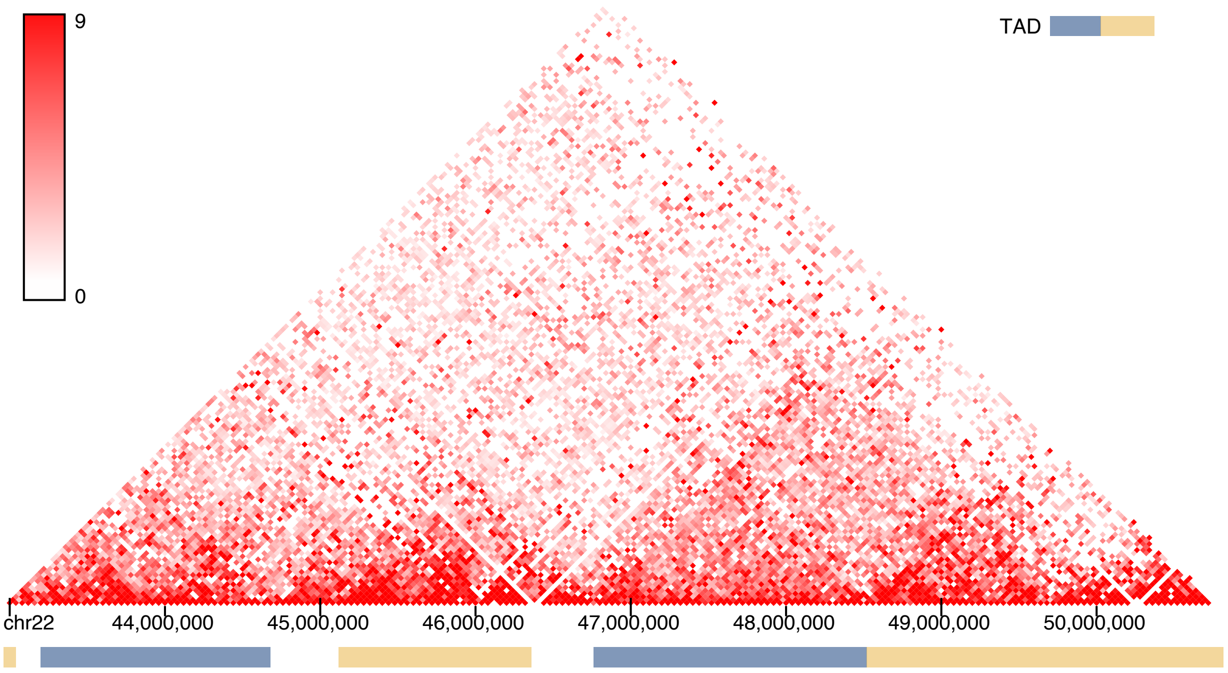

Figure S28. Topologically associated domain (TAD) from hippocampus on 22q13.31 – 22q13.33 regions that extends to the telomere, assembly in hg38 with resolution at 40 kb [53].

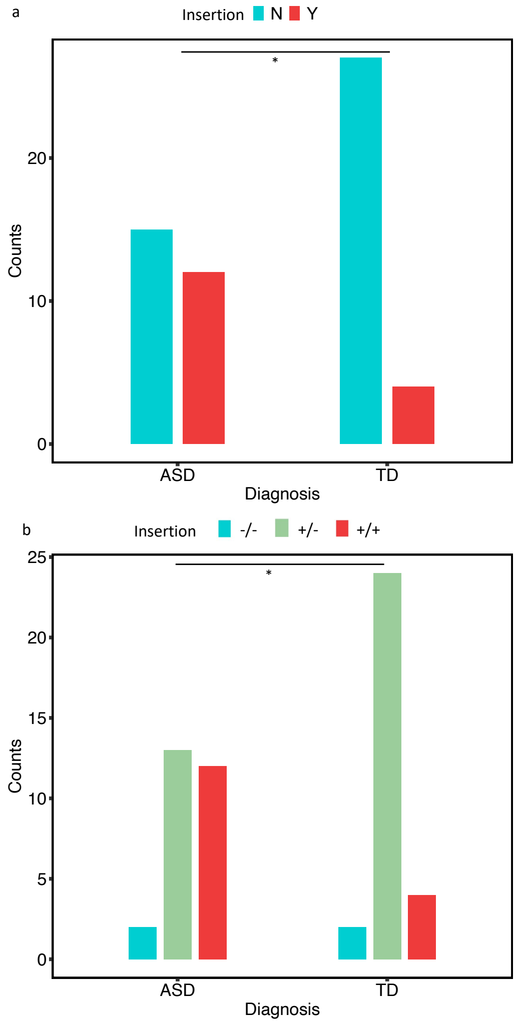

Figure S29. The insertion had significantly higher frequency in ASD compared with TD samples in brain.

(**a**) Count matrix was based on the Y/N insertion genotypes in brain, showing a higher frequency of individuals with the insertion in ASD compared to TD (ASD n = 27, TD n =30) (chi-square test, *p*-value = 0.023).

(**b**) Count matrix was based on -/-, +/-, +/+ insertion genotypes in brain, showing a higher frequency of individuals with homozygosity for the insertion (+/+) in ASD compared to TD (ASD n = 27, TD n =30) (chi-square test, *p*-value = 0.028).

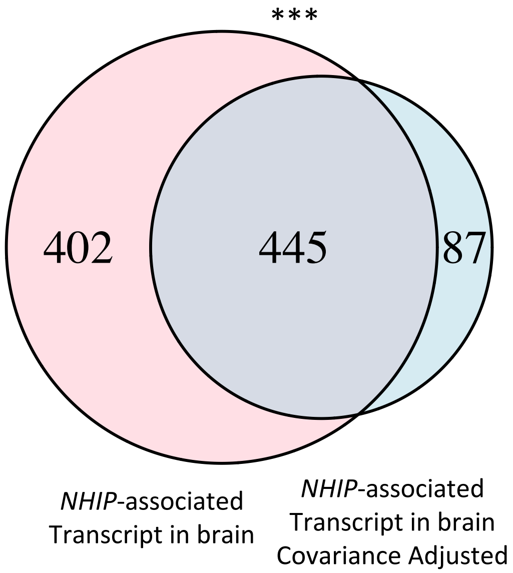

Figure S30. *NHIP*-associated genes in brain adjusted for covariates (age, sex, region, PMI, race, ethnicity) significantly overlapped with *NHIP*-associated genes compared to all expressed genes (Fisher's exact test, *p*-value < 2.2E-16, OR = 248.454).

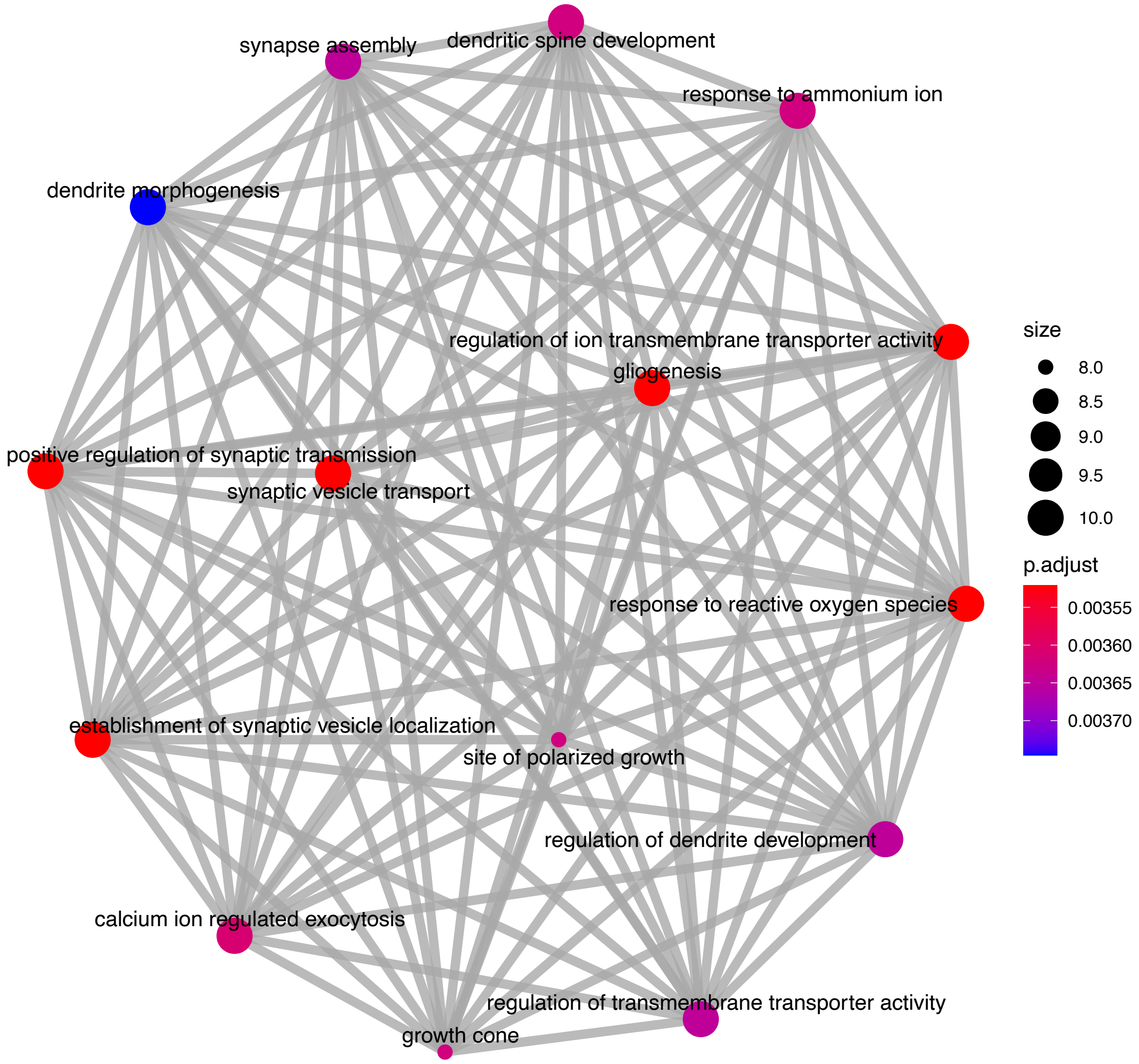

Figure S31. Genes significantly associated with *NHIP* levels were enriched for neuronal functions.

GO terms analysis was based on the genes showing significant *NHIP* association in brain after adjustment for potential covariates. The enrichment map organized significantly enriched terms into a network with edges connecting overlapping gene sets. Clustered gene sets were identified as functional modules with mutually overlapping genes.

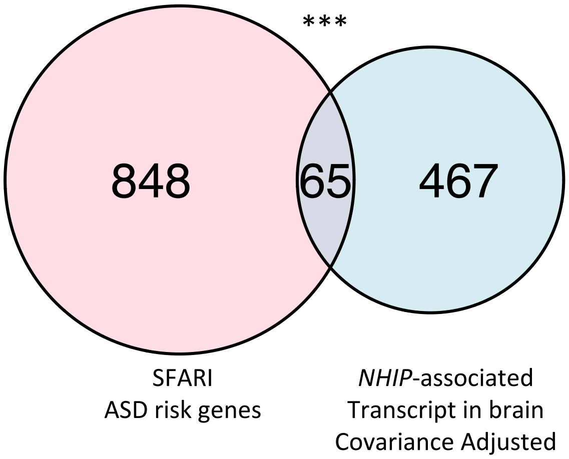

Figure S32. *NHIP*-associated genes in brain (adjusted for potential covariates) show a significant overlap with SFARI ASD risk genes compared to all expressed genes (Fisher's exact test, *p*-value < 0.001).

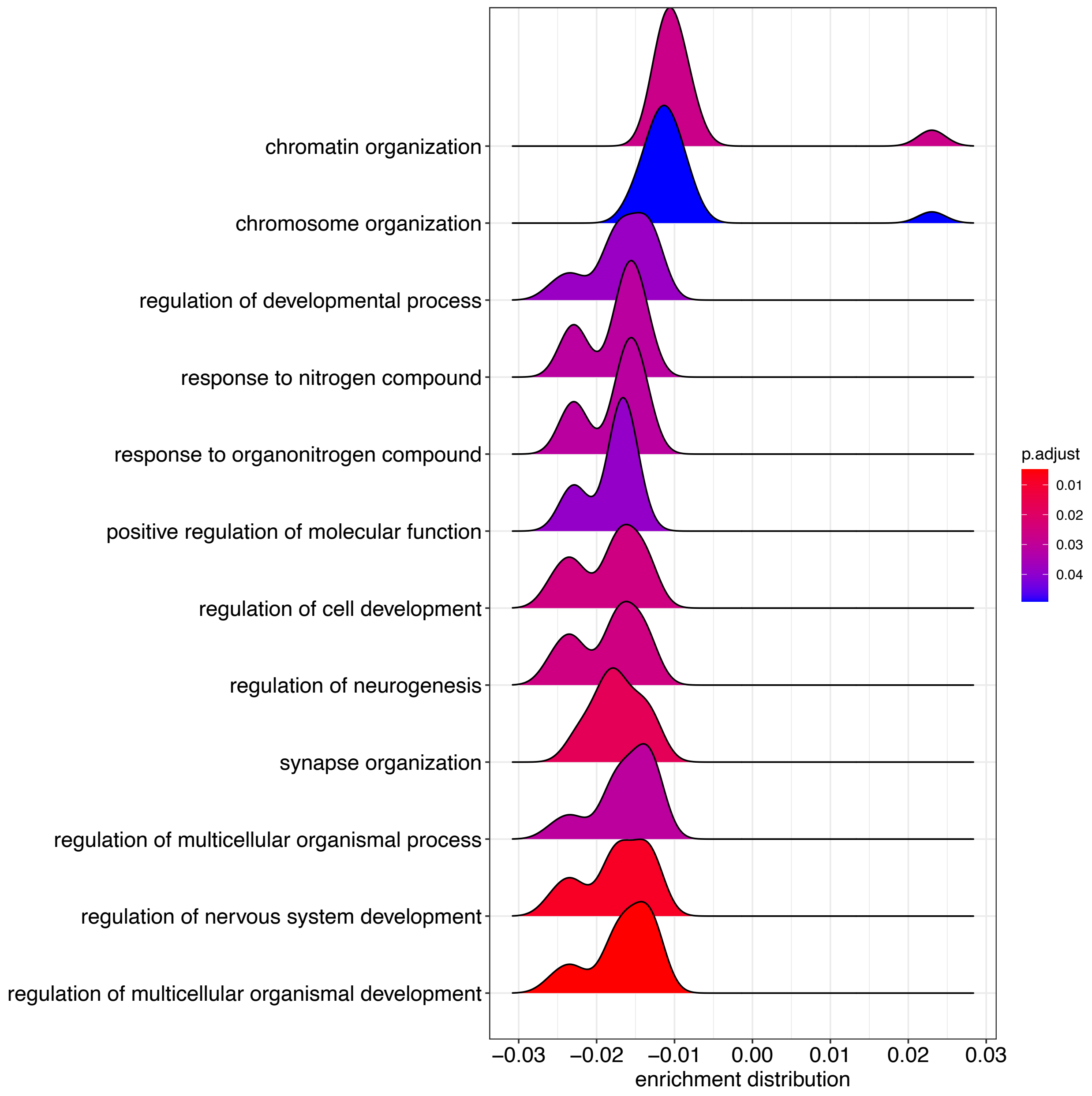

Figure S33. Genes in common between brain DGE and SFARI ASD genes show enrichment for chromatin, synapse, and nervous system development.
GO term analysis was based on the 65 genes in common between *NHIP* association in brain and SFARI ASD genes. The ridgeline plot was generated using the frequency of fold change values per gene within each enrichment GO term set.

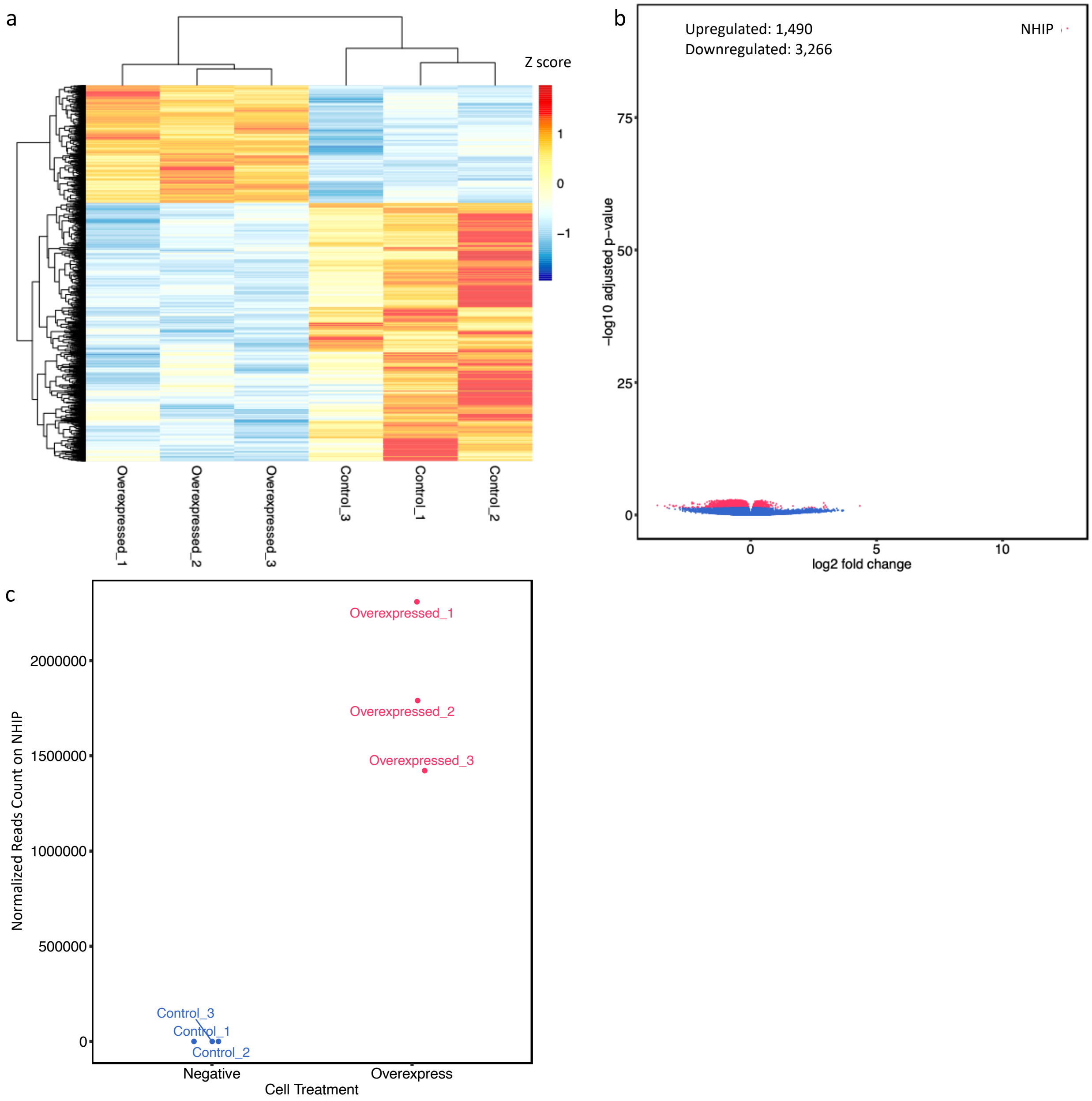

Figure S34. RNA-seq from overexpression of *NHIP* versus negative control cells, identifying 4,756 differential expressed genes (FDR 0.05 corrected).

(**a**) Heatmap and hierarchical clustering of 3 overexpressed *NHIP* and 3 negative control with clear separation by condition.

(**b**) Volcano plot showing 4,756 DGEs including 1,490 upregulated and 3,266 downregulated genes.

(**c**) Graph to illustrate the difference in *NHIP* transcript levels between overexpressed and negative control cells.

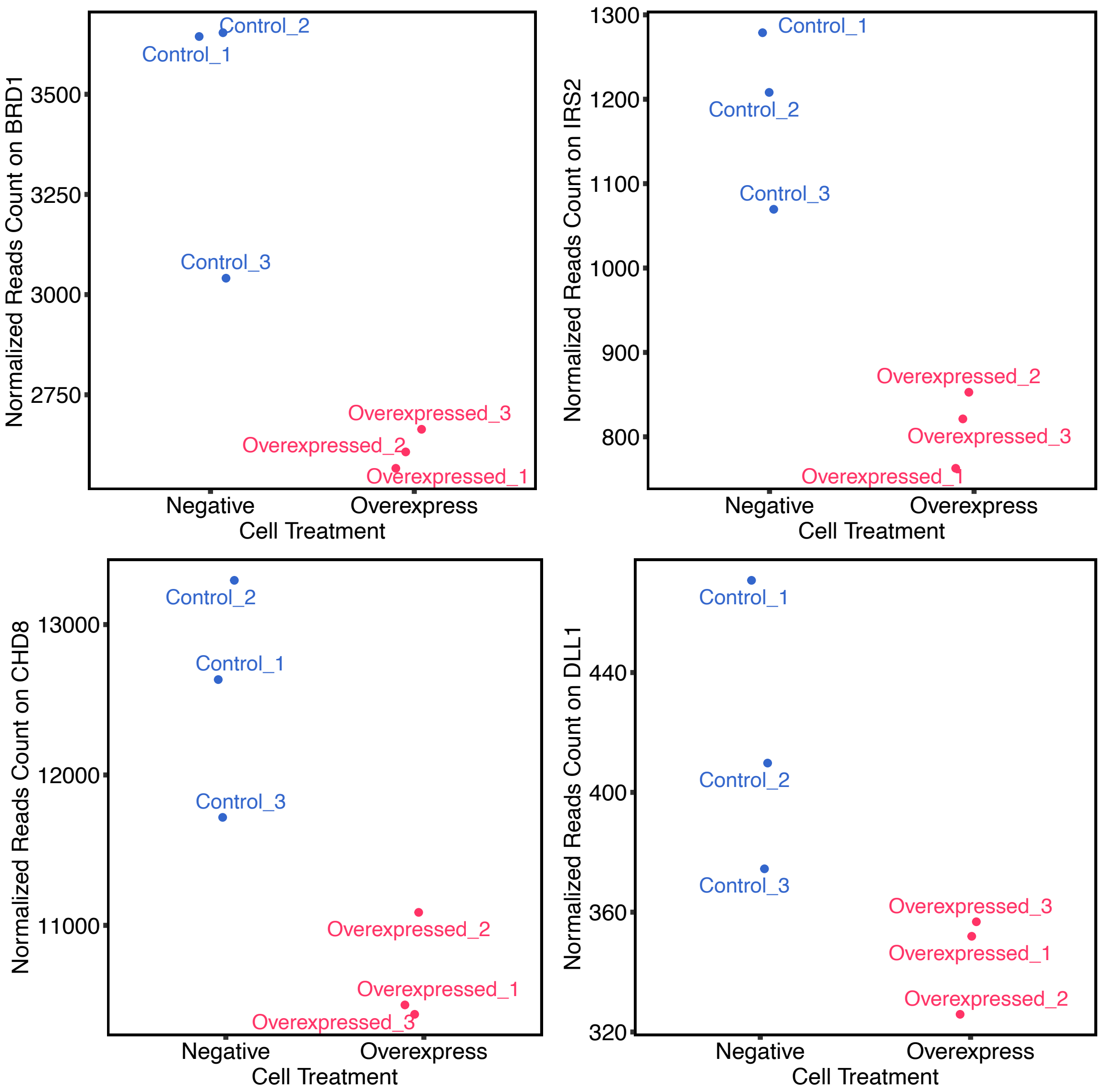

Figure S35. Several genes were downregulated with *NHIP* overexpression in HEK293T cells. Normalized read counts from RNA-seq are plotted on the y-axis, while x-axis shows cell treatment. *BRD1* (adjusted *p*-value: 0.004), *IRS2* (adjusted *p*-value: 0.003), *CHD8* (adjusted *p*-value: 0.003), and *DLL1* (adjusted *p*-value: 0.048) were significantly downregulated in overexpression *NHIP* cells. Additional File 22: Table S21 contains additional details of RNA-seq statistical analyses.

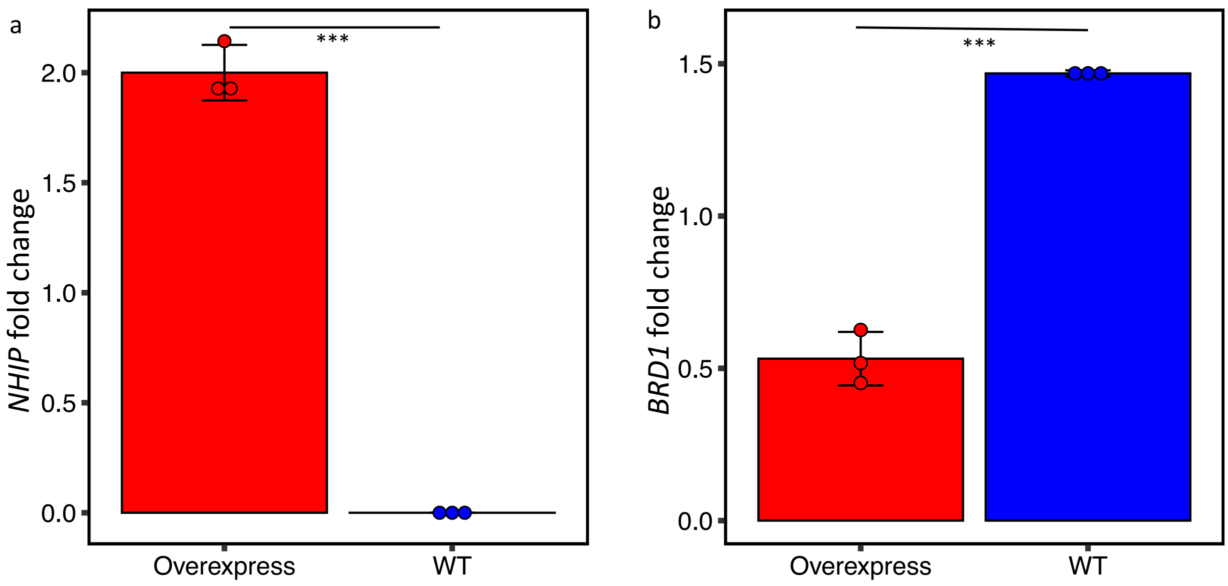

Figure S36. *NHIP* overexpression related changes to *BRD1* were validated using RT-qPCR. To validate the results from RNA-seq, RT-qPCR was performed on *NHIP* and *BRD1*. *NHIP* was significantly upregulated in the *NHIP* overexpressing cell line (Mann-Whitney-Wilcoxon, *p*-value = 0.001). *BRD1* was significantly downregulated in the overexpressing cell line (Mann-Whitney-Wilcoxon, *p*-value = 0.002).

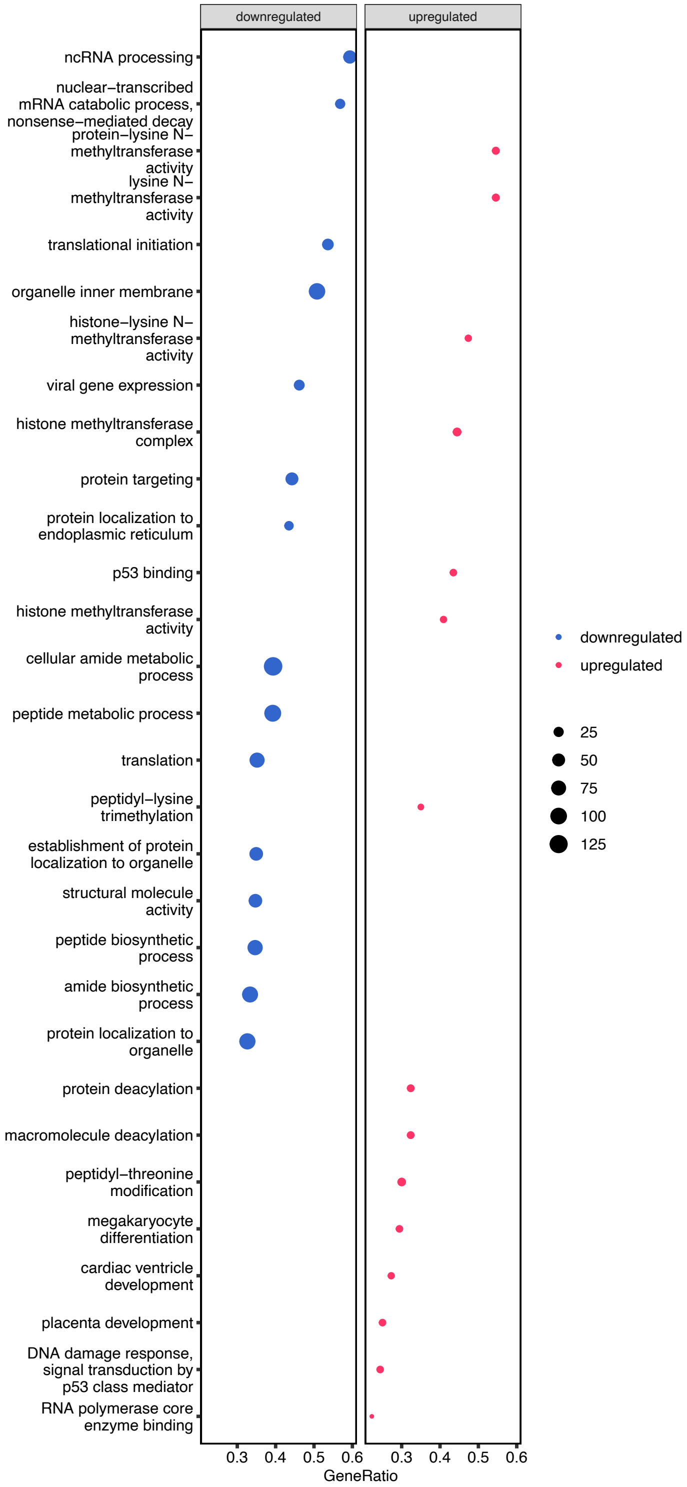

Figure S37. Overexpression of *NHIP* in HEK293T cells led to transcriptional changes in genes with functions in histone methylation and cell cycle.

GO analysis was based on the 4,756 DGE comparing *NHIP* overexpression and negative control cells with significant enrichment colored in red and blue to represent upregulated and downregulated GO terms, respectively (FDR adjusted *p*-value < 0.05).

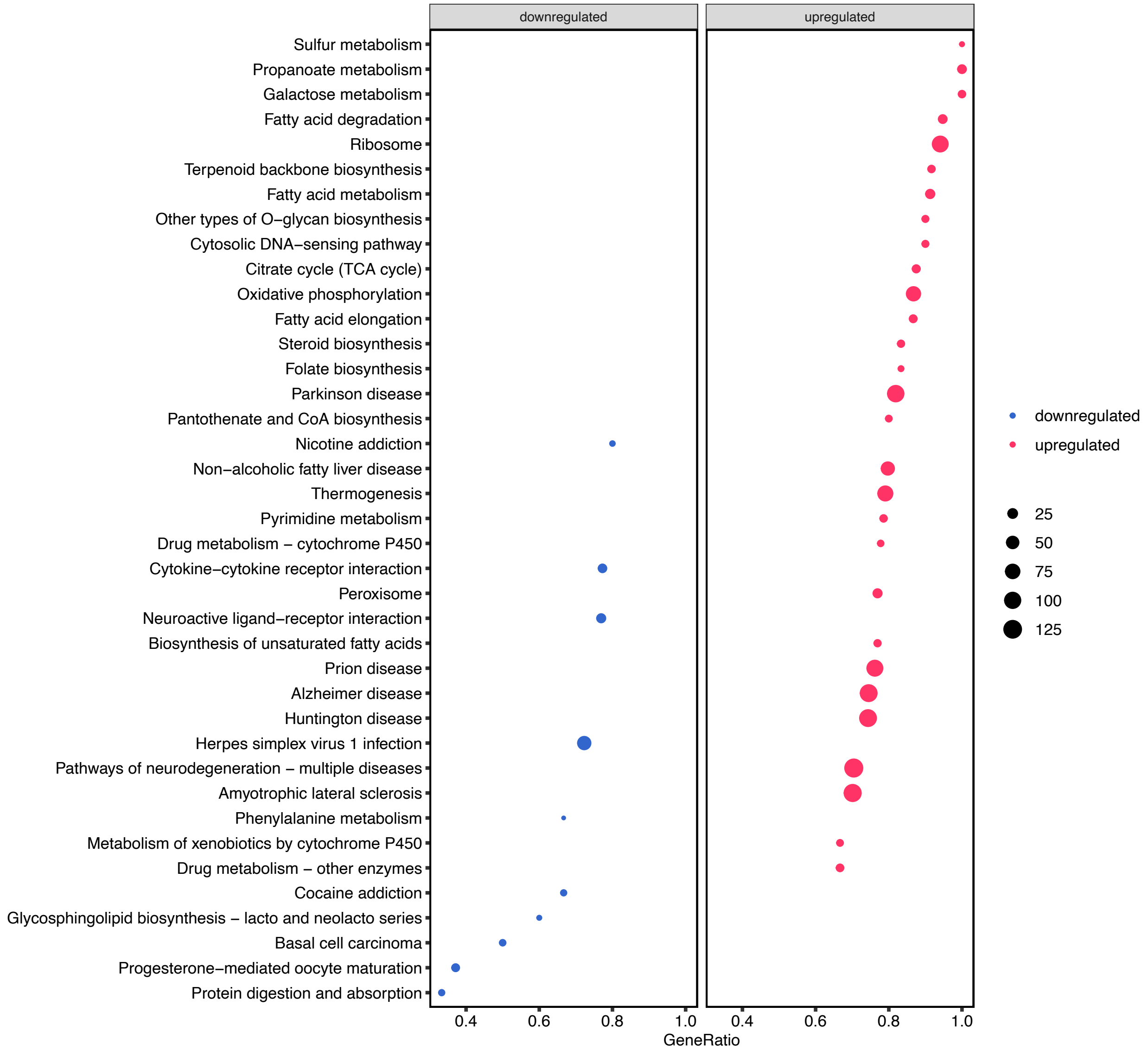

Figure S38. Genes significantly associated with overexpression of *NHIP* in HEK293T cells were enriched for neurological diseases.

Enrichment analyses of DGE in cells were also compared to Kyoto Encyclopedia of Genes and Genomes (KEGG) [55] gene sets using the clusterProfiler package [131]. Several terms related with the neurological diseases Parkinson's disease, Alzheimer's disease, and Huntington's disease were upregulated after overexpressing *NHIP* in HEK293T cells. Metabolism terms, including fatty acid and drug metabolism, were also upregulated with *NHIP* overexpression. GO analysis was based on the 4,756 DGE from *NHIP* overexpression compared to negative control cells, with significant enrichment colored in red for upregulated or blue for downregulated GO terms (FDR adjusted *p*-value < 0.05).

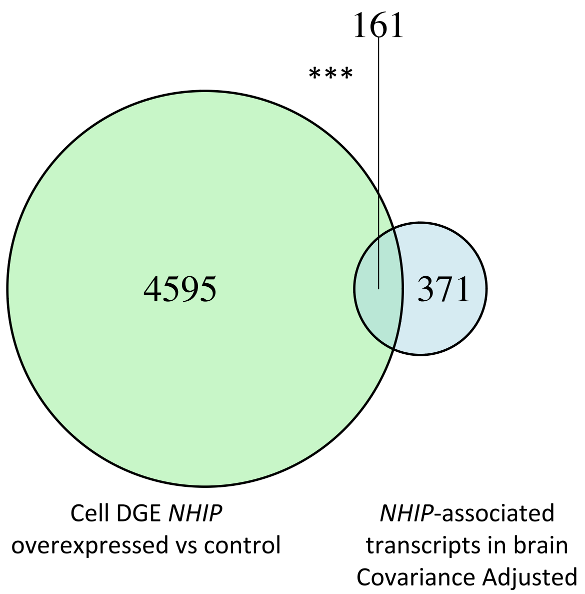

Figure S39. Genes differentially expressed in *NHIP* overexpressing cells significantly overlapped with *NHIP* associated genes identified in brain compared to all expressed genes (Fisher's exact test, *p*-value < 0.001), using brain RNA-seq data adjusted for sex, age, brain region, and postmortem interval.

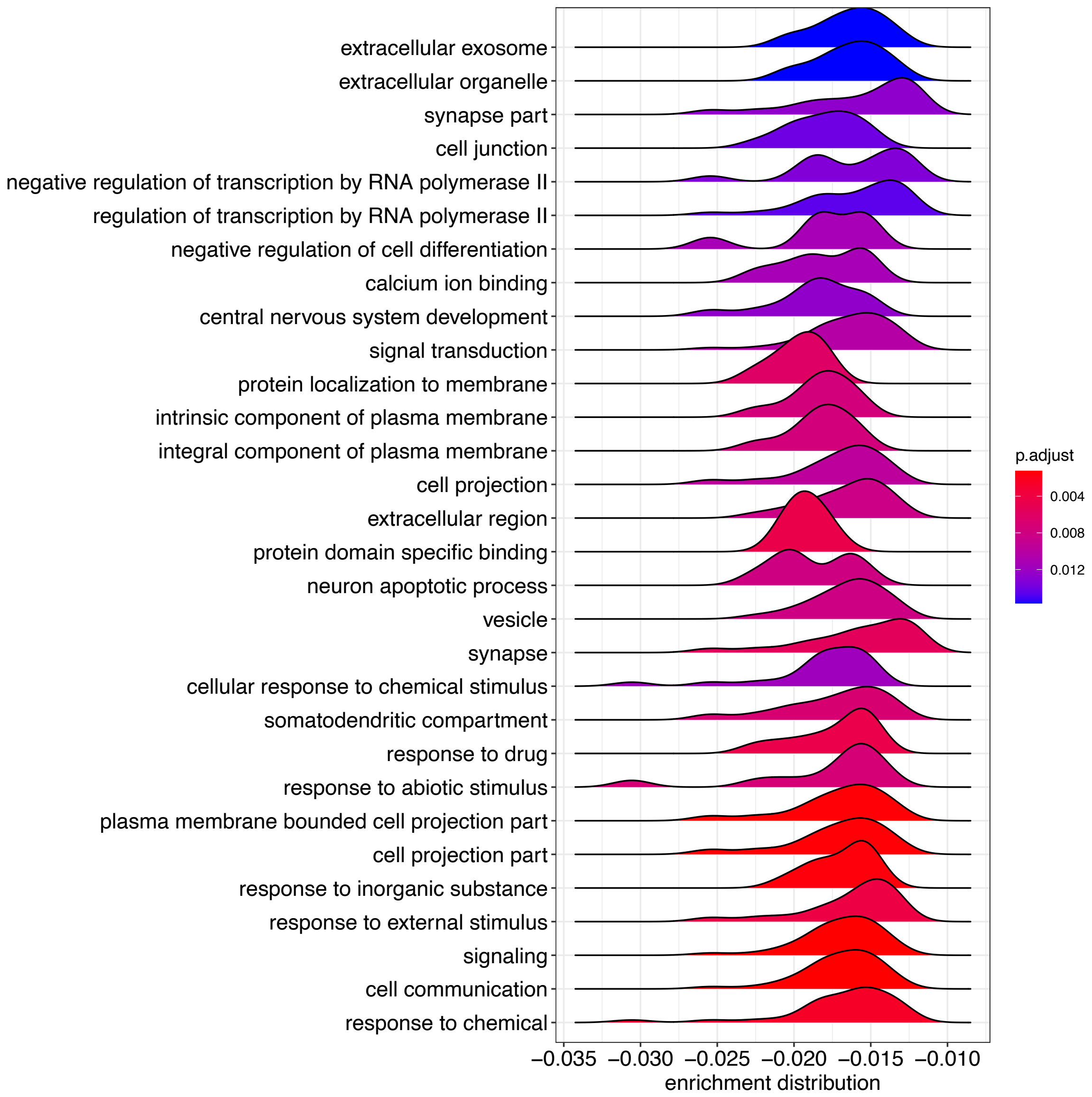

Figure S40. Genes in common between *NHIP* DGE in cell and *NHIP* associated in brain (adjusted) were significantly enriched for neuronal functions.

GO term analysis of the overlapping 161 genes showed enrichment for neuronal functions, including dendrite, nervous system development, and regulation of neurogenesis. The ridgeline plot was generated using the frequency of fold change values per gene within each enrichment GO term set.

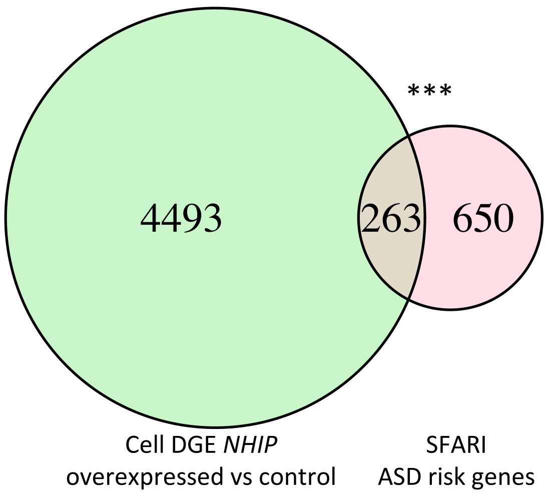

Figure S41. Genes differentially expressed in *NHIP* overexpressing cells significantly overlapped with SFARI ASD genes compared to all expressed genes (Fisher's exact test, *p*-value < 0.001).

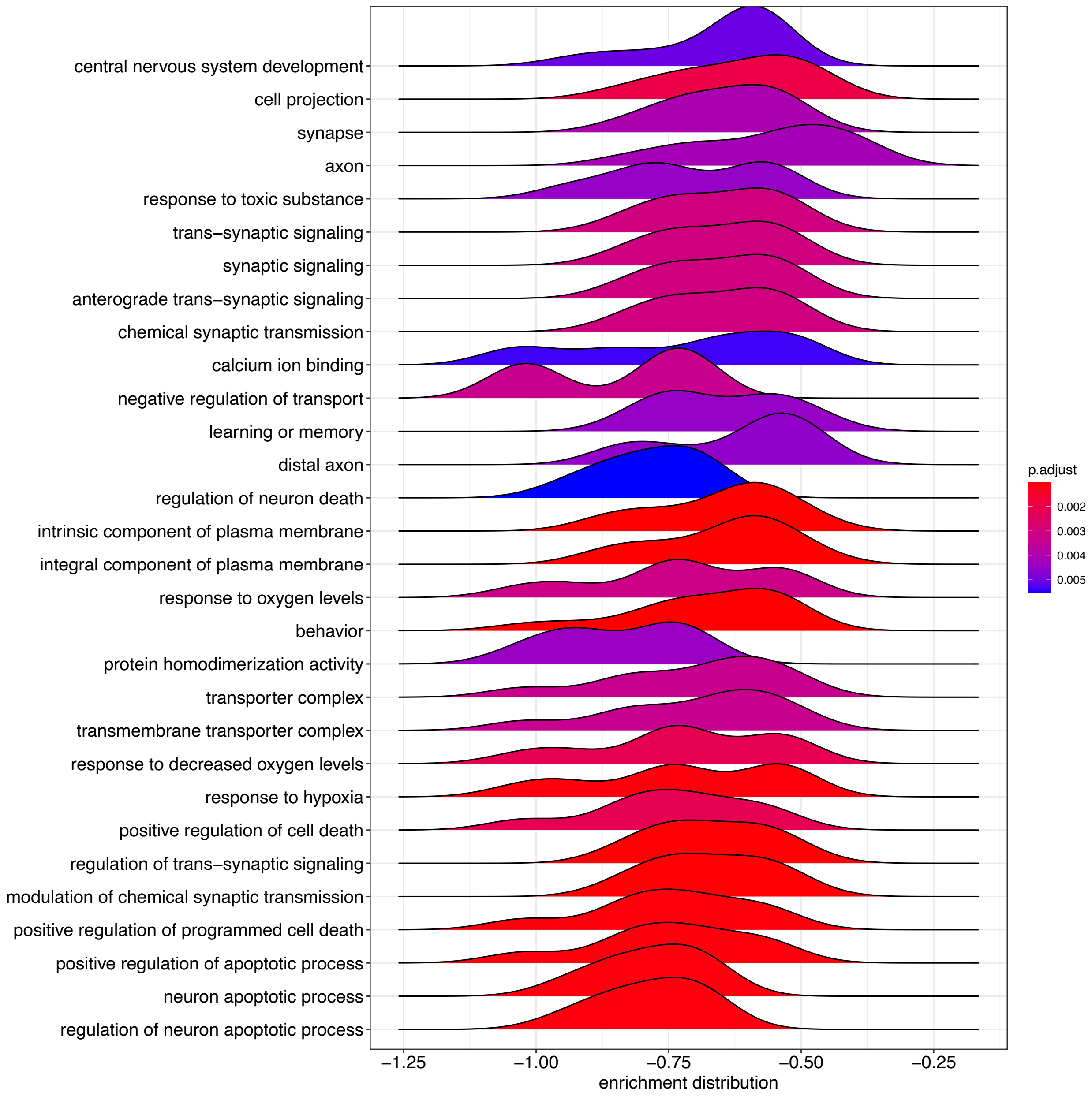

Figure S42. Genes in common between cell line NHIP overexpression DGE and SFARI ASD genes are enriched for neuronal functions and response to oxygen levels. GO term analysis was based on the 263 genes in common between significant DGE in the HEK293T overexpression *NHIP* and SFARI ASD genes. The ridgeline plot was generated using the frequency of the fold change values per gene within each enriched GO term.

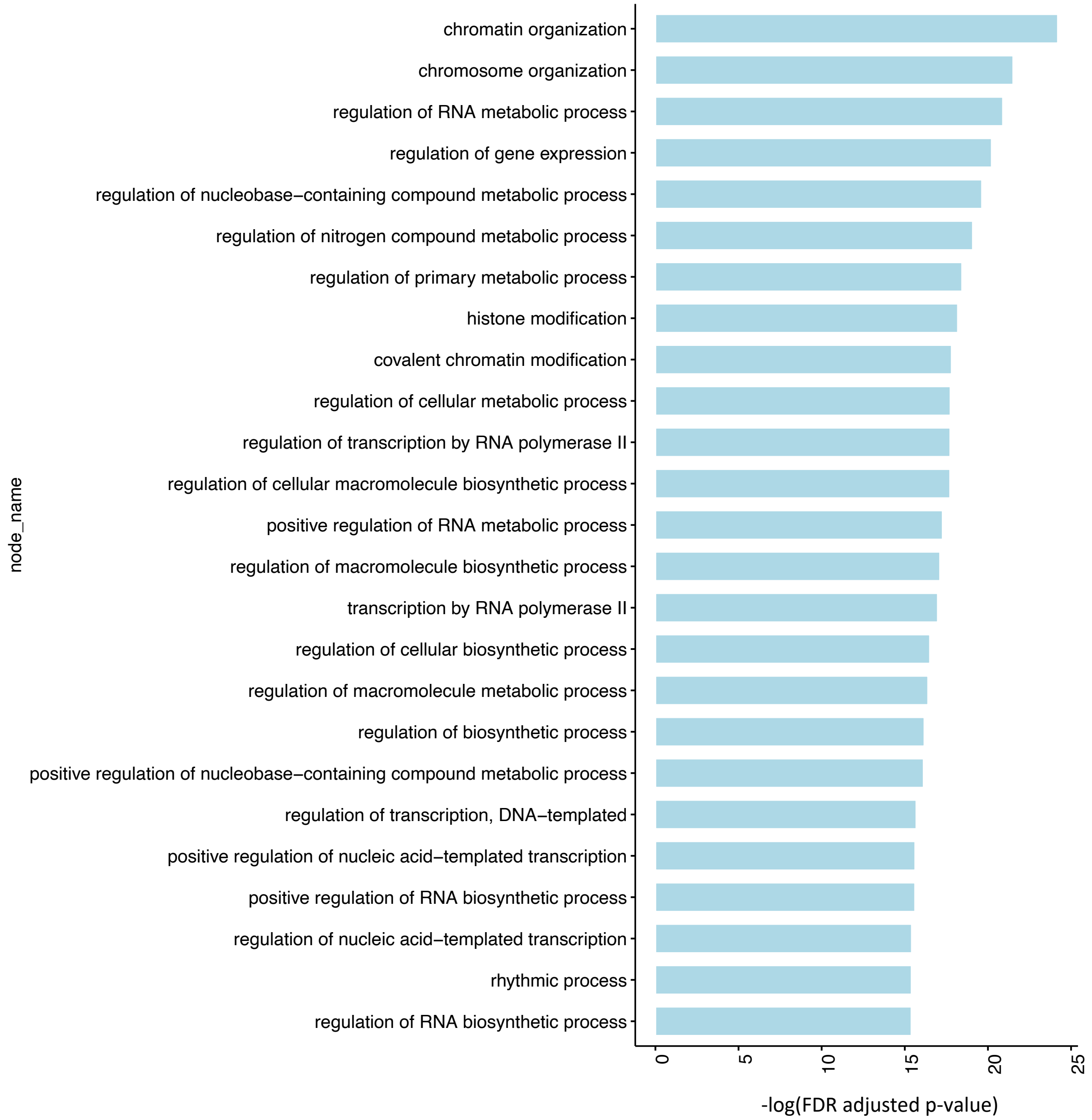

Figure S43. Genes in common among brain *NHIP*-associated (adjusted), cell line DGE, and SFARI ASD genes are enriched for histone modification and rhythmic process functions.
GO term analysis was based on the common 30 genes among brain DGE, cell line DGE, and SFARI ASD genes. The bar plot was generated by the top 25 GO terms in the biological process category and shown enrichment with histone acetyltransferase.

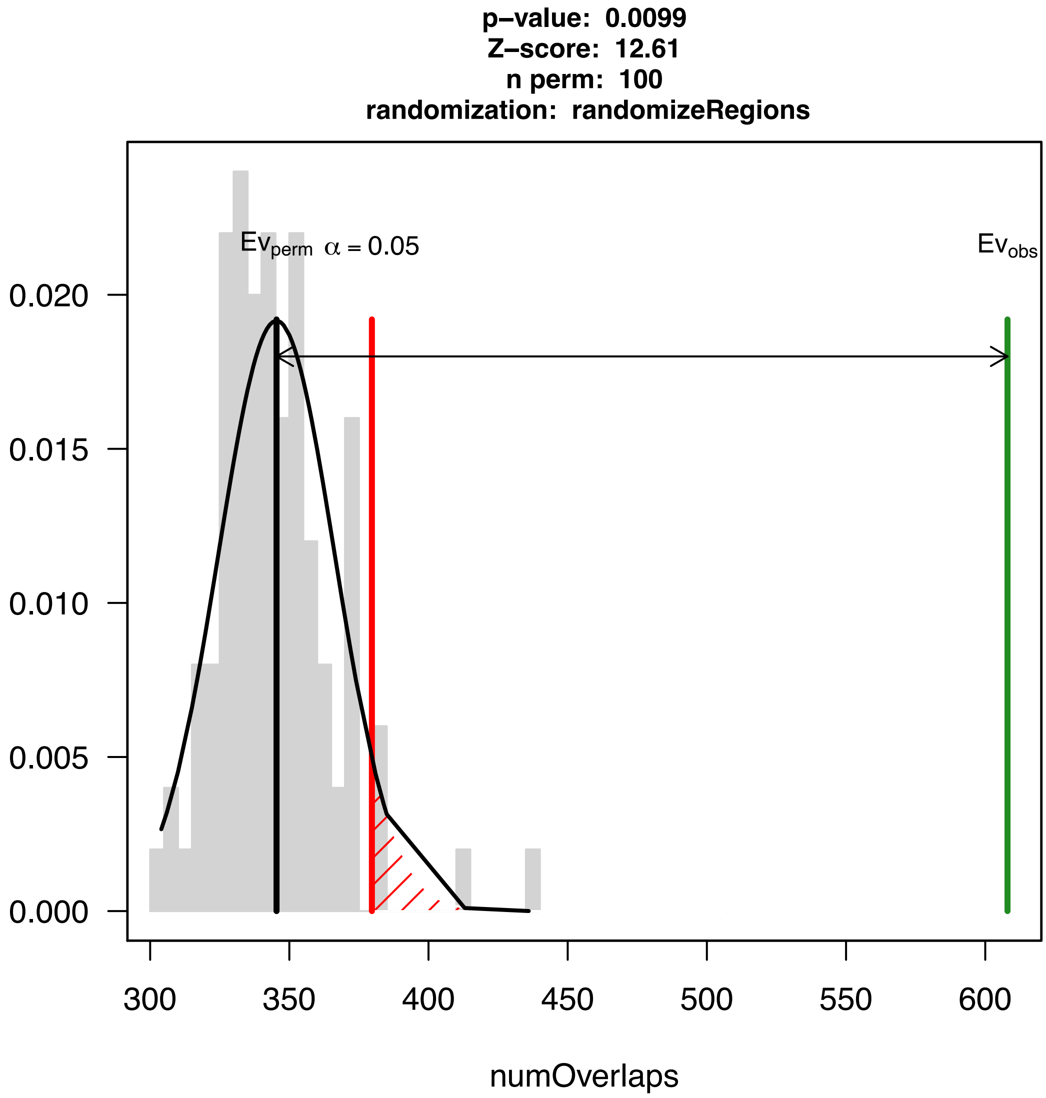

Figure S44. CoRSIV regions significantly overlapped with SV hotspots.
Analysis was performed using the regioneR R package with 100 permutation tests to show the significant overlapping between CoRSIV regions and SV hotspots.
