# Supplementary material for: Placental methylome reveals a 22q13.33 brain regulatory gene locus associated with autism
Source: Genome Biol. 2022 Feb 16;23:46. doi: 10.1186/s13059-022-02613-1 (PMC8848662; doi:10.1186/s13059-022-02613-1)
Supplement: Supplementary file 32 — Additional file 32. Review history. [file 13059_2022_2613_MOESM32_ESM.docx]

Review History

**First round of review**

**Reviewer 1**

**Are you able to assess all statistics in the manuscript, including the appropriateness of statistical tests used?**

Yes, and I have assessed the statistics in my report.

**Comments to author:**

Thank you very much for the opportunity to review this paper.

Zhu et al. first perform differentially methylated regional analysis of placental samples, where they find 22q13.33 is hypomethylated in the ASD group (replicating these in their "internal" and "external" replication sets). They take this as a starting point to further characterise the region. Specifically, they perform some functional characterisation of the gene NHIP; look for common structural variation in the locus associated with NHIP for which they find an SV insertion in 22q13.33 also associated with ASD risk. They turn to post-mortem brain tissue analyses and cell culture experiments to investigate tissue-specific relationships between this 22q13.33 insertion, NHIP expression, and relationships with other genes.

There are some notable strengths to this study

* The prospective design of MARLES and EARLI is important to the autism field.

* The authors make a commendable effort to draw together and integrating disparate datasets and models (multiple modalities of omics data from placental tissue, post-mortem brain tissue, and cell-lines) to support their claims.

Nevertheless, there are some concerns that I have about the manuscript that I believe should be addressed. A particularly concerning general issue is one of clarity and providing key details. This makes it difficult to properly assess the strength of evidence in this work. I have attempted to dig through long Supplementary material, but even then in many cases, the answers are still not found there.

Major points:

* At current, the main text lacks critical detail with which to assess the importance of the authors' findings. Almost every claim (and especially where the word "significant" is stated) should be accompanied by what statistical test was used, an effect size, standard error, p-value, and what N were included). Without these statistics, I also have great difficulty in assessing whether multiple testing correction was adequate (which must be done). In some cases, (like Supplementary Figure 30, Fisher's test, which is used to claim that there is significant overlap of NHIP-associated transcripts with SFARI ASD risk genes) the null distribution is unclear (this is an important consideration). Please also consider all Supplementary Figure legends when adding these necessary statistical details.

* Covariates included in each analysis should be clearly stated. In some analyses, it is unclear whether they are included at all when they should be to avoid confounding. I find it difficult to find any information of the age distribution between groups and cohorts. Specifically for making the statement that "Methylation levels within the 134 ASD DMRs were specifically associated with autism severity and cognitive scores, but not other demographic and technical variables", this would more robustly be tested by fitting a linear model with the other variables in Supp Fig 3 as covariates, and then seeing if the diagnosis term of the model retains significance. For example, there appears to be a subset of probes in the Supp Fig 3 heatmap that have a comparable relationship with ASD traits as there are with the last 4 variables (eg. CpG methylation %), which may create a spurious relationship.

* Furthermore, this study draws upon a variety of datasets (in-house and public), and it is often unclear which corresponds to each analysis. The dataset corresponding to each analysis should be clearly stated where each of these came from. In the case of cell lines, it would be most helpful if the derivative cell-type was also stated. For human cohorts (age, sex, diagnosis), the manuscript would benefit from a brief description of the demographics to allay concerns about confounding.

* While the inclusion of exhaustive supplementary tables and figures are commendable, clarity would be greatly improved by providing a brief summary of the result, rather than simply providing a reference.

* "Replication" groups and sequencing platforms: at current the effect of batch/sequencing platform is downplayed, despite there being huge effects in Supp Fig 2. This is potentially a significant limitation of the study, and must be described upfront, rather than leaving the reader to dig through Supplementary for it. What is meant by "sequencing platform" - do you mean "batch" instead? Also, what were the relationship between batches and group characteristics (e.g., I see that the "internal replication" group was the only group with non-TD participants)? I appreciate there is a table in Supp Table 1, but some comment on differences should be included in the main text. Furthermore, the terminology of "internal replication sample" is confusing, implying that assays were reperformed on the same samples.

* Detection of DMRs: please note that the DMRichR documentation states that the number of permutations should not increase the number of samples. I believe that your total discovery set has fewer than 100 people in it.

* The links between experiments sometimes seem tenuous. For example, if I take the following statement at face value "gene body methylation in placenta predicts active gene expression" (this statement also needs a reference), that doesn't mean that the fetus will also have active gene expression in this locus, despite that being stated in the next sentence. Also, I would expect gene body methylation to be associated with reduce gene expression (rather than "active"). Secondly, I think that the rationale for studying hypoxia could be "sold" more (e.g., surely many other conditions affect both neuronal and placental trophoblast differentiation? How specific is hypoxia to processes shared across these tissues?).

* Relationship with prenatal vitamin use: I believe it is important to state that the reference [43] provided is also from the same MARBLES cohort, rather than that epidemiological finding being independent of this cohort. I would also tone down the causal wording regarding this finding (eg, "nutrient intervention with demonstrated ASD protection", and also similar statements on page 13, lines 22-31), as the finding is purely associative. I would also note that it may be useful to describe the relationship between prenatal vitamin use and folic acid (as is done in reference [43]), as the former is not a recognised risk factor for ASD, whereas folic acid has a more robust relationship (see Figure 2, Lord et al. 2020 Nature Reviews Primers).

* SV insertion in 22q13.33 within these samples (MARBLES and post-mortem brain): I would like more explicit information on the characteristics of this region between participants. Eg. how many had 0, 1, and 2 alleles, rather than just homozygous vs non-homozygous? What is the ancestry break down? Is there any potential confounding between ancestry and the genetic associations you find? Could the ASD/TD allele frequency differences for the SV insertion be related to ancestry? What is the allele frequency in gnomAD (ie. for INS_22_115103), and how different is it across ancestries? Also, in terms of identifying this SV relationship, what were the specific criteria for including "common variants" in 22q13.33 in analysis (line 17, page 11)?

* For the NHIP-associated gene analysis on page13-14: Did you include covariates in this analysis? I strongly believe that covariates should be included as they may be important confounders. Also, on Page 14, line 9, it would probably be clearer to say "brain samples as a continuous response variable", if my interpretation is correct

* For the "significant overlap of 284 genes" on page 15, line 53, the way in which significance is assessed is unclear. A useful comparison to assess consistency of signal would be to see which/any GO terms overlap between the in vivo and in vitro analyses (as opposed to simply combining them together, as is currently done).

Minor points

* The figures could also benefit from more descriptive axis labels so that the reader is less wholly dependent on the figure legends to understand the figures

* It would help the reader to rationalise the focus on 22q13.33 if you mentioned that mentioned that this was the *only* genome-wide locus in the initial DMR analysis

**Reviewer 2**

**Are you able to assess all statistics in the manuscript, including the appropriateness of statistical tests used?**

No, I do not feel adequately qualified to assess the statistics.

**Comments to author:**

The study by Zhu and colleagues uses data from 2 cohorts (split into discovery, internal and external validation sets) to examine WGBS methylation data in 204 ASD cases and controls. They discover a novel locus on chromosome 22 that is not well covered by either methylation or genotype arrays. They use a variety of downstream approaches to interrogate the functional implications of this region.

Overall, there are a huge number of analyses reported in the paper. Whereas my background is in array-based statistical genetics, I find the triangulation of evidence fairly compelling. I have a few specific comments.

1. Page 3: "ASD genetic susceptibility predictions can be improved by adding single nucleotide polymorphism (SNP) weights using polygenic risk scores (PRS) from ASD-correlated traits, including schizophrenia, depression, and educational attainment"

I think you could delete "single nucleotide polymorphism (SNP) weights using" or maybe rephrase the sentence. Although I understand what you mean, I don't think the current version is clear.

2. Page 3: "For example, a schizophrenia PRS was shown to be more than five times greater in the presence of early-life maternal complications"

Five times greater than what? I also had a look at the cited manuscript but was unable to find this result - is the citation correct? I might also re-order the sentence to note that for those with a record of an early-life maternal complication, the mean (?) SCZ PRS was >5 times greater than those without such a complication.

3. Page 11 (Supplementary Figure 21), Page 12 (Supplementary Figure 26), Page 13 (Fig 4a).

These results are either borderline significant or based on extremely small n's - often two or three individuals are driving the findings e.g Fig 4a. At the very least this requires a major caveat in the results and discussion. Was multiple testing controlled for across all (independent) experiments/analyses?

4. Page 24: Please could you provide more details about the permutation testing.

5. Page 27: Please could you clarify the ANOVA models here? The PRS is a continuous variable and you compare means across diagnosis groups (although it is a 2 category outcome so equivalent to a t-test?) but it is unclear to me how the % block methylation is being assessed.

6. While you critique the EPIC array for lacking coverage in the 22q13.33 block, there are several CpGs present, as shown in Figure S13. Did you check to see if any of these sites (DMPs and DMRs) were associated with case/control status in the MARBLES cohort where both EPIC and WGBS data are available? I think these results and the integration of larger-scale EPIC/450k EWAS results from the literature would be very useful.

**Authors Response**

**Point-by-point responses to the reviewers’ comments:**

Thank you very much for the opportunity to review this paper.

Zhu et al. first perform differentially methylated regional analysis of placental samples, where they find 22q13.33 is hypomethylated in the ASD group (replicating these in their "internal" and "external" replication sets). They take this as a starting point to further characterise the region. Specifically, they perform some functional characterisation of the gene NHIP; look for common structural variation in the locus associated with NHIP for which they find an SV insertion in 22q13.33 also associated with ASD risk. They turn to post-mortem brain tissue analyses and cell culture experiments to investigate tissue-specific relationships between this 22q13.33 insertion, NHIP expression, and relationships with other genes.

There are some notable strengths to this study

*	The prospective design of MARLES and EARLI is important to the autism field.

*	The authors make a commendable effort to draw together and integrating disparate datasets and models (multiple modalities of omics data from placental tissue, post-mortem brain tissue, and cell-lines) to support their claims.

Nevertheless, there are some concerns that I have about the manuscript that I believe should be addressed. A particularly concerning general issue is one of clarity and providing key details. This makes it difficult to properly assess the strength of evidence in this work. I have attempted to dig through long Supplementary material, but even then in many cases, the answers are still not found there.

Major points:

*	At current, the main text lacks critical detail with which to assess the importance of the authors' findings. Almost every claim (and especially where the word "significant" is stated) should be accompanied by what statistical test was used, an effect size, standard error, p-value, and what N were included). Without these statistics, I also have great difficulty in assessing whether multiple testing correction was adequate (which must be done). In some cases, (like Supplementary Figure 30, Fisher's test, which is used to claim that there is significant overlap of NHIP-associated transcripts with SFARI ASD risk genes) the null distribution is unclear (this is an important consideration). Please also consider all Supplementary Figure legends when adding these necessary statistical details.

*Response: We thank the reviewer for their rigorous attention to these important details. We have thoroughly revised the manuscript text and figure legends of the main and supplementary figures to include the statistical test and other important details of sample size and statistics.*

*	Covariates included in each analysis should be clearly stated. In some analyses, it is unclear whether they are included at all when they should be to avoid confounding. I find it difficult to find any information of the age distribution between groups and cohorts. Specifically for making the statement that "Methylation levels within the 134 ASD DMRs were specifically associated with autism severity and cognitive scores, but not other demographic and technical variables", this would more robustly be tested byfitting a linear model with the other variables in Supp Fig 3 as covariates, and then seeing if the diagnosis term of the model retains significance. For example, there appears to be a subset of probes in the Supp Fig 3 heatmap that have a comparable relationship with ASD traits as there are with the last 4 variables (eg. CpG methylation %), which may create a spurious relationship.

*Response: We have now performed linear regression models including all of the potential covariates shown in Supplemental Figure 3. The new Supplemental Figure 3b shows the results of these linear models performed for each of the 134 ASD DMRs and the new Supplemental Figure 3c shows the adjusted p values for smoothed methylation over the 22q block, which demonstrates that diagnosis is the strongest predictor of the association, not the other variables. We have also revised the statement of Results to better reflect the actual results shown in the revised Supplemental Figure 3.*

*	Furthermore, this study draws upon a variety of datasets (in-house and public), and it is often unclear which corresponds to each analysis. The dataset corresponding to each analysis should be clearly stated where each of these came from. In the case of cell lines, it would be most helpful if the derivative cell-type was also stated. For human cohorts (age, sex, diagnosis), the manuscript would benefit from a brief description of the demographics to allay concerns about confounding.

*Response: We have carefully edited the text of the Results section to incorporate more details and clarity about the dataset corresponding to each analysis. This includes more detail about the demographic and covariate information in Supplemental Tables 1 and 2, the source of the cell lines, and the tissues analyzed for RNAseq.*

*	While the inclusion of exhaustive supplementary tables and figures are commendable, clarity would be greatly improved by providing a brief summary of the result, rather than simply providing a reference.

*Response: We have expanded the text in the Results section to provide brief summaries of the results in the Supplemental Tables and Figures.*

*	"Replication" groups and sequencing platforms: at current the effect of batch/sequencing platform is downplayed, despite there being huge effects in Supp Fig 2. This is potentially a significant limitation of the study, and must be described upfront, rather than leaving the reader to dig through Supplementary for it. What is meant by "sequencing platform" - do you mean "batch" instead? Also, what were the relationship between batches and group characteristics (e.g., I see that the "internal replication" group was the only group with non-TD participants)? I appreciate there is a table in Supp Table 1, but some comment on differences should be included in the main text. Furthermore, the terminology of "internal replication sample" is confusing, implying that assays were reperformed on the same samples.

*Response: The differences in methylation levels in Supplemental Figure 2 are due to technology differences in the Illumina sequencer that was used (HiSeq 4000, HiSeq X, NovaSeq). While this was a necessary limitation of the study because of the change in technologies over the time period when samples were collected and analyzed, it is also a strength because it shows that the main finding of hypomethylation over the 22q block in ASD replicated, in spite of the sequencing platform used. To clarify this point, we have expanded the description of the sequencing platforms in Supplemental Figure 2 and the text of the Results. We have also changed the wording of “internal replication” to “specificity replication” because that group was actually testing the specificity of the methylation signature in the 22q13.33 block to distinguish ASD from non-TD and TD diagnostic categories.*

*	Detection of DMRs: please note that the DMRichR documentation states that the number of permutations should not increase the number of samples. I believe that your total discovery set has fewer than 100 people in it.

*Response: We repeated the DMR detection using 90 permutations (our discovery set was 92 individuals). We identified the same 134 DMRs as in the 100 permutations test from the original manuscript. The main findings of 12 ASD DMRs mapping to 22q13.33, including the one which passed genome-wide significance also replicated with 90 permutations. We updated Supplementary Table 3 to include the results using either 100 or 90 permutations and the text of the Methods to explain this approach.*

*	The links between experiments sometimes seem tenuous. For example, if I take the following statement at face value "gene body methylation in placenta predicts active gene expression" (this statement also needs a reference), that doesn't mean that the fetus will also have active gene expression in this locus, despite that being stated in the next sentence. Also, I would expect gene body methylation to be associated with reduce gene expression (rather than "active"). Secondly, I think that the rationale for studying hypoxia could be "sold" more (e.g., surely many other conditions affect both neuronal and placental trophoblast differentiation? How specific is hypoxia to processes shared across these tissues?).

*Response: We have expanded the text in the Introduction and Results to better explain and reference the findings of gene body methylation in placenta and the importance of hypoxia sensitivity of placental trophoblast cells as well as neuronal differentiation and maturation.*

*	Relationship with prenatal vitamin use: I believe it is important to state that the reference [43] provided is also from the same MARBLES cohort, rather than that epidemiological finding being independent of this cohort. I would also tone down the causal wording regarding this finding (eg, "nutrient intervention with demonstrated ASD protection", and also similar statements on page 13, lines 22-31), as the finding is purely associative. I would also note that it may be useful to describe the relationship between prenatal vitamin use and folic acid (as is done in reference [43]), as the former is not a recognised risk factor for ASD, whereas folic acid has a more robust relationship (see Figure 2, Lord et al. 2020 Nature Reviews Primers).

*Response: We agree that it might be important to note that prenatal vitamins are folic acid supplements and the main source of folic acid in the US during pregnancy. We added this information to the text along with our rationale for examining them as a methyl donor source. We also added references to other findings specific to prenatal vitamin intake in the first month of pregnancy in relation to reduced ASD diagnosis. We agree that these epidemiologic findings should be discussed in a non-causal frame and have adjusted our language to reflect the associative nature of the findings.*

*	SV insertion in 22q13.33 within these samples (MARBLES and post-mortem brain): I would like more explicit information on the characteristics of this region between participants. Eg. how many had 0, 1, and 2 alleles, rather than just homozygous vs non-homozygous? What is the ancestry break down? Is there any potential confounding between ancestry and the genetic associations you find? Could the ASD/TD allele frequency differences for the SV insertion be related to ancestry? What is the allele frequency in gnomAD (ie. for INS_22_115103), and how different is it across ancestries? Also, in terms of identifying this SV relationship, what were the specific criteria for including "common variants" in 22q13.33 in analysis (line 17, page 11)?

*Response: We have clarified the location of the allelic genotype information in the revised Results section (Supplementary Table 12 for placenta samples, Supplementary Table 14 for brain samples). These Supplemental Tables also include any race and ethnicity information that was available. Genetic ancestry was not available for these samples. Because the allele frequency is high in all populations in the gnomAD database (0.7, Supplementary Figure 25) and does not vary by more than 10% between any ancestry group, we do not think that the ASD/TD allele frequency differences we observed was driven by ancestry. However, in response to this concern, we have added more information about race/ethnicity of the samples to the text of the Results section and the supplementary tables. The criteria we used for including SNPs or structural variants in the analyses shown in Figure 3b was filterer for minor allele frequency > 5%. We have added these criteria to the method section.*

*	For the NHIP-associated gene analysis on page13-14: Did you include covariates in this analysis? I strongly believe that covariates should be included as they may be important confounders. Also, on Page 14, line 9, it would probably be clearer to say "brain samples as a continuous response variable", if my interpretation is correct

*Response: We appreciate this comment and have revised the analyses in Figure 4 which now contains data from brain RNAseq differential gene analysis adjusted for sex, age, brain region, and postmortem interval. Since race/ethnicity was matched between samples and the small numbers of available brain samples was a limitation, we could not adjust for these. The list of NHIP associated genes following adjustments for these potential brain sample covariates are in Supplementary Table 16, the list of GO terms for these genes is in Supplementary Table 17, and their overlap with SFARI genes is in Supplementary Table 18. The list of NHIP associated genes without adjustments remains in Supplementary Table 15 for comparison. Supplementary Fig. 29 shows that there was a significant overlap in brain NHIP associated genes with those of the unadjusted analysis (Fisher’s exact test, p-value < 2.2E-16, OR = 248.454). While the overall results were quite similar, based on the reviewer’s strong opinion about adjustment, we decided to use the gene list for the adjusted data in the revised Figure 4 and Table 1. We have also corrected the phrase to read "brain samples as a continuous response variable".*

*	For the "significant overlap of 284 genes" on page 15, line 53, the way in which significance is assessed is unclear. A useful comparison to assess consistency of signal would be to see which/any GO terms overlap between the in vivo and in vitro analyses (as opposed to simply combining them together, as is currently done).

*Response: We reworded this sentence to make it clear that the null comparison group for the Fisher’s exact test was all expressed genes (defined in the Methods section). We have also added this information to the Supplementary Figure legends. We also performed the suggested GO term overlap between the in vivo and in vitro analyses and the results are in Supplementary Table 25.*

Minor points

*	The figures could also benefit from more descriptive axis labels so that the reader is less wholly dependent on the figure legends to understand the figures

*Response: We have added additional information to the labels on the main figures.*

*	It would help the reader to rationalise the focus on 22q13.33 if you mentioned that mentioned that this was the *only* genome-wide locus in the initial DMR analysis

*Response: We reworded the justification for focus on 22q13.33 in the Results section.*

Reviewer #2: The study by Zhu and colleagues uses data from 2 cohorts (split into discovery, internal and external validation sets) to examine WGBS methylation data in 204 ASD cases and controls. They discover a novel locus on chromosome 22 that is not well covered by either methylation or genotype arrays. They use a variety of downstream approaches to interrogate the functional implications of this region.

Overall, there are a huge number of analyses reported in the paper. Whereas my background is in array-based statistical genetics, I find the triangulation of evidence fairly compelling. I have a few specific comments.

1.	Page 3: "ASD genetic susceptibility predictions can be improved by adding single nucleotide polymorphism (SNP) weights using polygenic risk scores (PRS) from ASD-correlated traits, including schizophrenia, depression, and educational attainment"

I think you could delete "single nucleotide polymorphism (SNP) weights using" or maybe rephrase the sentence. Although I understand what you mean, I don't think the current version is clear.

*Response: We agree with the reviewer that the wording here could use improvement. We have re-written this section as follows:*

*“A large genome-wide association study (GWAS) identified individual genetic variants that contribute to an individual’s ASD risk, and showed that the weighed sum of the risk alleles and their effect sizes can be combined to create a polygenic risk score (PRS) for ASD [8]. The variance explained for ASD using this approach was 2.45% , which was further improved to 3.77% by including in the prediction model additional PRS for traits co-heritable with ASD, including schizophrenia, depression, and educational attainment [8–10].”*

2.	Page 3: "For example, a schizophrenia PRS was shown to be more than five times greater in the presence of early-life maternal complications"

Five times greater than what? I also had a look at the cited manuscript but was unable to find this result - is the citation correct? I might also re-order the sentence to note that for those with a record of an early-life maternal complication, the mean (?) SCZ PRS was >5 times greater than those without such a complication.

*Response: We apologize that this sentence was not clear, and we realize that the references were not placed in a way that was intuitive for the reader. The interaction between early-life maternal complication and SCZ PRS was identified in reference 12, deriving the SCZ PRS from variants identified in reference 11. We have changed the sentence and it now reads as follows:*

*“Common polygenic risk may also interplay with early environmental and perinatal factors. For example, a PRS derived from a set of 108 previously identified genome-wide significant variants for schizophrenia [11] was shown to be significant only in the presence of early-life maternal complications (ELCs), and the subset of variants interacting with ELCs also corresponded with patterns of placental gene expression, consistent with the importance of placental gene regulation as a window into neurodevelopment [12].”*

3.	Page 11 (Supplementary Figure 21), Page 12 (Supplementary Figure 26), Page 13 (Fig 4a). These results are either borderline significant or based on extremely small n's - often two or three individuals are driving the findings e.g Fig 4a. At the very least this requires a major caveat in the results and discussion. Was multiple testing controlled for across all (independent) experiments/analyses?

*Response: We added a mention of the small number of brain samples to the Results, as well as an additional paragraph to the Discussion to include the limitations of the study. In this section, we specifically mention the small number of available postmortem samples for ASD research as a limitation. However, the number of brain samples included in our study is comparable to other analyses of postmortem brain in the ASD field. We controlled for multiple testing in the discovery-based genomic analyses (WGBS and RNAseq). However, each individual non-genomic experiment, including the one shown in Fig 4a, was designed to test a single specific hypothesis clearly stated in the text of the Results section.*

4.	Page 24: Please could you provide more details about the permutation testing.

*Response: We added additional details about the permutation testing to the Methods.*

5.	Page 27: Please could you clarify the ANOVA models here? The PRS is a continuous variable and you compare means across diagnosis groups (although it is a 2 category outcome so equivalent to a t-test?) but it is unclear to me how the % block methylation is being assessed.

*Response: We added additional details to the Methods about the ANOVA model used for the data in Supplementary Table 13. We also performed similar analyses using the alternate approach of linear regression and obtain very similar results, shown in Supplementary Table 13. Individual smoothed methylation levels within the 22q13.33 block were obtained using bsseq and an approach we describe in more detail in the revised Methods section.*

6.	While you critique the EPIC array for lacking coverage in the 22q13.33 block, there are several CpGs present, as shown in Figure S13. Did you check to see if any of these sites (DMPs and DMRs) were associated with case/control status in the MARBLES cohort where both EPIC and WGBS data are available? I think these results and the integration of larger-scale EPIC/450k EWAS results from the literature would be very useful.

*Response: We did check the EPIC array from the same samples for the few probes that were within the 22q13.33 block. However, there was no significant difference between ASD and TD for any of these probes. This result was not surprising because these probes did not overlap with the 12 DMRs found in WGBS analysis. We also searched the MRC-IEU EWAS catalog (ewascatalog.org) for the region chr22:49440481-49558654. There were 90 reports of 42 CpG sites as significant hits from other studies, but none showing any association with ASD. Because of the poor overlap between these probes and those within our 12 ASD DMRs, we decided not to include this report.*

**Second round of review**

**Reviewer 1**

Thank you very much to Zhu et al. for comprehensively addressing the reviewers’ requests. This is greatly appreciated, and I think that the manuscript is much clearer and stronger to read as a result.

I have a few more questions about the data and analysis that I believe are important to further clarify and strengthen the results:

1. Could you please confirm that sample QC was done separately between cohorts? (Or if QC was done in aggregate, that QC outcomes aren’t dependent on the other samples?) Just to ensure that they are true replication samples, with no “contamination” from the other cohorts?

2. Text relating to checking the relationship between DMRs and demographic and technical covariates and SF3c: Could you please confirm whether this was an analysis of univariable (ie. DMR ~ diagnosis, DMR ~ sex, etc.) or multivariable model (ie. DMR ~ diagnosis + sex + maternal education + home ownership + etc.)? I would like to see a multivariable linear model to see what happens when these effects are jointly modelled (and most particularly the technical, cell-type and sex variables), as I think this will be most convincing in ruling out potential confounding. Additionally, could you please also report these p-values without adjustment? I really appreciate the extensive FDR adjustment you have performed, but in this case, I think it is appropriate to include the raw p-values, as this isn’t a case of testing multiple different hypotheses. The robustness of the downstream results relating to the cohort data (eg. page 9-10, 13-15) would be greatly improved if the nominally-significant variables from this multivariable analysis were included as covariates in a linear model framework, in favour of performing t-tests which don’t account for covariates.

3. Supp Figure 15: could you comment on whether you would expect NHIP to reduce from Day 6 to 8 without CoCl2 treatment (and if not, how that affects interpretation of the decrease seen in the CoCl2 treatment)? And were non-CoCl2-treated cells also washed out at Day 6?

4. I do find the relationship between chr22q13.33 methylation, the insertion and NHIP to be convincing. However, I have less confidence in the interpretation of the results for relationship between prenatal vitamin use and 22q13.33 insertion, discussed on page 15 (per the marked-up version of the manuscript): The stratified result in Supp Fig 26 suggests to me that chr22q block % methylation is primarily driven by insertion genotype (lower % methylation on Supp Figure 26a vs Supp Figure 26b). In this stratified analysis, there are no group differences by prenatal vitamin usage, except for between those with insertion and taking prenatal vitamins. However, I do not find this result wholly convincing as it hinges on only 7 individuals for whom the range of methylation % is within that of the corresponding group without prenatal vitamins intake. Hence, I believe that statements linking prenatal vitamin usage to the chr22q block and broader extrapolation should be caveated to acknowledge this. Eg., “prenatal vitamin use during the first month of pregnancy showed a nominally-significant protective effect among individuals with the insertion, which we acknowledge is small …”

5. Page 19, testing for overlap of DEGs in response to experimental NHIP overexpression and those associated with NHIP transcript levels in human brain: Could you please clarify what is meant by “all expressed genes”. I think that the most appropriate comparison would be with the set of genes that are expressed in both the embryo cell-line *and* the human brain samples, rather than all genes.

Minor points (N.B. page references are per the marked-up version of the manuscript):

Page 2, line 46: statistics are provided twice

Page 3: “Common polygenic risk may also interplay with early environment and perinatal factors in other neurodevelopmental diagnosis. For example …”

Page 5: Could you please briefly explain what CoRSIVs are in the Introduction, just to fill in people who might not be familiar with the concept? (I do see it on page 9, but it would also be helpful I think in the Introduction)

Page 13: Could you clarify which cohorts were included in the structural variant analyses? MARBLES only?

Page 15: would say “nominally significant protective effect”

Page 22: “homozygosity for this 22q13.33 insertion was a better predictor of ASD risk than GWAS-based PRS” – is this a fair comparison? Ie. is the ancestry/race of people included in this analysis matching to the GWAS summary statistics (from Europeans)?

**Reviewer 2**

I thank the authors for their responses, which address most of my comments. However, I think there are still a few outstanding issues.

1. Re the response to point 3: "However, each individual non-genomic experiment, including the one shown in Fig 4a, was designed to test a single specific hypothesis clearly stated in the text of the Results section." you often have multiple tests per Figure/analysis that need to be considered. You also need to use rank-based non-parametric tests (eg Mann Whitney Test instead of T-tests) when n's are so small.

2. Re the response to point 5, there is insufficient justification of the methods. Linear regression is more appropriate than ANOVA for the % block methylation analysis so should be reported as the primary analysis. I don't see why ANOVA should be considered here at all. Also, verbatim posting of R output into a supplementary table is not appropriate. Please format these as proper tables.

3. More of a comment but, in response to point 6, did you also compare the effect sizes between the WGBS and EPIC output to see how consistent they were? I think it's a pity not to include the EWAS and EWAS catalog checks in the paper.

**Authors Response**

**Point-by-point responses to the reviewers’ comments:**

Reviewer #1: Thank you very much to Zhu et al. for comprehensively addressing the reviewers’ requests. This is greatly appreciated, and I think that the manuscript is much clearer and stronger to read as a result.

I have a few more questions about the data and analysis that I believe are important to further clarify and strengthen the results:

1. Could you please confirm that sample QC was done separately between cohorts? (Or if QC was done in aggregate, that QC outcomes aren’t dependent on the other samples?) Just to ensure that they are true replication samples, with no “contamination” from the other cohorts?

*Response: Yes, sample QC was independently performed for each of the three groups (discovery, external replication, and specificity replication). Since these three groups of samples were sequenced on different platforms, all QC was done separately for each group. We have added this statement to the revised Methods.*

2. Text relating to checking the relationship between DMRs and demographic and technical covariates and SF3c: Could you please confirm whether this was an analysis of univariable (ie. DMR ~ diagnosis, DMR ~ sex, etc.) or multivariable model (ie. DMR ~ diagnosis + sex + maternal education + home ownership + etc.)? I would like to see a multivariable linear model to see what happens when these effects are jointly modelled (and most particularly the technical, cell-type and sex variables), as I think this will be most convincing in ruling out potential confounding. Additionally, could you please also report these p-values without adjustment? I really appreciate the extensive FDR adjustment you have performed, but in this case, I think it is appropriate to include the raw p-values, as this isn’t a case of testing multiple different hypotheses. The robustness of the downstream results relating to the cohort data (eg. page 9-10, 13-15) would be greatly improved if the nominally-significant variables from this multivariable analysis were included as covariates in a linear model framework, in favour of performing t-tests which don’t account for covariates.

*Response: Yes, the analysis for Supplementary Fig. 3c used a multivariable linear model and we have clarified this in the figure legend and provided the model used. We have substituted the raw p values for the corrected ones in the revised Supplementary Fig. 3c. Diagnosis was the strongest predictor of the association (p=0.002). We also adjusted for the potential effects of the two nominal covariates, birthweight and nRBC, in the new Supplementary Fig. 9 (corresponding to the main Fig. 1e but with adjustments), with actual values in the new Supplementary Table 9. These adjustments did not change the degree of significance of the results shown in Figure 1e.*

3. Supp Figure 15: could you comment on whether you would expect NHIP to reduce from Day 6 to 8 without CoCl2 treatment (and if not, how that affects interpretation of the decrease seen in the CoCl2 treatment)? And were non-CoCl2-treated cells also washed out at Day 6?

*Response: Thanks for pointing out the lack of clarity with the figure legend for Supplemental Figure 16. We have revised the legend to clarify that all cells were washed at Day 6. We also now include the statistical tests of the Day 6 to Day 8 differences in NHIP transcript levels: “NHIP transcript level remained unchanged between Day 6 and Day 8 in the mock treatment cells (two-tailed t-test, p-value = 0.410), while differentiated LUHMES treated with CoCl2 at Day 6 had a significantly higher level of NHIP compared with 48 hours after CoCl2 removal on Day 8 (two-tailed t-test, p-value = 0.005).”*

4. I do find the relationship between chr22q13.33 methylation, the insertion and NHIP to be convincing. However, I have less confidence in the interpretation of the results for relationship between prenatal vitamin use and 22q13.33 insertion, discussed on page 15 (per the marked-up version of the manuscript): The stratified result in Supp Fig 26 suggests to me that chr22q block % methylation is primarily driven by insertion genotype (lower % methylation on Supp Figure 26a vs Supp Figure 26b). In this stratified analysis, there are no group differences by prenatal vitamin usage, except for between those with insertion and taking prenatal vitamins. However, I do not find this result wholly convincing as it hinges on only 7 individuals for whom the range of methylation % is within that of the corresponding group without prenatal vitamins intake. Hence, I believe that statements linking prenatal vitamin usage to the chr22q block and broader extrapolation should be caveated to

acknowledge this. Eg., “prenatal vitamin use during the first month of pregnancy showed a nominally-significant protective effect among individuals with the insertion, which we acknowledge is small …”

*Response: We have modified this statement in the revised text, based reviewer’s suggestions.*

5. Page 19, testing for overlap of DEGs in response to experimental NHIP overexpression and those associated with NHIP transcript levels in human brain: Could you please clarify what is meant by “all expressed genes”. I think that the most appropriate comparison would be with the set of genes that are expressed in both the embryo cell-line *and* the human brain samples, rather than all genes.

*Response: We used all genes expressed in at least one read in any sample, stated in page 39 of the Methods section. In response to this concern, we have repeated the significance test for all genes with expression in both tissues and have added both results to p. 19: “>1 read in any sample, Fisher’s exact test, p-value = 6.946E-5, OR = 1.482; or >1 read per sample in both tissues, Fisher’s exact test, p-value = 8.348E-4, OR = 1.386.”*

Minor points (N.B. page references are per the marked-up version of the manuscript):

Page 2, line 46: statistics are provided twice

*Response: Modified*

Page 3: “Common polygenic risk may also interplay with early environment and perinatal factors in other neurodevelopmental diagnosis. For example …”

*Response: Modified*

Page 5: Could you please briefly explain what CoRSIVs are in the Introduction, just to fill in people who might not be familiar with the concept? (I do see it on page 9, but it would also be helpful I think in the Introduction)

*Response: The third paragraph of the Introduction contains this description of CoRSIVs:*

*“Most epigenome-wide association studies (EWAS) for ASD have used array-based methods to assess DNA methylation which lack coverage over the most epigenetically and genetically polymorphic regions of the human genome, such as correlated regions of systemic interindividual variation (CoRSIVs) and structural variants (SVs) [24]. CoRSIVs are sensitive to periconceptional environment, observed across diverse tissues, associated with human disease genes, and are enriched for transposable elements and subtelomeric locations [24,25]. “*

Page 13: Could you clarify which cohorts were included in the structural variant analyses? MARBLES only?

*Response: The structural variant analyses were only performed in the MARBLES discovery group. We now clarify this in the Methods section: “The discovery group included 92 samples (ASD n = 46, TD n = 46) from the MARBLES study that were used for both WGBS and WGS.”*

Page 15: would say “nominally significant protective effect”

*Response: Modified*

Page 22: “homozygosity for this 22q13.33 insertion was a better predictor of ASD risk than GWAS-based PRS” – is this a fair comparison? Ie. is the ancestry/race of people included in this analysis matching to the GWAS summary statistics (from Europeans)?

*Response: We have added the clause “…in this mixed ancestry cohort.” to the end of this sentence.*

Reviewer #2: I thank the authors for their responses, which address most of my comments. However, I think there are still a few outstanding issues.

1. Re the response to point 3: “However, each individual non-genomic experiment, including the one shown in Fig 4a, was designed to test a single specific hypothesis clearly stated in the text of the Results section.” You often have multiple tests per Figure/analysis that need to be considered. You also need to use rank-based non-parametric tests (eg Mann Whitney Test instead of T-tests) when n’s are so small.

*Response: Thanks for clarifying this point. We have now performed a Mann Whitney Wilcoxon test for the results in Fig. 4a and included the revised p values in the Fig 4a legend. These changes do not modify the conclusions from this figure.*

2. Re the response to point 5, there is insufficient justification of the methods. Linear regression is more appropriate than ANOVA for the % block methylation analysis so should be reported as the primary analysis. I don’t see why ANOVA should be considered here at all. Also, verbatim posting of R output into a supplementary table is not appropriate. Please format these as proper tables.

*Response: We now only show the linear regression results in Supplementary Table 14 and have reformatted this revised table for improved clarity.*

3. More of a comment but, in response to point 6, did you also compare the effect sizes between the WGBS and EPIC output to see how consistent they were? I think it's a pity not to include the EWAS and EWAS catalog checks in the paper.

*Response: We appreciate the reviewer’s interest in EWAS overlaps. But there was insufficient coverage of EPIC probes over the locus to make any meaningful interpretation about effect sizes. We are actively working on additional manuscripts that will compare EPIC placenta data across MARBLES and EARLI cohorts, but significant ASD associations have not been found to date by EPIC analyses in our cohorts.*

**Third round of review**

**Reviewer 1**

Thank you to Zhu et al. for comprehensively addressing my comments. My only remaining suggestion would be to define what CoRSIVs represent genetically, as the current Introduction text does not seem to describe the essence of this form of epigenetic variation (eg. you could consider something more like "CoRSIVs, which are regions of the genome with similar DNA methylation patterns across tissue types ..."). Thank you again for your interesting work.

**Reviewer 2**

I thank the authors' for their responses. In relation to the non-parametric testing, this needs to be applied throughout the paper and not just in the specific instance that I highlighted. I apologise if this was unclear. It is highly unlikely that the assumptions of t-tests will be met when n's are so small. There was also no response to my query about multiple testing across sub-groups.

**Authors Response**

**Point-by-point responses to the reviewers’ comments:**

Reviewer #1: Thank you to Zhu et al. for comprehensively addressing my comments. My only remaining suggestion would be to define what CoRSIVs represent genetically, as the current Introduction text does not seem to describe the essence of this form of epigenetic variation (eg. you could consider something more like "CoRSIVs, which are regions of the genome with similar DNA methylation patterns across tissue types ..."). Thank you again for your interesting work.

*Response: We have added this phrase to the Introduction.*

Reviewer #2: I thank the authors' for their responses. In relation to the non-parametric testing, this needs to be applied throughout the paper and not just in the specific instance that I highlighted. I apologise if this was unclear. It is highly unlikely that the assumptions of t-tests will be met when n's are so small. There was also no response to my query about multiple testing across sub-groups.

*Response: We have now replaced all statistical results from paired t-tests with those from the non-parametric Mann-Whitney-Wilcoxon tests in the Results and Figure legends describing Figures 1e, 2b-f, 3c-d, 4a, S15, S16, and S27. This altered the actual p values slightly but did not change any of the conclusions. For the question about multiple testing, we did not perform correction for these figures in which a few planned specific hypotheses were being tested, which is standard in the field of molecular genetics.*
